# Acquiring Focus on Paramagnetic Single-Atom Sites with Fast Magic-Angle Spinning NMR

**DOI:** 10.1021/jacs.5c20153

**Published:** 2026-02-14

**Authors:** Ioannis Mylonas-Margaritis, Zhehao Huang, Niklas Hedin, Aleksander Jaworski

**Affiliations:** † Department of Chemistry, 7675 Stockholm University, SE-106 91 Stockholm, Sweden; ‡ Wallenberg Initiative Materials Science for Sustainability, Department of Chemistry, 7675 Stockholm University, SE-106 91 Stockholm, Sweden

## Abstract

A new approach for characterizing paramagnetic sites in materials is introduced. It combines broadband fast magic-angle spinning (MAS) NMR data with *ab initio* computed paramagnetic NMR shifts using correlated wave functions. This study presents a challenging example of this. With Fe coordinated in a model compound, the PCN-224 porphyrin metal–organic framework (Fe@PCN-224 MOF) was used to elucidate the coordination geometry and electronic structure using ^1^H and ^13^C MAS NMR spectra of the ligand atoms. The computationally predicted ^13^C NMR shifts on the paramagnetic Fe@PCN-224 MOF compared unprecedentedly well with experimental ^13^C NMR shifts and equally well for the diamagnetic counterpart, the Fe-free PCN-224 MOF. This is despite the 25 times wider NMR shift range of 1200 ppm for the paramagnetic Fe@PCN-224 MOF. We conclude that this approach is applicable to crystalline, noncrystalline, and molecular systems.

## Graphical abstract

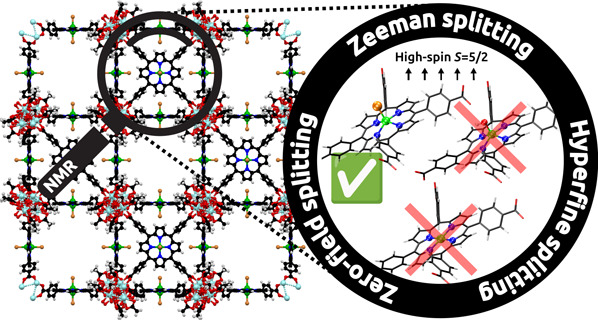

Paramagnetic ions of iron in proteins,^1–4^ catalysts,^5–8^ and other functional materials are essential for life and in chemistry. Currently, the valence state of iron is determined by Mössbauer spectroscopy and X-ray or electron spectroscopy;^4,9–11^ however, there are advantages to using a combination of solid-state fast magic-angle spinning (MAS) ^1^H and ^13^C nuclear magnetic resonance (NMR) data with *ab initio* computed paramagnetic or diamagnetic NMR shifts. Not only is the valence state resolved but also, as we show here, the local geometry of the organic moieties and the single-site Fe ion can be resolved with very high accuracy. Unlike extended X-ray absorption fine structure (EXAFS), which provides a similar level of information but requires the use of beamlines, this method does not. Here, we used a specific Zr­(IV)-oxo-porphyrin metal–organic framework (MOF), the Fe@PCN-224 MOF,^12–17^ as a model system, with the crystal structure (Figure 1a) and geometry-optimized models (Figure 1b) displayed. The PCN-224 MOF was composed of large tetrakis­(4-carboxyphenyl)­porphyrin (TCPP) linkers and Zr­(IV)-6-oxo [Zr_6_O_4_(OH)_4_] secondary building units (SBUs). Such MOFs and related graphitic carbon nitride (g-C_3_N_4_)^18–23^ have been studied as single-atom catalysts (SACs), where the catalytic action is directly related to the interactions between the metal ion and electron-donating ligand nitrogen or oxygen atoms. A detailed understanding of the local coordination environments of metal ions in SACs is often incomplete because of the limitations of the available characterization techniques. In this study, solid-state NMR spectroscopy was used to probe the local geometries and structures. In relation to SACs, it can be mentioned that NMR spectra from diamagnetic species such as ^195^Pt can be measured directly;^24^ however, most metal ions used in catalysis are paramagnetic and cannot be detected directly because of strong electron–nucleus spin–spin hyperfine couplings. These interactions are weaker for the nuclear spins of ligand atoms, and their NMR signals can be detected with modern fast MAS NMR techniques^25–28^ and translated into rich structural and chemical data using accurate electronic structure calculations.^29^ The obtained structural insights were compared to the existing X-ray (averaged) structure of the Fe@PCN-224 MOF.

**Figure 1:**
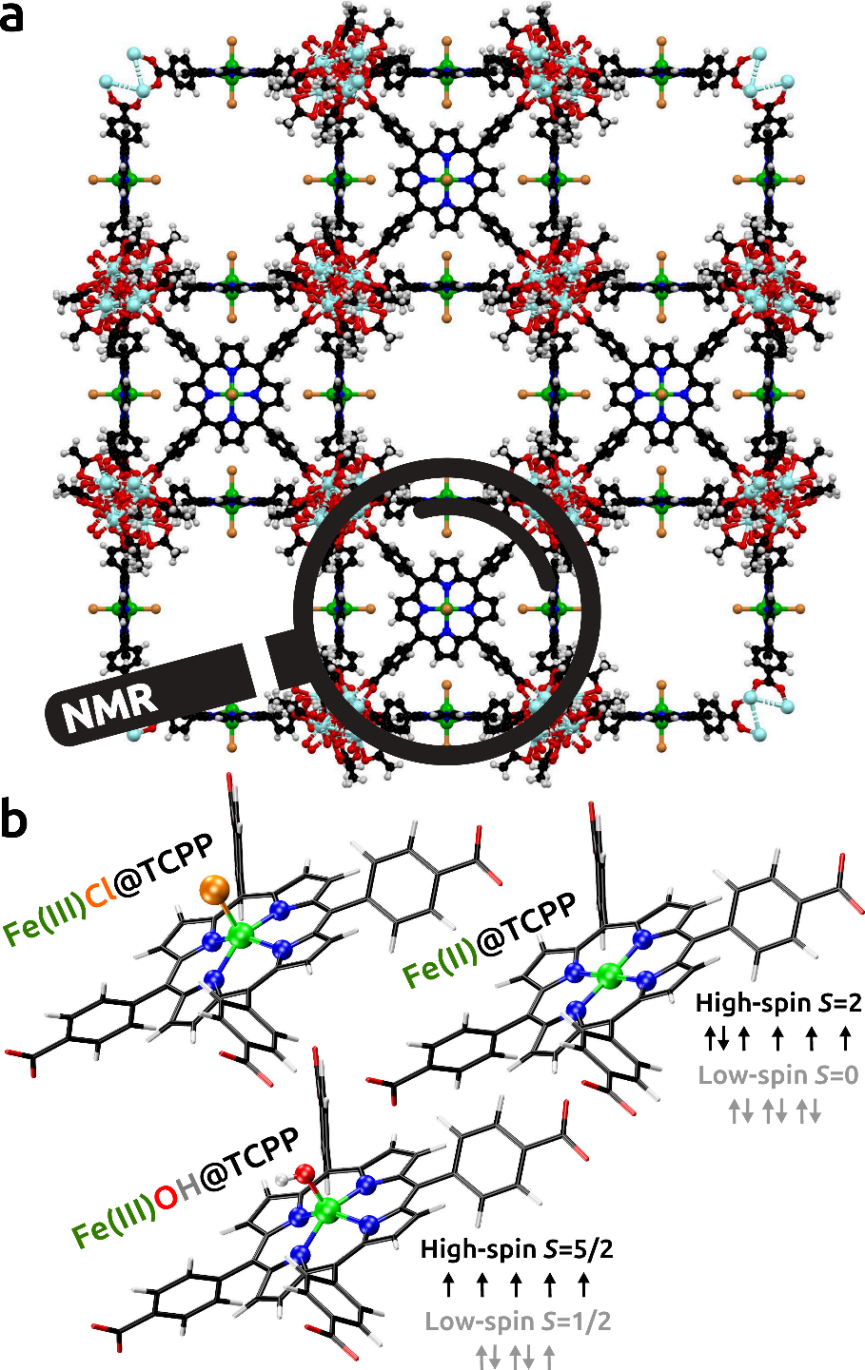
(a) Crystal structure of Fe@PCN-224 MOF generated from crystallographic data by Harris et al.;^16^ note double occupations of Fe positions. (b) Geometry-optimized models of high-spin Fe­(III) five-coordinated and Fe­(II) four-coordinated complexes with the TCPP linker.

The solid-state ^1^H MAS and ^13^C CPMAS NMR spectra of TCPP and the as-synthesized Fe-free PCN-224 MOF (Figure 2a) are characteristic of porphyrin linkers. The Fe-free PCN-224 MOF has orbital shielding (**σ**_orb_) as the only contribution to the NMR chemical shifts.^30–36^ The complete assignment of the ^13^C NMR signals from TCPP (Figure 2b) was based on ^13^C chemical shift predictions with perturbatively corrected DFT,^37^ and an equally robust interpretation was also obtained by *ab initio* calculations with second-order Møller–Plesset perturbation theory (DLPNO-MP2);^38,39^ see the Supporting Information for more details. Analogous ^13^C signal patterns were observed for TCPP molecules grafted onto g-C_3_N_4_,^40^ and in the previously reported spectrum of the PCN-224 MOF.^41^ However, in the latter study, the assignment of the carbon *β* signal was likely incorrect according to the older (liquid-state) NMR literature^42^ and our theoretical shift predictions (section S4 of the Supporting Information). In the ^1^H MAS NMR spectrum of the PCN-224 MOF (inset in Figure 2a), the signal of the NH protons from inside the porphyrin ring can be observed at −3.2 ppm, which indicates that the as-synthesized PCN-224 MOF has no metal ions in the coordination cavity (Figure 2b). The Fe-free PCN-224 MOF contains DMF and acetic acid molecules, the latter of which were shown to be coordinated to the SBUs according to X-ray diffraction data refinements by Harris and co-workers.^16^

**Figure 2:**
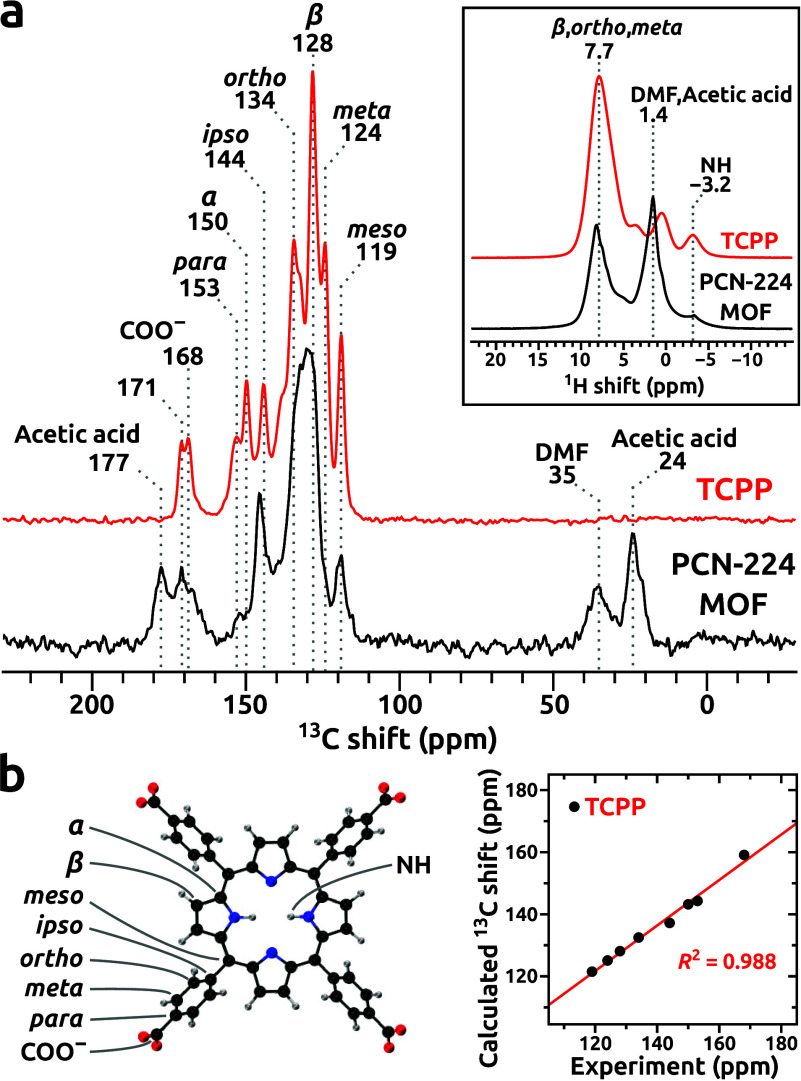
Solid-state ^1^H–^13^C CPMAS (^1^H MAS in the inset) NMR spectra (14.1 T, 60 kHz MAS) of the TCPP linker and PCN-224 MOF (a). Geometry-optimized model of TCPP and fitted linear regression analysis between the observed and calculated (DLPNO-DSD-PBEP86/pcSseg-1) ^13^C NMR shifts (b).

The solid-state ^1^H and ^13^C MAS NMR spectra of the Fe@PCN-224 MOF (Figure 3) were collected at a magnetic field of 14.1 T using a 1.3 mm rotor at MAS rate of 60 kHz and a rotor-synchronized double adiabatic spin–echo pulse sequence.^43,44^ Direct detection of the complete set of ^1^H and ^13^C NMR signals at natural isotope abundance from all atomic positions within the coordination environment of paramagnetic Fe ion is demonstrated, notwithstanding a span of isotropic NMR shifts of 70 ppm for ^1^H and more than 1200 ppm for ^13^C. Previous NMR studies on paramagnetic MOFs have targeted small linkers subjected to the paramagnetism of metal clusters (SBUs)^45–49^ but not the precise coordination environments of single paramagnetic metal ions. Quantum chemical methods^29,50^ were used to provide understanding of the observed paramagnetic ^1^H and ^13^C NMR shifts. This was performed for distinct scenarios involving the binding of Fe atoms to TCPP linker: high-spin (*S* = 5/2) Fe^3+^ five-coordinated ions with Cl^–^ or OH^–^ ligands at axial positions, and four-coordinated Fe^2+^ (*S* = 2) ion (cf. Figure 1b). These calculations required evaluation of electron paramagnetic resonance (EPR) spin Hamiltonian parameters: electron–nucleus hyperfine coupling tensors (***A***) for ligand nuclei of interest and electronic ***g*** tensor representing interaction of the system with an external magnetic field (Zeeman splitting). For complexes of iron, i.e., systems with multiple unpaired electrons, magnetic anisotropy effects emerge even in the absence of an external field due to zero-field splitting (ZFS ***D*** tensor), which had to be considered as well.^29^ ,^51^ −^52,53^ Accurate EPR parameters are critical for meaningful predictions of paramagnetic NMR shifts, and DFT results in this respect are typically inconsistent or even completely erratic (see discussion in section S6 in the Supporting Information).^29,54–59^ We used a state-of-the-art protocol where EPR tensors were computed with robust *ab initio* electronic-structure methods, including electron correlation:^29^ ***A*** with the coupled-cluster DLPNO-CCSD method,^29,50,60–69^ and ***g*** and ***D*** with multireference perturbation theory in the form of the CASSCF/NEVPT2 approach.^70–73^ All calculations were performed with the ORCA code,^74,75^ and total NMR shielding (**σ**) was evaluated with the integrated pNMR toolbox as follows:^51,52^

$$\sigma =\sigma_{\mathrm{orb}}-\frac{\beta_{e}S\left(S+1\right)}{g_{N}\beta_{N}3kT}g\cdot Z\cdot A$$ 1

where ***Z*** is a 3 × 3 matrix that represents |*S*λα⟩ states (eigenfunctions) and *E*_λ_ energies (eigenvalues) of the ZFS ***S**·**D**·**S*** Hamiltonian and is defined as

**Figure 3:**
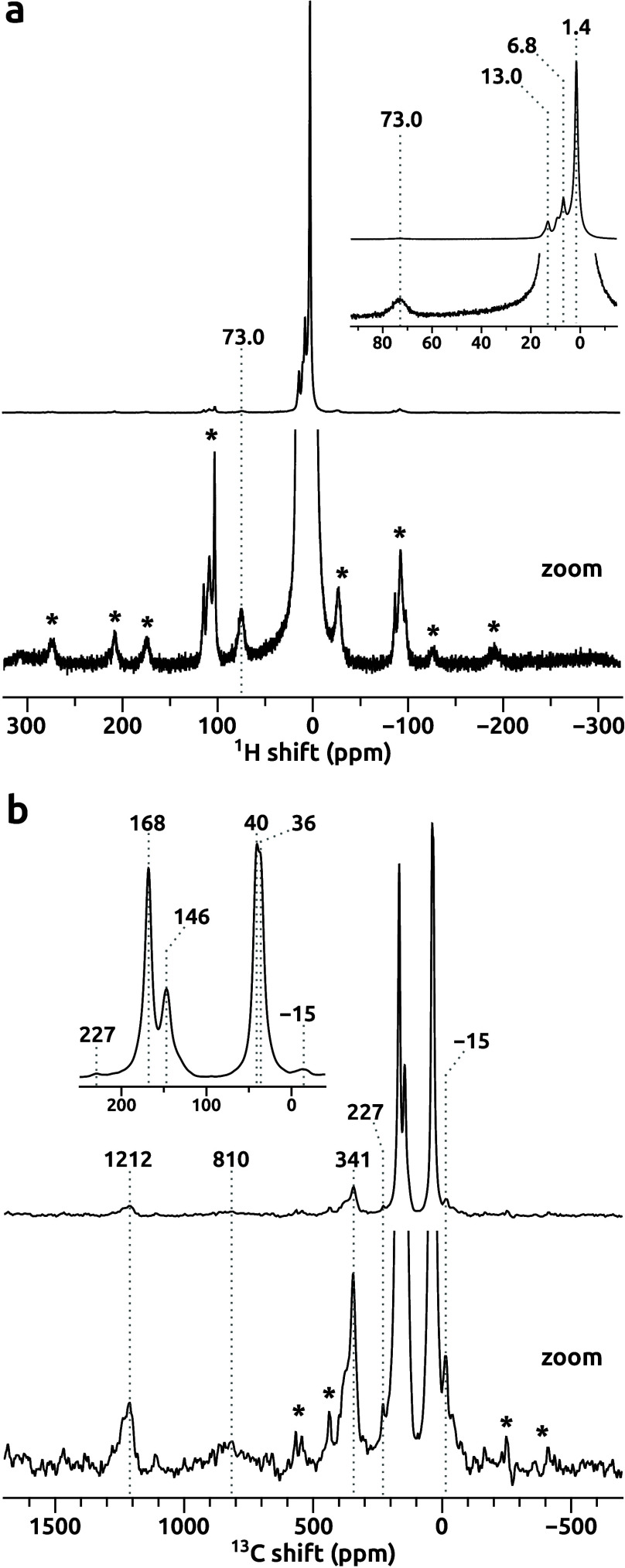
Solid-state ^1^H (a) and ^13^C (b) MAS NMR spectra of the Fe@PCN-224 MOF recorded at 14.1 T with a 60 kHz MAS rate. Spinning sidebands are marked with asterisks.

$$Z_{ij}=\frac{3}{S\left(S+1\right)}\frac{1}{Q_{0}}\underset{\lambda }{\sum }e^{-E_{\lambda }/(kT)}\cdot ,[\underset{\alpha ,\alpha^{\prime }}{\sum }\langle S\lambda \alpha |S_{i}|S\lambda \alpha^{\prime }\rangle \langle S\lambda \alpha^{\prime }|S_{j}|S\lambda \alpha \rangle +,2kT\underset{\lambda^{\prime }\neq \lambda }{\sum }\underset{\alpha ,\alpha^{\prime }}{\sum }\frac{\langle S\lambda \alpha |S_{i}|S\lambda^{\prime }\alpha^{\prime }\rangle \langle S\lambda^{\prime }\alpha^{\prime }|S_{j}|S\lambda \alpha \rangle }{E_{\lambda \prime }-E_{\lambda }}]$$ 2

where *i*, *j* = *x*, *y*, *z* and *Q*_0_ = ∑_λ,α_ *e* ^–*E*_λ_/(*kT*)^ is the partition function.

The Fe­(III)­Cl@TCPP model was investigated because Fe­(III)­Cl_3_·6H_2_O was used as a precursor during the preparation of Fe@PCN-224 MOF. All-electron DLPNO-CCSD­(T)/cc-pwCVTZ/EPR-II calculations indicated that the high-spin (*S* = 5/2) metal-ion configuration was energetically more stable by 126 kcal/mol than the low-spin (*S* = 1/2) state; hence, the latter was not considered further. All-electron DKH2-CASSCF/NEVPT2/cc-pwCVTZ-DK/cc-pVDZ-DK calculations revealed limited ***g*** anisotropy (*g*_*xx*/*yy*/*zz*_ = 2.0008/2.0008/2.0016) and a small zero-field splitting of 2.13 cm^–1^, which is consistent with the high-spin configuration exhibiting negligible spin–orbit coupling. In Table 1, the isotropic hyperfine couplings and the resulting isotropic ^1^H and ^13^C NMR shifts evaluated for the Fe­(III)­Cl@TCPP model using eqs 1 and 2 are compared with the experimental NMR shifts observed for the Fe@PCN-224 MOF (Figure 3). Based on these results, the ^1^H signals detected for the Fe@PCN-224 MOF at 73.0, 13.0, and 6.8 ppm (Figure 3a) could be unambiguously assigned to three nonequivalent aromatic proton positions in the TCPP linker: *β*, *meta*, and *ortho*. Notably, these signals were not resolved in the ^1^H MAS NMR spectrum of Fe-free PCN-224 MOF (cf. Figure 2a) owing to the small differences in the chemical shifts (**σ**_orb_). In the paramagnetic Fe@PCN-224 MOF, each of these proton positions was subjected to distinct hyperfine coupling with unpaired electrons of the Fe­(III) ion, which induced additional paramagnetic (beyond **σ**_orb_) site-specific NMR shifts that effectively increased the spectral resolution. The ^1^H signal at 1.4 ppm, which originated from the −CH_3_ groups of DMF and acetic acid molecules coordinated to the SBUs, was unaffected by the Fe ions, as these were more distant. Long-range effects are not expected because of the negligible magnetic anisotropy of high-spin Fe­(III) ions. No signal was observed at −3.2 ppm from the NH protons; hence, it could be concluded that no “empty” porphyrin rings (without Fe) were present. In the ^13^C MAS NMR spectrum of Fe@PCN-224 (Figure 3b), eight isotropic signals expected from eight nonequivalent carbon atoms were identified and assigned using the computational EPR/NMR data (Table 1). The three ^13^C NMR signals that exhibited the highest NMR shifts of 1212, 810, and 341 ppm were assigned to the *β*, *α*, and *meso* positions, respectively, consistent with the positioning of these carbon atoms in the vicinity of the paramagnetic Fe­(III) ion in the porphyrin ring. The ^13^C NMR signal of carbon *α* observed at 810 ppm was the broadest signal detected for the Fe@PCN-224 MOF. Notably, this was predicted by the *ab initio* EPR/NMR parameters in Table 1, where the standard deviations for *A*_iso_ and δ_i__so_ for ^13^C_*α*_ were the highest values. In this context, the standard deviation for *A*_iso_ can be interpreted as a propensity for the distribution of hyperfine couplings due to the known nonplanar (ruffle/saddle) distortions of substituted porphyrins,^76–79^ which result in the distribution of paramagnetic ^13^C NMR shifts observed in the spectra. The signals of ^13^C*_β_* and ^13^C_*meso*_ were significantly narrower than those of ^13^C_*α*_, and their calculated standard deviations for *A*_iso_ of 0.021 and 0.023 MHz, respectively, were substantially lower than the 0.103 MHz of ^13^C_*α*_. A considerable negative hyperfine coupling of −0.122 MHz predicted for the carbon *ipso* position resulted in the lowest calculated ^13^C shift of 10 ppm, which was assigned to the lowest observed shift of −15 ppm. Among the carbon atoms in the phenyl rings, only the *ortho* position, which is closest to the porphyrin center, experienced moderate isotropic hyperfine coupling, with the resulting calculated ^13^C NMR shift of 234.5 ppm assigned to a weakly visible signal at 227 ppm that overlapped with the more intense resonance at 168 ppm. The latter consisted of contributions from ^13^C_*para*_ and ^13^C_COO^–^_ with calculated shifts of 162.3 and 167.5 ppm, respectively, and also from carbonyl carbon atoms of DMF/acetic acid molecules present in the material (−CH_3_ groups contribute an intense signal at 36–40 ppm). The signal at 146 ppm was assigned to ^13^C_*meta*_ (calculated shift of 130.8 ppm); however, its relatively high signal intensity indicated a DMF-signal component.

**Table 1:** Isotropic Hyperfine Couplings (*A*_iso_, MHz) Obtained with the All-Electron DLPNO-CCSD/cc-pwCVTZ/EPR-II Level of Theory for the Fe­(III)­Cl@TCPP Model and Isotropic NMR Shifts (δ_i__so_, ppm) Evaluated using Eqs 1 and 2, with the Standard Deviations in Parentheses

atom	*A*_iso_ (std)	δ_iso_ (std)	δ_iso_ exp
^1^H_*β*_	0.123 (0.013)	43.8 (3.6)	73.0
^1^H_*ortho*_	–0.006 (0.001)	6.1 (0.3)	6.8
^1^H_*meta*_	0.004 (0.001)	11.5 (0.2)	13.0
^13^C_*α*_	0.542 (0.103)	759.3 (117.8)	810
^13^C_*β*_	0.692 (0.021)	923.2 (28.1)	1212
^13^C_*meso*_	0.228 (0.023)	385.9 (25.8)	341
^13^C_*ipso*_	–0.122 (0.005)	10.4 (5.5)	–15
^13^C_*ortho*_	0.082 (0.011)	234.5 (12.4)	227
^13^C_*meta*_	–0.003 (0.003)	130.8 (3.3)	146
^13^C_*para*_	0.009 (0.002)	162.3 (2.7)	168
^13^C_COO^–^_	–0.003 (0.001)	167.5 (0.9)	168

Next, we investigated the Fe­(III)­OH@TCPP and Fe­(II)@TCPP models, for which the EPR/NMR parameters were calculated using the same methodology as that for the previously discussed Fe­(III)­Cl@TCPP model. The calculated NMR shifts of all three models are plotted in Figure 4 against those observed for the Fe@PCN-224 MOF. The results for Fe­(III)­Cl@TCPP were in good agreement with the experiment, with correlation coefficients of *R* ^2^ = 0.998 and *R* ^2^ = 0.975 for the ^1^H and ^13^C NMR shifts, respectively. The results for the Fe­(III)­OH@TCPP model were in worse agreement with the experimental results, whereas those for the Fe­(II)@TCPP model deviated significantly. The extreme sensitivity of paramagnetic NMR shifts to minor changes in the coordination environments can be illustrated by the example of the ^13^C_*meso*_ signal observed at 341 ppm in the NMR spectrum of Fe@PCN-224, with the corresponding calculated shift of 385.9 ppm for the Fe­(III)­Cl@TCPP model. Notably, no signal was predicted in the 300–400 ppm “fingerprint” region upon substitution of the Cl^–^ axial ligand with OH^–^ or for the Fe­(II) ion. To examine the predictive power for revealing the local coordination geometry of paramagnetic metal ions, we compared the Fe–N and Fe– *X*_axial_ distances in the NMR-validated Fe­(III)­Cl@TCPP model to those obtained by X-ray diffraction data refinements for the Fe@PCN-224 MOF by Harris and co-workers.^16^ The geometry of the Fe­(III)­Cl@TCPP model revealed two sets of Fe–N bond distances with a difference of ∼0.002 Å: two shorter distances of 2.071 Å and two longer distances of 2.073 and 2.074 Å. This local geometry compares extremely well with the two bond pairs with Fe–N distances of 2.086 ± 0.006 and 2.088 ± 0.006 Å established from the diffraction data. Furthermore, the X-ray-derived Fe–*X*_axial_ distance of 2.227 ± 0.02 Å was in excellent agreement with the Fe–Cl distance of 2.224 Å in the Fe­(III)­Cl@TCPP model. In comparison, the geometries of the NMR-disproved models revealed significant incompatibilities with the X-ray data: an Fe–*X*_axial_(OH^–^) distance of 1.833 Å in the Fe­(III)­OH@TCPP model and Fe–N distances of 2.049–2.050 Å in the Fe­(II)­Cl@TCPP model (see section S5 in the Supporting Information).

**Figure 4:**
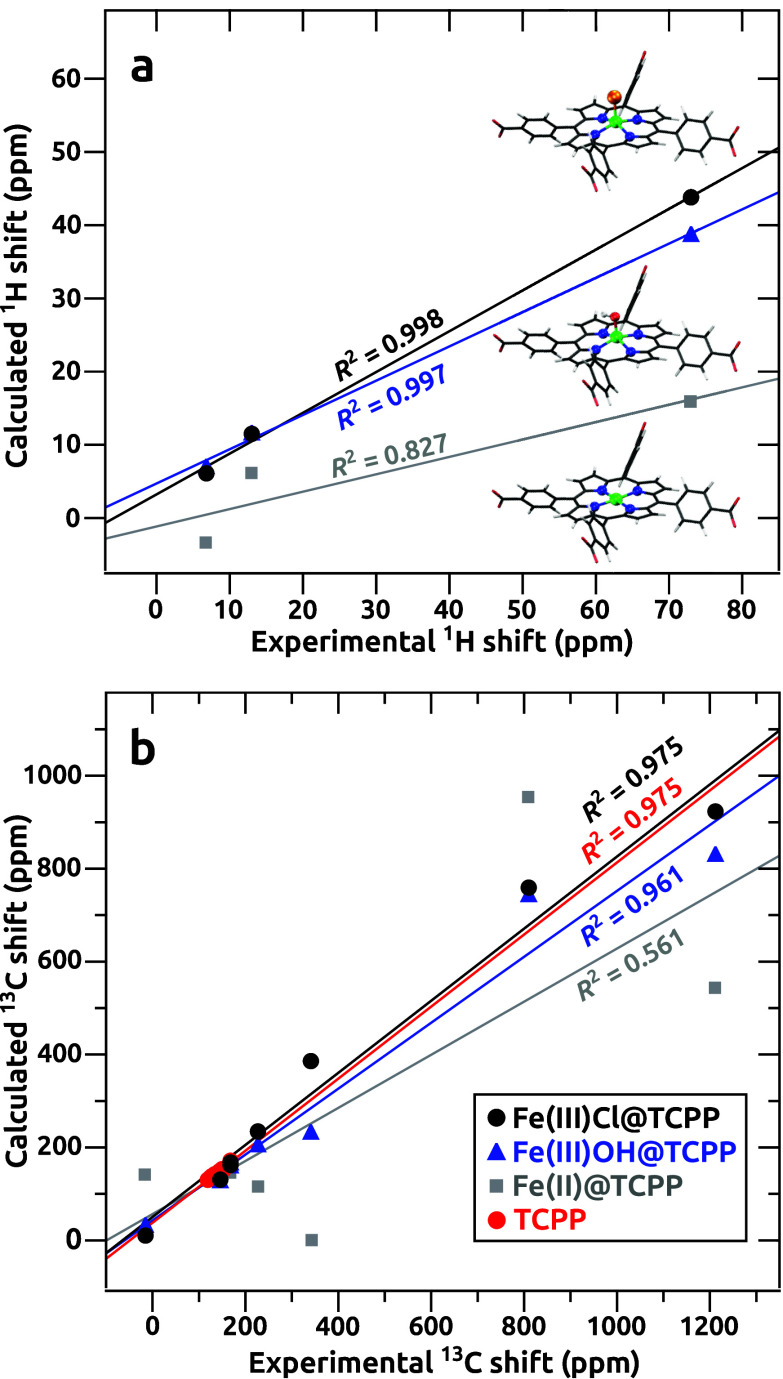
Experimentally observed ^1^H (a) and ^13^C (b) NMR shifts from the Fe@PCN-224 MOF plotted against those calculated for the indicated models. The straight lines correspond to the best-fit linear regression analysis with the resulting *R* ^2^ coefficients shown for each data set. NMR shifts from the TCPP linker were plotted against those calculated at the same level of theory, which was employed for the calculations of orbital shielding contributions to NMR shifts of paramagnetic models.

Proposed *ab initio* protocol was also verified on the Fe­(II)­(py-NMe-PiPr_2_)­Cl_2_ catalyst, for which ^1^H NMR spectra collected at 111 kHz MAS rate were recently reported.^27^ Based on our theoretical results including vibrational corrections to hyperfine couplings,^66^ we propose reassignment of two ^1^H NMR signals. After reassignment, correlation of *R* ^2^ = 0.989 between theory and experiment is obtained (see section S7 in the Supporting Information).

We have shown that the proposed approach not only discriminates distinct coordination scenarios by bridging the gap between DFT-derived models and experimental paramagnetic NMR observables but also enables the establishment of local coordination geometries of paramagnetic metal ions based solely on the NMR signatures of the observed ligand atoms. Crystalline Fe@PCN-224 MOF was used as a model example, but the same approach should be possible for use in noncrystalline SACs, molecular catalysts, and metalloproteins.

## Supplementary Material



## Notes

Materials and synthesis procedures, X-ray diffraction data, details of solid-state NMR experiments, details of quantum chemistry calculations, examples of input files for the ORCA code and the pNMR toolbox (PDF)

## References

[1] Waldron K., Rutherford J., Ford D., Robinson N. (2009). Metalloproteins and
metal sensing. Nature.

[2] Liu J., Chakraborty S., Hosseinzadeh P., Yu Y., Tian S., Petrik I., Bhagi A., Lu Y. (2014). Metalloproteins Containing
Cytochrome, Iron-Sulfur, or Copper Redox Centers. Chem. Rev..

[3] Chalkley M., Mann S., DeGrado W. (2022). De novo metalloprotein
design. Nat. Rev. Chem..

[4] Braun A., Titus C., Gee L., Baker M., Waters M., Yan J., Lee S., Nordlund D., Doriese W., O’Neil G., Schmidt D., Swetz D., Ullom J., Irwin K., Solomon E. (2025). Description of the Electronic Structure of Oxyhemoglobin
Using Fe L-Edge X-ray Absorption Spectroscopy. J. Am. Chem. Soc..

[5] Cui X., Li W., Ryabchuk P., Junge K., Beller M. (2018). Bridging Homogeneous
and Heterogeneous Catalysis by Heterogeneous Single-Metal-Site Catalysts. Nat. Catal..

[6] Wang A., Li J., Zhang T. (2018). Heterogeneous Single-Atom
Catalysis. Nat. Rev. Chem..

[7] Singh B., Gawande M., Kute A., Varma R., Fornasiero P., McNeice P., Jagadeesh R., Beller M., Zboril R. (2021). Single-Atom
(Iron-Based) Catalysts: Synthesis and Applications. Chem. Rev..

[8] Barman S., Saha P., Dey A. (2025). Second-Sphere Interaction
Allows
Selective Reduction of Nitrite to NO or Ammonia by Synthetic Iron
Porphyrins. J. Am. Chem. Soc..

[9] Armstrong J. (1999). Determination
of Chemical Valence State by X-ray Emission Analysis Using Electron
Beam Instruments: Pitfalls and Promises. Anal.
Chem..

[10] Evans D., Reed C. (2000). Reversal of H_2_O and OH^–^ Ligand Field
Strength on the Magnetochemical Series Relative to the Spectrochemical
Series. Novel 1-equiv Water Chemistry of Iron­(III) Tetraphenylporphyrin
Complexes. J. Am. Chem. Soc..

[11] Wan W., Kang L., Schnegg A., Ruediger O., Chen Z., Allen C., Liu L., Chabbra S., DeBeer S., Heumann S. (2025). Carbon-Supported Single
Fe/Co/Ni Atom Catalysts for
Water Oxidation: Unveiling the Dynamic Active Sites. Angew. Chem., Int. Ed..

[12] Morris W., Volosskiy B., Demir S., Gandara F., McGrier P. L., Furukawa H., Cascio D., Stoddart J. F., Yaghi O. M. (2012). Synthesis,
Structure, and Metalation of Two New Highly Porous Zirconium Metal–Organic
Frameworks. Inorg. Chem..

[13] Feng D., Chung W.-C., Wei Z., Gu Z.-Y., Jiang H.-L., Chen Y.-P., Darensbourg D. J., Zhou H.-C. (2013). Construction of
Ultrastable Porphyrin Zr Metal–Organic Frameworks through Linker
Elimination. J. Am. Chem. Soc..

[14] Feng D., Gu Z.-Y., Chen Y.-P., Park J., Wei Z., Sun Y., Bosch M., Yuan S., Zhou H.-C. (2014). A Highly Stable
Porphyrinic Zirconium Metal-Organic Framework with shp-a Topology. J. Am. Chem. Soc..

[15] Anderson J.
S., Gallagher A. T., Mason J. A., Harris T. D. (2014). A Five-Coordinate
Heme Dioxygen Adduct Isolated within a Metal–Organic Framework. J. Am. Chem. Soc..

[16] Zee D. Z., Harris T. D. (2020). Enhancing catalytic alkane hydroxylation by tuning
the outer coordination sphere in a heme-containing metal–organic
framework. Chem. Sci..

[17] Cichocka M. O., Liang Z., Feng D., Back S., Siahrostami S., Wang X., Samperisi L., Sun Y., Xu H., Hedin N., Zheng H., Zou X., Zhou H.-C., Huang Z. (2020). A Porphyrinic Zirconium Metal–Organic
Framework for Oxygen
Reduction Reaction: Tailoring the Spacing between Active-Sites through
Chain-Based Inorganic Building Units. J. Am.
Chem. Soc..

[18] Ren P. (2023). An Atomically
Dispersed Mn-Photocatalyst for Generating Hydrogen
Peroxide from Seawater via the Water Oxidation Reaction (WOR). J. Am. Chem. Soc..

[19] Ghosh T. (2024). A robust Fe-based heterogeneous photocatalyst for the visible-light-mediated
selective reduction of an impure CO_2_ stream. Chem. Sci..

[20] Sahoo P. K., Maiti R., Ren P., Delgado Jaén J. J., Dai X., Barcaro G., Monti A., Skorynina S., Rokicinska A., Jaworski A., Simonelli L., Kustrowski P., Rabeah J., Das S. (2025). An Atomically Dispersed
Mn Photocatalyst for Vicinal Dichlorination of Nonactivated Alkenes. J. Am. Chem. Soc..

[21] Pradhan S. (2025). An Atomically Dispersed Photocatalyst for Undirected *para*-Selective C–H Bond Functionalizations. Angew. Chem., Int. Ed..

[22] Zuo D., Pradhan S., Banerjee M., Rockstroh N., Bartling S., Rabee A., Tian X., Skorynina A., Jaworski A., Simonelli L., Rabeah J., Jiao H., Beller M., Das S. (2025). Photocatalytic Aqueous Reforming
of Methyl Formate. Adv. Mater..

[23] Waiba S. (2025). Seawater to Sustainable
Fuel: Sunlight-Driven Green Hydrogen Generation
with an Atomically Dispersed Photocatalyst. J. Am. Chem. Soc..

[24] Koppe J., Yakimov A. V., Gioffrè D., Usteri M. E., Vosegaard T., Pintacuda G., Lesage A., Pell A. J., Mitchell S., Pérez-Ramírez J., Copéret C. (2025). Coordination
environments of Pt single-atom catalysts from NMR signatures. Nature.

[25] Pell A. J., Pintacuda G., Grey C. P. (2019). Paramagnetic NMR
in Solution and
the Solid State. Prog. Nucl. Magn. Reson. Spectrosc..

[26] Koppe J., Pell A. J. (2023). Structure Determination
and Refinement of Paramagnetic
Materials by Solid-State NMR. ACS Phys. Chem.
Au.

[27] Koppe J., Sanders K. J., Robinson T. C., Lejeune A. L., Proriol D., Wegner S., Purea A., Engelke F., Clément R. J., Grey C. P., Pell A. J., Pintacuda G. (2025). Resolving
Structures of Paramagnetic Systems in Chemistry and Materials Science
by Solid-State NMR: The Revolving Power of Ultra-Fast MAS. Angew. Chem., Int. Ed..

[28] Papawassiliou W., Carvalho J. P., Paul S., Sultan A., Fardis G., Papavassiliou M., Morgan G. G., Märker K., De Paëpe G. (2026). Tracking Structural and Electron Spin Density Changes
in a Cooperative Mn^3+^ Spin Crossover Complex at Atomic
Scale via Low Temperature Solid-State NMR. Angew.
Chem., Int. Ed..

[29] Jaworski A., Hedin N. (2022). Electron correlation and vibrational effects in predictions of paramagnetic
NMR shifts. Phys. Chem. Chem. Phys..

[30] Ramsey N. F. (1950). The Internal
Diamagnetic Field Correction in Measurements of the Proton Magnetic
Moment. Phys. Rev..

[31] Ramsey N. F. (1950). Magnetic
Shielding of Nuclei in Molecules. Phys. Rev..

[32] Ramsey N. F. (1951). Dependence
of Magnetic Shielding of Nuclei upon Molecular Orientation. Phys. Rev..

[33] Ramsey N. F. (1952). Chemical
Effects in Nuclear Magnetic Resonance and in Diamagnetic Susceptibility. Phys. Rev..

[34] Wolinski K., Hinton J. F., Pulay P. (1990). Efficient Implementation of the Gauge-Independent
Atomic Orbital Method for NMR Chemical Shift Calculations. J. Am. Chem. Soc..

[35] Helgaker T., Jaszuński M., Ruud K. (1999). Ab Initio Methods for the Calculation
of NMR Shielding and Indirect Spin-Spin Coupling Constants. Chem. Rev..

[36] Vaara J. (2007). Theory and
computation of nuclear magnetic resonance parameters. Phys. Chem. Chem. Phys..

[37] Stoychev G. L., Auer A. A., Neese F. (2018). Efficient and Accurate Prediction
of Nuclear Magnetic Resonance Shielding Tensors with Double-Hybrid
Density Functional Theory. J. Chem. Theory Comput..

[38] Stoychev G. L., Auer A. A., Gauss J., Neese F. (2021). DLPNO-MP2 second derivatives
for the computation of polarizabilities and NMR shieldings. J. Chem. Phys..

[39] Dittmer A., Stoychev G. L., Maganas D., Auer A. A., Neese F. (2020). Computation
of NMR Shielding Constants for Solids Using an Embedded Cluster Approach
with DFT, Double-Hybrid DFT, and MP2. J. Chem.
Theory Comput..

[40] Szalad H., Uscategui-Linares A., García-Muelas R., Galushchinskiy A., Savateev O., Antonietti M., Albero J., García H. (2024). Natural Sunlight-Driven
Photocatalytic Overall Water Splitting with 5.5% Quantum Yield Promoted
by Porphyrin-Sensitized Zn Poly­(heptazine imide). ACS Appl. Mater. Interfaces.

[41] Koschnick C. (2021). Understanding disorder and linker deficiency in porphyrinic zirconium-based
metal–organic frameworks by resolving the Zr8O6 cluster conundrum
in PCN-221. Nat. Commun..

[42] Abraham R. J., Hawkes G. E., Hudson M. F., Smith K. M. (1975). The Nuclear
Magnetic
Resonance Spectra of Porphyrins. Part X. Carbon 13 Nuclear Magnetic
Resonance Spectra of Some meso-Tetra-arylporphyrins and their Metal
Chelates. J. Chem. Soc., Perkin Trans..

[43] Hwang T. L., van Zijl P. C. M., Garwood M. (1998). Fast Broadband
Inversion by Adiabatic
Pulses. J. Magn. Reson..

[44] Kervern G., Pintacuda G., Emsley L. (2007). Fast Adiabatic Pulses for Solid-State
NMR of Paramagnetic Systems. Chem. Phys. Lett..

[45] Wittmann T., Mondal A., Tschense C. B. L., Wittmann J. J., Klimm O., Siegel R., Corzilius B., Weber B., Kaupp M., Senker J. (2018). Probing Interactions
of N-Donor Molecules with Open
Metal Sites within Paramagnetic Cr-MIL-101: A Solid-State NMR Spectroscopic
and Density Functional Theory Study. J. Am.
Chem. Soc..

[46] Blahut J., Lejeune A. L., Ehrling S., Senkovska I., Kaskel S., Wisser F. M., Pintacuda G. (2021). Monitoring
Dynamics, Structure, and Magnetism of Switchable Metal– Organic
Frameworks via ^1^H-Detected MAS NMR. Angew. Chem., Int. Ed..

[47] Klug C. A., Swift M. W., Miller J. B., Lyons J. L., Albert A., Laskoski M., Hangarter C. M. (2022). High Resolution Solid State NMR in
Paramagnetic Metal-Organic Frameworks. Solid
State Nucl. Magn. Reson..

[48] Carretero-Cerdan A., Carrasco S., Sanz-Marco A., Jaworski A., Martín-Matute B. (2023). One-Step Microwave-Assisted
Synthesis of Amino-Functionalized Chromium­(III) Terephthalate MIL-101-NH_2_. Materials Today Chemistry.

[49] Phan H., Gueret R., Martínez-Pardo P., Valiente A., Jaworski A., Slabon A., Martín-Matute B. (2025). Synthesis
of Benzoic Acids from Electrochemically Reduced CO_2_ Using
Heterogeneous Catalysts. ChemSusChem.

[50] Neese F., Atanasov M., Bistoni G., Maganas D., Ye S. (2019). Chemistry
and Quantum Mechanics in 2019: Give Us Insight and Numbers. J. Am. Chem. Soc..

[51] Soncini A., Van den Heuvel W. (2013). Communication: Paramagnetic NMR chemical shift in a
spin state subject to zero-field splitting. J. Chem. Phys..

[52] Van
den Heuvel W., Soncini A. (2013). NMR chemical shift as analytical
derivative of the Helmholtz free energy. J.
Chem. Phys..

[53] Vaara J., Rouf S. A., Mareš J. (2015). Magnetic Couplings
in the Chemical
Shift of Paramagnetic NMR. J. Chem. Theory Comput..

[54] Munzarová M., Kaupp M. (1999). A Critical Validation
of Density Functional and Coupled-Cluster Approaches
for theCalculation of EPR Hyperfine Coupling Constants in Transition
Metal Complexes. J. Phys. Chem. A.

[55] Neese F. (2003). Metal and
ligand hyperfine couplings in transition metal complexes: The effect
of spin–orbit coupling as studied by coupled perturbed Kohn–Sham
theory. J. Chem. Phys..

[56] Kaupp M., Arbuznikov A. V., Heßelmann A., Görling A. (2010). Hyperfine
coupling constants of the nitrogen and phosphorus atoms: A challenge
for exact-exchange density-functional and post-Hartree–Fock
methods. J. Chem. Phys..

[57] Schattenberg C. J., Maier T. M., Kaupp M. (2018). Lessons from the Spin-Polarization/Spin-Contamination
Dilemma of Transition-Metal Hyperfine Couplings for the Construction
of Exchange-Correlation Functionals. J. Chem.
Theory Comput..

[58] Bertarello A., Benda L., Sanders K. J., Pell A. J., Knight M. J., Pelmenschikov V., Gonnelli L., Felli I. C., Kaupp M., Emsley L., Pierattelli R., Pintacuda G. (2020). Picometer
Resolution Structure of the Coordination Sphere in the Metal-Binding
Site in a Metalloprotein by NMR. J. Am. Chem.
Soc..

[59] Pyykkönen A., Wodyński A., Kaupp M., Vaara J. (2025). First-principles paramagnetic
NMR of a challenging Fe­(V) bis­(imido) complex: a case for novel density
functionals beyond the zero-sum game. Phys.
Chem. Chem. Phys..

[60] Perera S. A., Watts J. D., Bartlett R. J. (1994). A theoretical study of hyperfine
coupling constants. J. Chem. Phys..

[61] Bartlett R. J., Musiał M. (2007). Coupled-Cluster
Theory in Quantum Chemistry. Rev. Mod. Phys..

[62] Saitow M., Becker U., Riplinger C., Valeev E. F., Neese F. (2017). A new near-linear
scaling, efficient and accurate, open-shell domain-based local pair
natural orbital coupled cluster singles and doubles theory. J. Chem. Phys..

[63] Saitow M., Neese F. (2018). Accurate Spin-Densities
Based on the Domain-Based Local Pair-Natural
Orbital Coupled-Cluster Theory. J. Chem. Phys..

[64] Guo Y., Riplinger C., Liakos D. G., Becker U., Saitow M., Neese F. (2020). Linear scaling
perturbative triples correction approximations for
open-shell domain-based local pair natural orbital coupled cluster
singles and doubles theory [DLPNO-CCSD­(T0/T1)]. J. Chem. Phys..

[65] Witwicki M., Walencik P. K., Jezierska J. (2020). How Accurate is Density Functional
Theory in Predicting Spin Density? An Insight from the Prediction
of Hyperfine Coupling Constants. J. Mol. Model..

[66] Auer A. A., Tran V. A., Sharma B., Stoychev G. L., Marx D., Neese F. (2020). A case study of density
functional theory and domain-based local
pair natural orbital coupled cluster for vibrational effects on EPR
hyperfine coupling constants: vibrational perturbation theory versus
ab initio molecular dynamics. Mol. Phys..

[67] Ma Z., Lu C., Chen J., Rokicińska A., Kuśtrowski R., Coridan P., Dronskowski R., Slabon A., Jaworski A. (2021). CeTiO_2_N oxynitride perovskite:
paramagnetic ^14^N MAS NMR
without paramagnetic shifts. Z. Naturforsch.,
B.

[68] Jaworski A., Piatek J., Mereacre L., Braun C., Slabon A. (2021). 14N, 13C,
and 119Sn solid-state NMR characterization of tin (II) carbodiimide
Sn (NCN). Z. Naturforsch., B.

[69] Sharma B., Tran V. A., Pongratz T., Galazzo L., Zhurko I., Bordignon E., Kast S. M., Neese F., Marx D. (2021). A Joint Venture
of Ab Initio Molecular Dynamics, Coupled Cluster Electronic Structure
Methods, and Liquid-State Theory to Compute Accurate Isotropic Hyperfine
Constants of Nitroxide Probes in Water. J. Chem.
Theory Comput..

[70] Ye S., Neese F. (2012). How Do Heavier Halide Ligands Affect the Signs and
Magnitudes ofthe
Zero-Field Splittings in Halogenonickel­(II) ScorpionateComplexes?
A Theoretical Investigation Coupled to Ligand-Field Analysis. J. Chem. Theory Comput..

[71] Ganyushin D., Neese F. (2013). A Fully Variational Spin-Orbit Coupled Complete Active Space Self-Consistent
Field Approach: Application to Electron Paramagnetic Resonance g-Tensors. J. Chem. Phys..

[72] Jiang S., Maganas D., Levesanos N., Ferentinos E., Haas S., Thirunavukkuarasu K., Krzystek J., Dressel M., Bogani L., Neese F., Kyritsis P. (2015). Direct Observation
of Very Large Zero-Field Splitting in a Tetrahedral Ni^II^Se_4_ Coordination Complex. J. Am.
Chem. Soc..

[73] Singh S. K., Atanasov M., Neese F. (2018). Challenges in Multireference Perturbation
Theory for the Calculations of the g-Tensor of First-Row Transition-Metal
Complexes. J. Chem. Theory Comput..

[74] Neese F. (2012). The ORCA Program
System. Wiley Interdiscip. Rev. Comput. Mol.
Sci..

[75] Neese F., Wennmohs F., Becker U., Riplinger C. (2020). The ORCA Quantum
Chemistry Program Package. J. Chem. Phys..

[76] Medforth C. J., Senge M. O., Smith K. M., Sparks L. D., Shelnutt J. A. (1992). Nonplanar
Distortion Modes for Highly Substituted Porphyrins. J. Am. Chem. Soc..

[77] Sparks L. D., Medforth C. J., Park M. S., Chamberlain J. R., Ondrias M. R., Senge M. O., Smith K. M., Shelnutt J. A. (1993). Metal Dependence
of the Nonplanar Distortion of Octaalkyltetraphenylporphyrins. J. Am. Chem. Soc..

[78] Ryeng H., Ghosh A. (2002). Do Nonplanar Distortions of Porphyrins Bring about Strongly Red-Shifted
Electronic Spectra? Controversy, Consensus, New Developments, and
Relevance to Chelatases. J. Am. Chem. Soc..

[79] Nakamura M. (2006). Electronic
Structures of Highly Deformed Iron­(III) Porphyrin Complexes. Coord. Chem. Rev..

