# Supplementary material for: Acquiring Focus on Paramagnetic Single-Atom Sites with Fast Magic-Angle Spinning NMR
Source: J Am Chem Soc. 2026 Feb 14;148(7):6772–8. doi: 10.1021/jacs.5c20153 (PMC12951433; doi:10.1021/jacs.5c20153)
Supplement: Supplementary file 1 [file ja5c20153_si_001.pdf]

SUPPORTING INFORMATION

Acquiring Focus on Paramagnetic Single-Atom Sites with Fast Magic-Angle Spinning NMR

Ioannis Mylonas-Margaritis[†,‡], Zhehao Huang[†,‡], Niklas Hedin[†], and Aleksander Jaworski[*,†]

[†]Department of Chemistry, Stockholm University, SE-106 91 Stockholm, Sweden
[‡]Wallenberg Initiative Materials Science for Sustainability, Department of Chemistry, Stockholm University, SE-106 91 Stockholm, Sweden

[*]Corresponding author: Aleksander Jaworski
Email: *aleksander.jaworski@su.se*

Content:

S1. Materials and synthesis
S2. Solid-state NMR
S3. Theoretical methods
S4. Not straightforward prediction of ^{13}C NMR shifts for TCPP
S5. Models of the Fe@PCN-224 MOF linker
S6. Prediction of hyperfine coupling constants: the challenge
S7. Calculations for the Fe(II)(py-NMe-PiPr$_2$)Cl$_2$ catalyst
S8. Examples of input commands for the ORCA code
S9. Supplementary data: atomic coordinates, hyperfine tensors, vibrational frequencies
References

S1. Materials and synthesis

Zirconium(IV) oxide chloride octahydrate (CAS number 13520-92-8) was bought in February 2022 from Alfa Aesar. TCPP [Tetrakis(4-carboxylphenyl)porphyrin] (CAS number is 14609-54-2) was bought from TCI Chemicals in September 2024. Iron(III) chloride hexahydrate 99+% (CAS number 10025-77-1) was bought from Acros Organics in February 2022. *N*,*N*-Dimethylformamide (DMF) was bought from VWR in August 2024 (20l plastic tank number: 3731205025799157195, batch number: 24G314016, VWR number VWRC23470.442). Acetic acid (glacial, CAS number 64-19-7, ACS, Reag. Ph. Eur.) was bought in January 2023 from VWR.

Compound 1 (PCN-224): 10.0 ml of DMF and 2.5 ml of acetic acid were placed in a glass vial. 0.0132 g of Zirconium(IV) oxide chloride octahydrate was added into the vial and sonicated. After 3 min 0.0289 g of TCPP was added into the cloudy solution. The vial was placed into an oven preheated to 150 degree Celsius and kept there for 3 days. The solution retained a purple color. The precipitated compound was washed two times with 10 ml of DMF.

Compound 2 (Fe@PCN-224): 50 ml of DMF was placed into a round bottom flask and 0.0197 g of the isolated Compound 1 and 0.0251 g of iron (III) chloride were added. Reflux was performed for 12 h. The reaction mixture retained a pale purple color and the solid product was filtrated by vacuum filtration and washed two times with 10 ml of DMF.

Powder X-ray diffraction (PXRD) data of both compounds were collected on the PANalytical PRO MPD diffractometer in the range from 3 to 40 degrees using a mask (width 20) and a knife to protect the detector. The resulting PXRD patterns are shown in Figure S1 against the simulated patterns derived from the crystallographic data of Zee and Harris,[1] which were deposited in the CCDC database with the respective numbers 1992907 and 1992908. The PXRD pattern of PCN224 was compared with all proposed crystal structures and in overall is matching data of Harris[1] and Koschnick and coworkers, [2] however, we note that the peak at 5.633° (hkl 211) does not exist in any of the reported experimental PXRD data.

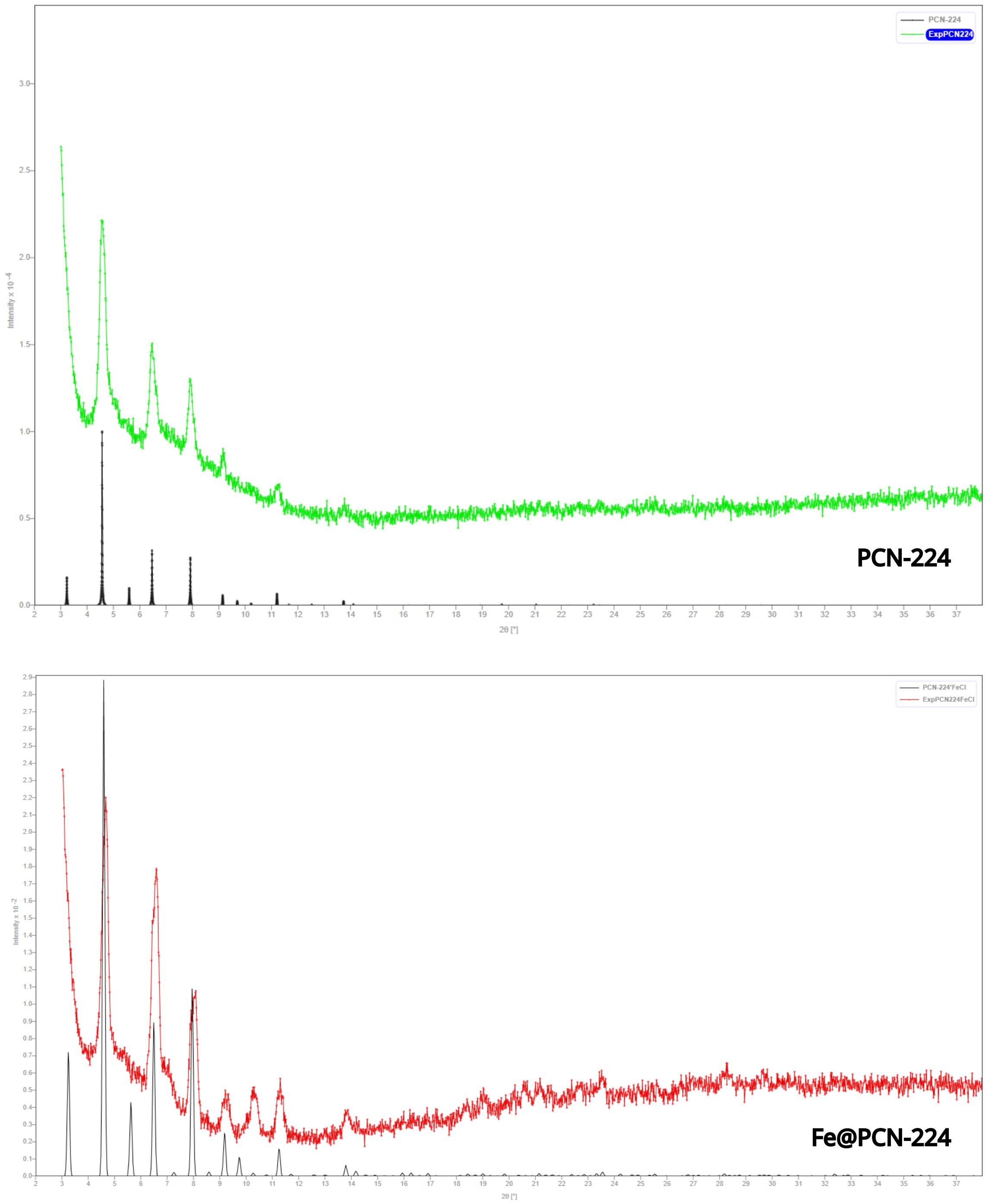

Figure S1: Comparison of the experimental PXRD patterns of PCN-224 (green trace, upper panel) and Fe@PCN-224 (red trace, lower panel) to respective calculated patterns based on the data retrieved from CCDC database; structures number 1992907 and 1992908 of ref;[1] generated using CrystalDiffract®, CrystalMaker Software Ltd, Oxford, England (www.crystalmaker.com).

S2. Solid-state NMR

Magic-angle spinning (MAS) NMR experiments were performed at a magnetic field of 14.1 T (Larmor frequencies of 600.12 and 150.92 for ^{1}H and ^{13}C, respectively) on a Bruker wide-bore Avance-III spectrometer. All spectra were acquired using a Bruker 1.3 mm double-resonance probehead and MAS rate $\nu_r = 60.00$ kHz. Acquisitions of ^{1}H MAS spectra involved a use of a rotor-synchronized, double-adiabatic spin-echo sequence with a 90 degree excitation pulse of 1.25 μs followed by a pair of 50.0 μs tanh/tan short high-power adiabatic pulses (SHAPs) with a 5 MHz frequency sweep.[3, 4] All pulses operated at the nutation frequency of 200 kHz. 128 signal transients were acquired using a relaxation delay 5 s. ^{13}C MAS spectrum of paramagnetic Fe@PCN-224 MOF employed the same rotor-synchronized, double-adiabatic spin-echo sequence but pulses operated at the nutation frequency of 100 kHz and 917504 scans were acquired using a relaxation delay 0.5 s.

SHAPs offer excellent broadband population inversion performance, however, their considerable length (typically tens of μs) has potential drawbacks in case of paramagnetic systems. This relates to the presence of the NMR "blind sphere" in close vicinity to the paramagnetic metal ion, where effects of signal broadening due to paramagnetic relaxation enhancement (PRE) and fast transverse relaxation are the strongest. In extreme cases, signals can be broadened beyond detection. In this work we used robust SHAPs with duration of 3 rotor periods, which for 1.3 mm rotor with MAS rate $\nu_r = 60.00$ kHz translates to 50.0 μs. With this setup complete set of ^{1}H and ^{13}C resonances from Fe@PCN-224 MOF could be successfully detected and no significant paramagnetic broadening was observed. However, this is not guaranteed to be the case in other systems, since PRE effects depend on electronic properties of the particular metal ion and the chemistry of the system. For the most challenging cases, shorter SHAPs, spanning 2, or even 1 rotor period can be generated and used; or smaller rotors capable of MAS rates above 100 kHz can be employed to considerably shorten duration of the irradiation schemes (due to shorter duration of each rotor period).[5] On the other hand, compared to 1.3 mm rotor, direct detection of ^{13}C at natural abundance would be extremely challenging with samller rotors due to substantially reduced sample volume (roughly 5$\times$ smaller for the 0.7mm rotor, and 25$\times$ smaller for the 0.4 mm rotor when compared to 1.3 mm rotor used herein). Further challenges are expected for carbon-based catalysts with low metal loadings, where ^{13}C isotopic enrichment might be needed in case when more NMR receptive nuclei are not present in the studied coordination environments.

Cross-polarization ^{1}H-^{13}C CPMAS NMR spectra of TCPP linker and PCN-224 MOF involved Hartmann-Hahn radiofrequency fields ($\nu_{\rm H} = 40$, $\nu_{\rm C} = 20$ kHz) matched on double-quantum (DQ; $\nu_{\rm H} + \nu_{\rm C} = \nu_r$) condition and applied for a contact interval of 1.5 ms. SPINAL-64 proton decoupling of $\nu_{\rm H} = 150$ kHz was employed. 32768 signal transients were collected using a relaxation delay of 2 s. Variable temperature (VT) BCU Xtreme unit was used to stabilize sample temperature inside the rotor at 320 K during experiments, which was verified by measuring ^{207}Pb NMR shift of solid lead nitrate ($Pb(NO_3)_2$) for the same conditions of MAS rate and VT gas temperature setting and flow. ^{1}H and ^{13}C NMR shifts are reported with respect to tetramethylsilane (TMS, $Si(CH_3)_4$).

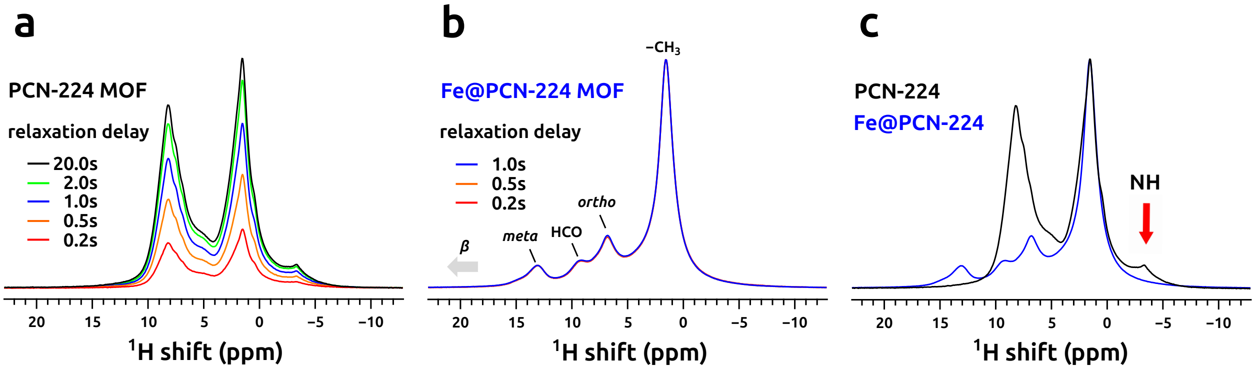

Figure S2: ^{1}H T_1 relaxation tests for the as-synthesized PCN-224 MOF (a) and for the Fe@PCN-224 MOF sample (b); comparison of fully relaxed ^{1}H MAS NMR spectra of PCN-224 and Fe@PCN-224 MOF samples (c). The latter exhibits significantly faster relaxation with full recovery just after 0.2 s (compared to 20 s for the MOF sample without Fe ions). Three ^{1}H signals from the TCPP linker (β, *meta*, and *ortho*) are not well resolved in the 6−10 ppm region in panel a, and signal from NH protons of porphyrin core is visible at −3 ppm. After complete Fe complexation into the porphyrin core (see no detectable signal of NH protons after reaction; marked with red arrow in panel c) $^1H_\beta$, $^1H_{meta}$, and $^1H_{ortho}$ signals from the linker are clearly resolved at 73 (not visible with this scale, see Fig. 3 in the main text), 13, and 7 ppm, respectively, due to paramagnetic NMR shifts interactions within the porphyrin linker, whereas the shift of $-CH_3$ groups at around 2 ppm from the residual DMF/acetic acid solvent molecules is unchanged upon incorporation of Fe ions. Presence of $-CH_3$ groups from residual solvent molecules is evidenced by ^{13}C signals in the 25−35 ppm region (Fig. 2 in the main text). Characteristic signal of formyl protons (HCO) from DMF is detected at 8 ppm.

S3. Theoretical methods

All calculations were performed with the ORCA code[6–8] version 5.0.1,[9] and employed a tight self-consistent field (SCF) convergence tolerance of 1×10^{-8} E_h (TightSCF). Evaluation of Coulomb and exchange integrals was accelerated with the RIJCOSX approximation[10] employing either def2/J[11] or automatically generated (AutoAux)[12] Coulomb-fitting basis sets (for relativistic or augmented orbital basis sets). Default integration grid settings were used. All calculations were performed on a cluster nodes equipped with the two Intel® Xeon® Gold 6242R CPUs (40 cores; 3.10 GHz) and 256 GB of RAM each.

Geometry optimizations of the models were converged to tight thresholds (TightOpt) with tolerances for energy change and root mean squared gradient of 1×10^{-6} E_h and 3×10^{-5} E_h/bohr respectively. Optimizations employed the PBE0[13] DFT approximation together with a density dependent atom-pairwise dispersion correction (D4)[14] and the def2-TZVP basis set[15]. Open-shell systems were optimized assuming high-spin electronic configuration. All models were confirmed to correspond to global energy minima at the corresponding potential energy surfaces by numerical Hessian calculations.

Orbital shielding tensors $\boldsymbol{\sigma}$ were calculated with the GIAO approach[16] at the PBE0 level using pcSseg-n (n= 1,2) basis sets specifically developed for NMR shielding calculations.[17] Additional calculations with the double hybrid DLPNO-DSD-PBEP86[18, 19] DFT approximation and the DLPNO-MP2[20–22] method involved relaxed MP2 density and all electrons (NoFrozenCore). The isotropic shielding σ and subsequently the isotropic shift δ are obtained according to:

$$\sigma = \frac{\sigma_{\mathrm{xx}} + \sigma_{\mathrm{yy}} + \sigma_{\mathrm{zz}}}{3}; \quad \delta = \sigma_{\mathrm{ref}} - \sigma + \delta_{\mathrm{ref}} \tag{S1}$$

where σ_{ref} and δ_{ref} are the shielding and chemical shifts of reference compound. The CH_4 molecule was used as NMR shift reference since ^{1}H and ^{13}C chemical shifts measured in the gas phase are available for this molecule (^{1}H $\delta_{\mathrm{ref}} = 2.17$ ppm; ^{13}C $\delta_{\mathrm{ref}} = -8.65$ ppm).[23–25]

All-electron (NoFrozenCore) DLPNO-CCSD calculations of the hyperfine tensors $\boldsymbol{A}$ employed unrelaxed coupled cluster density, quasi-restricted orbitals (QROs), and default truncation thresholds for the PNO space (NormalPNO). Open-shell CCSD and Λ/Z-vector-iterations were converged to within of 1×10^{-6}

(residual norm), and the T1 diagnostics were $\leqslant$0.016 for both CCSD and Λ/Z-vector iterations. The cc-pwCVTZ basis set[26, 27] was used for the Fe and Cl atoms, and EPR-II basis set[28–31] for O, N, C, and H. The cc-pwCVTZ/C and cc-pwCVDZ/C auxiliary basis set were used for Fe,Cl and O, N, C, H atoms, respectively.[32, 33] All-electron DLPNO-CCSD(T) calculations of spin-states energetics employed the same basis sets and PNO truncation setting as above. DLPNO-CCSD(T) calculations for energetics of TCPP models without metal ions involved the cc-pVTZ orbital basis sets[34–36] on all atoms and the corresponding cc-pVTZ/C auxiliary basis sets.[32]

The electronic $\boldsymbol{g}$ tensor and zero-field splitting $\boldsymbol{D}$ tensor were calculated with all-electron state-averaged, complete active space self-consistent field (CASSCF) wave function distributing 5 electrons over 5 $3d$-orbitals of the Fe^{3+} ion [CAS(5,5)] and followed by the N-electron valence second-order perturbation theory (NEVPT2); 1 sextet, 20 quartet, and 30 doublet roots were considered. For the Fe^{2+} ion [CAS(6,5)] 5 quintet, 20 triplet, and 30 singlet roots were evaluated. Both scalar-relativistic (via the second-order Douglas-Kroll-Hess (DKH2) approach, picture changed operators, and finite nucleus model)[37–40] and spin-orbit effects were included.[41, 42] For the DKH2-CASSCF/NEVPT2 calculations the cc-pwCVTZ-DK basis set[43] was used for Fe and cc-pVDZ-DK basis set[44] for the remaining atoms in the model. Auxiliary basis sets generated with the AutoAux were employed.

NMR shielding for the system with spin quantum number S was parametrized with EPR spin Hamiltonian parameters and evaluated with pNMR tool as:[45, 46]

$$\boldsymbol{\sigma} = \boldsymbol{\sigma}_{\mathrm{orb}} - \frac{\beta_e S(S+1)}{g_{\mathrm{N}}\beta_{\mathrm{N}} 3kT}\, \boldsymbol{g}\cdot \boldsymbol{Z}\cdot \boldsymbol{A} \tag{S2}$$

where β_e, g_{N}, β_{N}, k, and T are the Bohr magneton, nuclear g-factor (depending on the type of nucleus), nuclear magneton, Boltzmann constant, and absolute temperature, respectively. Note that $\boldsymbol{g}$ represents the Zeeman splitting in the external magnetic field and is a property of the system (metal ion + ligand atoms) as a whole, independent of the nuclear moments, whereas $\boldsymbol{A}$ represents the hyperfine interaction between the (unpaired) electrons and the nucleus of interest, therefore each nucleus will have its own hyperfine tensor $\boldsymbol{A}$ and in consequence shielding tensor $\boldsymbol{\sigma}$. Orbital shielding tensor $\boldsymbol{\sigma}_{\mathrm{orb}}$ corresponds to the Ramsey shielding theory,[47–54] which is the only contribution to NMR "chemical shift" for diamagnetic closed-shell systems, without unpaired electrons. $\boldsymbol{Z}$ is a 3×3 matrix that represents $|S\lambda\alpha\rangle$ states (eigenfunctions) and E_λ energies (eigenvalues) of the ZFS $\boldsymbol{S}\cdot \boldsymbol{D}\cdot \boldsymbol{S}$ Hamiltonian, and is defined as:

$$Z_{ij} = \frac{3}{S(S+1)}\frac{1}{Q_0}\sum_{\lambda} e^{-E_\lambda/kT}\left[\sum_{\alpha,\alpha'}\langle S\lambda\alpha|S_i|S\lambda\alpha'\rangle\langle S\lambda\alpha'|S_j|S\lambda\alpha\rangle + 2kT\sum_{\lambda'\neq\lambda}\sum_{\alpha,\alpha'}\frac{\langle S\lambda\alpha|S_i|S\lambda'\alpha'\rangle\langle S\lambda'\alpha'|S_j|S\lambda\alpha\rangle}{E_{\lambda'}-E_\lambda}\right], \tag{S3}$$

where $i, j = x, y, z$ and $Q_0 = \sum_{\lambda,\alpha} e^{-E_\lambda/kT}$ is the partition function. Induced paramagnetic NMR shielding was evaluated assuming temperature of 320 K for the Fe@PCN-224 MOF and 293 K for the Fe(py-NMe-$PiPr_2$)Cl_2 catalyst, in accordance to experimental MAS NMR conditions. EPR/NMR properties of atoms at equivalent chemical positions were averaged.

For calculations of vibrational corrections to $\boldsymbol{A}$ the second-order vibrational perturbation theory (VPT2) was employed.[55] Hessian calculations for the anharmonic VPT2 force fields were carried out at the PBE-D4/def2-SVP level starting from the respective tightly converged geometry. For the numerical calculation of Hessian and property derivatives stepsize of 0.05 was used for both anharmonic and property displacements. PBE0/def2-TZVP(Fe,Cl,P)/EPR-II(N,C,H) level of theory was employed for property calculations. 222 Hessian calculations and 222 property calculations at displaced geometries were needed for the Fe(II)(py-NMe-$PiPr_2$)Cl_2 molecule.

S4. Not straightforward prediction of ^{13}C NMR shifts for TCPP

We noted that standard GGA (PBE) and hybrid-GGA (PBE0) DFT approximations have problems to provide robust assignments of ^{13}C NMR shifts for the TCPP linker, see respective plots in Figure S3. Electron correlation corrections in perturbatively-corrected DLPNO-DSD-PBEP86 approximation clearly improve DFT performance in this respect, which is confirmed in the last plot, where shifts computed with an *ab initio* method, the second order Møller–Plesset perturbation theory (DLPNO-MP2) are shown. Also note overestimated NMR shifts for β carbon signal by PBE and PBE0 methods in Table S1. According to liquid-state NMR spectra these signals should be below 130 ppm,[56] which is confirmed by the results at DLPNO-DSD-PBEP86 and DLPNO-MP2 levels of theory. In particular, we note high reliability of the DLPNO-DSD-PBEP86 method for predictions for difficult carbon positions C_β, C_{meso} in the porphyrin core, where electron correlation effects are expected to be substantial, and predictions by GGA and hybrid-GGA deviate considerably from experiment, especially GGA-PBE prediciton with error of almost 11 ppm for C_{meso}.[57] Problems of the GGA PBE DFT approximation for description of NMR shifts for the TCPP linker may have serious drawbacks in periodic DFT predictions on MOFs, where additional errors from plane-wave basis sets for NMR predictions might come into play as well.

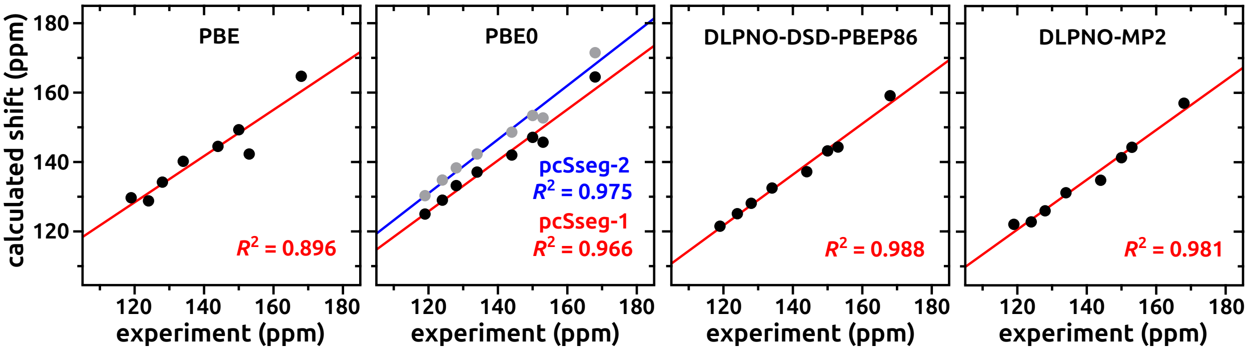

Figure S3: Calculated ^{13}C NMR shifts TCPP with psSseg-1 basis set (for PBE0 data with larger pcSseg-2 basis set shown in gray).

Table S1: Isotropic ^{13}C NMR shifts (δ, ppm) calculated for the TCPP model with the psSseg-1 basis set (for PBE0 data with the larger pcSseg-2 basis set shown in parenthesis).

Atom	PBE	PBE0	DLPNO-DSD-PBEP86	DLPNO-MP2
C_α	149.3	147.1(153.4)	143.2	141.3
C_β	134.2	133.2(138.3)	128.1	126.0
C_{meso}	129.7	125.0(130.3)	121.5	122.1
C_{ipso}	144.5	142.0(148.6)	137.2	134.8
C_{ortho}	140.2	137.1(142.3)	132.5	131.2
C_{meta}	128.8	129.0(134.8)	125.1	122.8
C_{para}	142.3	145.7(152.7)	144.3	144.3
C_{COO^-}	164.7	164.5(171.5)	159.1	157.0
Correlation with experiment (R^2)	0.896	0.966(0.975)	0.988	0.981

S5. Models of the Fe@PCN-224 MOF linker

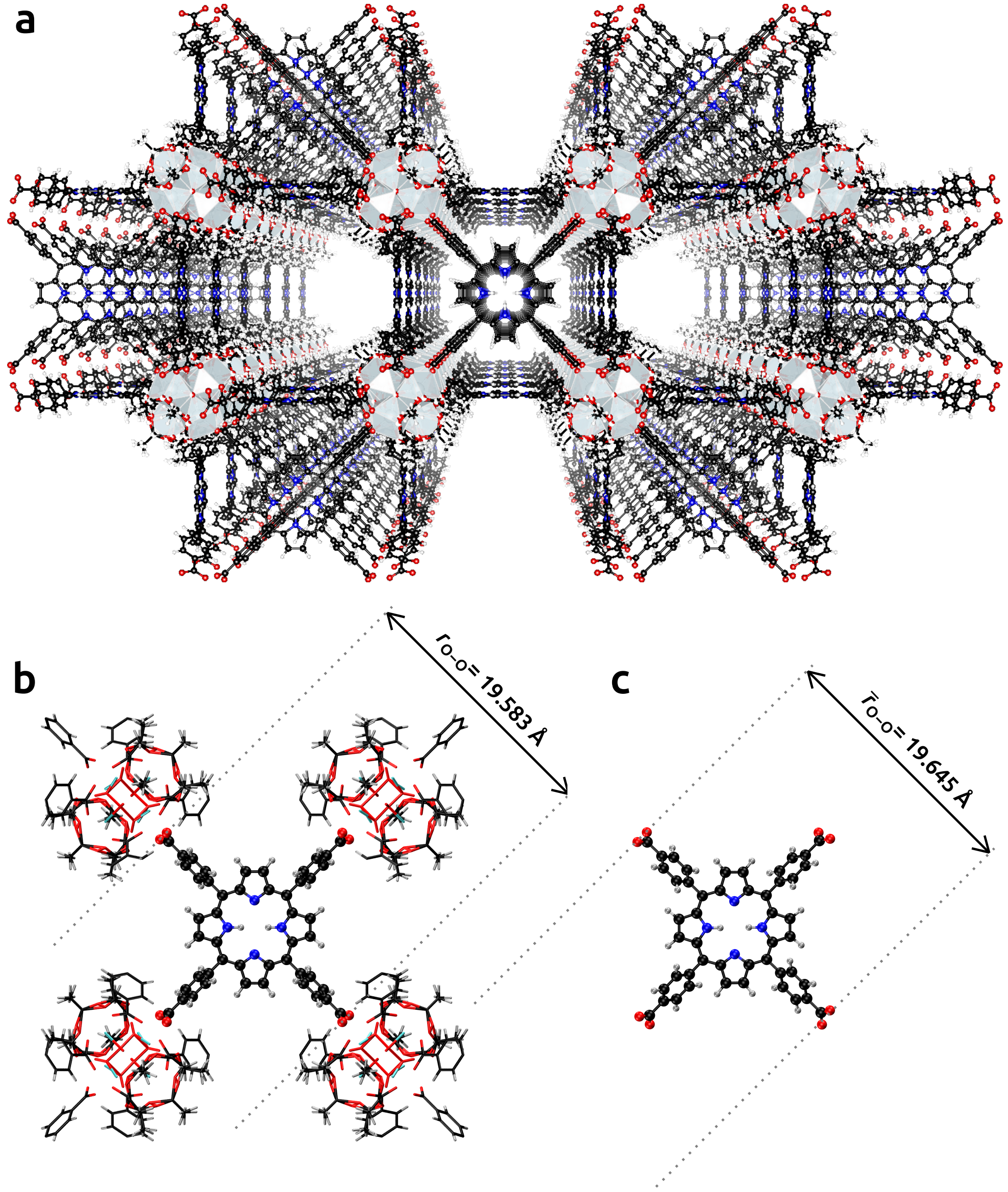

Figure S4: Panel a: crystal structure of PCN-224 MOF generated from crystallographic data by Harris[1] (note double occupations of proton positions). Panel b: model of the TCPP linker ($[C_{48}H_{26}N_4O_8]^{4-}$; atoms rendered with spheres) with atomic coordinates of O, N, and C corresponding to those in the crystal structure. Two (doubly occupied) NH protons were removed, and atomic coordinates of all protons in the model were optimized at the PBE0-D4/def2-TZVP level of theory while keeping all remaining (O, N, C) coordinates frozen. This is because C−H and N−H bond lengths of $r_{\mathrm{N-H}} = 0.950$ and $r_{\mathrm{N-H}} = 0.880$ in the crystallographic information file (.cif) were unrealistically short and caused pathological convergence issues in electronic structure calculations. This model is called X-ray + opt. H geometry. Panel c: model of the TCPP linker after geometry-optimization of all atomic coordinates at the PBE0-D4/def2-TZVP level of theory and called fully optimized geometry. We note only tiny change in O−O distances from 19.583 Å in the crystal structure of PCN-224 MOF to average value of 19.645 Å (change of 0.062 Å) after full geometry-optimization of the TCPP molecule.

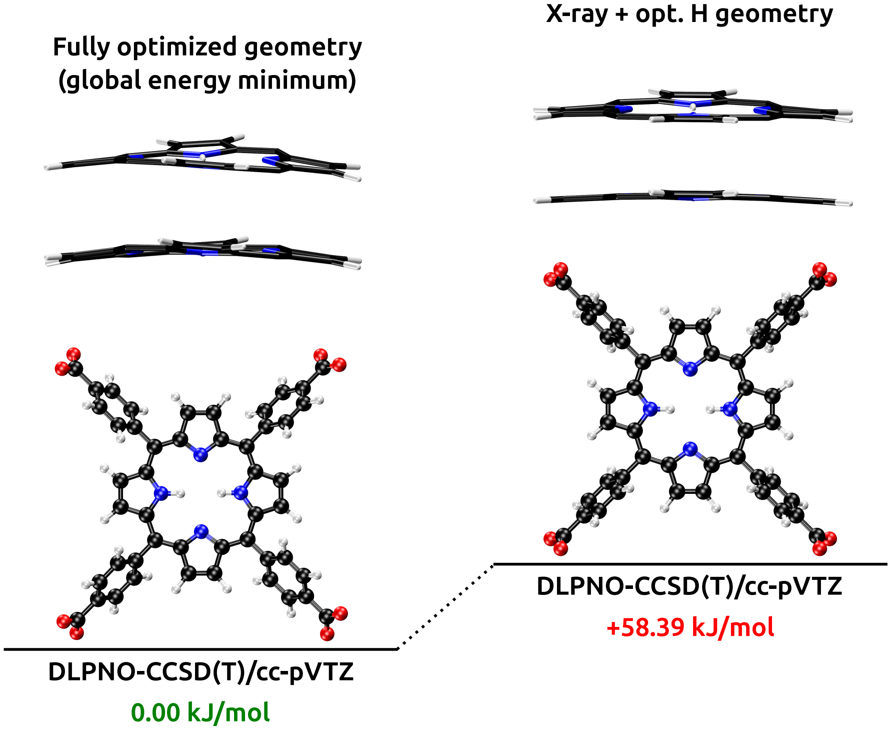

Figure S5: DLPNO-CCSD(T)/cc-pVTZ energy difference (kJ/mol) between fully optimized geometry corresponding to the minimum at the potential energy surface at the PBE0-D4/def2-TZVP level of theory (verified with Hessian analysis) and X-ray + opt. H geometry. To better visualize deformation of the porphyrin core, phenyl groups were removed for clarity in the upper panels.

DLPNO-CCSD(T)/cc-pVTZ calculations shown in Figure S5 confirm the well known propensity of substituted porphyrins to nonplanar (ruffle/saddle) distortions.[58–61] Geometry optimization of TCPP results in considerably distorted porphyrin core, which is energetically favored by nearly 60 kJ/mol compared to more flat, X-ray diffraction derived TCPP linker geometry in PCN-224 MOF. Moreover, solid-state $^{1}H-^{13}C$ CPMAS NMR spectrum of PCN-224 MOF (Figure 2a in the manuscript) reveals relatively broad resonances and overall decreased resolution compared to the spectrum of solid TCPP. This could indicate distribution of ^{13}C NMR shifts due to several coexisting linker distortions present in the PCN-224 MOF. Inspection of ^{13}C NMR shifts calculated for both geometries of Figure S5 at accurate DLPNO-DSD-PBEP86 and DLPNO-MP2 levels of theory (Table S2) does not reveal substantial differences. Notably, for $^{13}C_{meso}$ signal, which is clearly resolved in experimental spectrum of PCN-224 MOF at 119 ppm, optimized (distorted) geometry provides slightly better agreement with experiment at both DLPNO-DSD-PBEP86 and DLPNO-MP2 levels of theory. The same is true for $^{13}C_{\beta}$ observed at 128−130 ppm in the experimental spectrum. Since C_{meso} and C_{β} positions are located within the porphyrin core (not in phenyl ligands), and are therefore susceptible to effects of nonplanar distortions, this could indicate from the ^{13}C NMR standpoint that optimized linker geometry is more accurate compared to X-ray diffraction derived linker geometry. X-ray diffraction derived linker geometry of the Fe@PCN-224 MOF (see Figure S6) suggest simultaneous distortions, with their effects being summed up (superimposed) to create the final, observed structure with doubly occupied Fe and Cl sites. Finally, Hessian analysis indicate large-scale molecular motions/librations for all TCPP, Fe(III)Cl@TCPP, Fe(III)OH@TCPP, Fe(II)@TCPP models, as revealed by intense vibrations in a low-frequency range from 7 to 40 cm^{-1}; see Tables S16 and S17.

Comparison of Fe(III)Cl@TCPP geometries optimized with different DFT approximations and basis sets (Table S3) reveals balanced performance of the PBE0-D4/def2-TZVP level of theory, which provides overall best C−N and Fe−Cl distances, and slightly too short Fe−N bond lengths. On the other hand, GGA PBE-D4 approximation gives too long bond lengths for this system, even with the large def2-QZVP basis set.

Table S2: Isotropic ^{13}C NMR shifts (δ, ppm) calculated for the fully optimized (PBE0-D4/def2-TZVP) and the X-ray + opt. H geometries at DLPNO-DSD-PBEP86 and DLPNO-MP2 level of theory with the pcSseg-1 basis set.

	Fully optimized geometry		X-ray + opt. H geometry	
Atom	DLPNO-DSD-PBEP86	DLPNO-MP2	DLPNO-DSD-PBEP86	DLPNO-MP2
C_α	143.2	141.3	144.5	142.4
C_β	128.1	126.0	126.0	124.0
C_{meso}	121.5	122.1	123.2	123.7
C_{ipso}	137.2	134.8	134.9	132.7
C_{ortho}	132.5	131.2	128.2	127.2
C_{meta}	125.1	122.8	123.0	120.5
C_{para}	144.3	144.3	138.0	138.4
C_{COO^-}	159.1	157.0	164.5	162.0

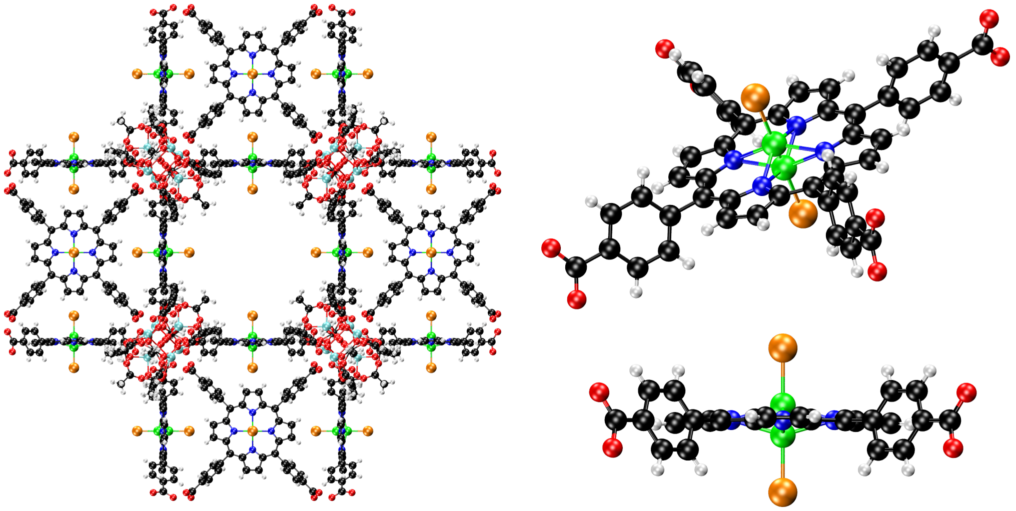

Figure S6: Fragments of crystal structure of the Fe@PCN-224 MOF generated from X-ray diffraction data by Harris.[1]

Table S3: C−N, Fe−N and Fe−Cl distances (Å) for the Fe(III)Cl@TCPP model energy-optimized with different DFT approximations and basis sets. Experimental data by Harris.[1]

Approximation	Basis set	$\overline{r}_{\mathrm{C-N}}$	$r_{\mathrm{Fe-N}}$	$r_{\mathrm{Fe-Cl}}$
PBE-D4	def2-TZVP	1.379	2.099; 2.100; 2.102; 2.103	2.238
PBE-D4	def2-QZVP	1.380	2.098; 2.099; 2.100; 2.102	2.237
PBE0-D4	def2-TZVP	1.369	2.071; 2.071; 2.073; 2.074	2.224
TPSSh-D4	def2-TZVP	1.377	2.077; 2.077; 2.078; 2.079	2.230
B3LYP-D4	def2-TZVP	1.374	2.079; 2.080; 2.083; 2.083	2.243
Experiment				
(X-ray; Fe@PCN-224 MOF)		1.370±0.006	2.086±0.006; 2.088±0.006	2.227±0.02

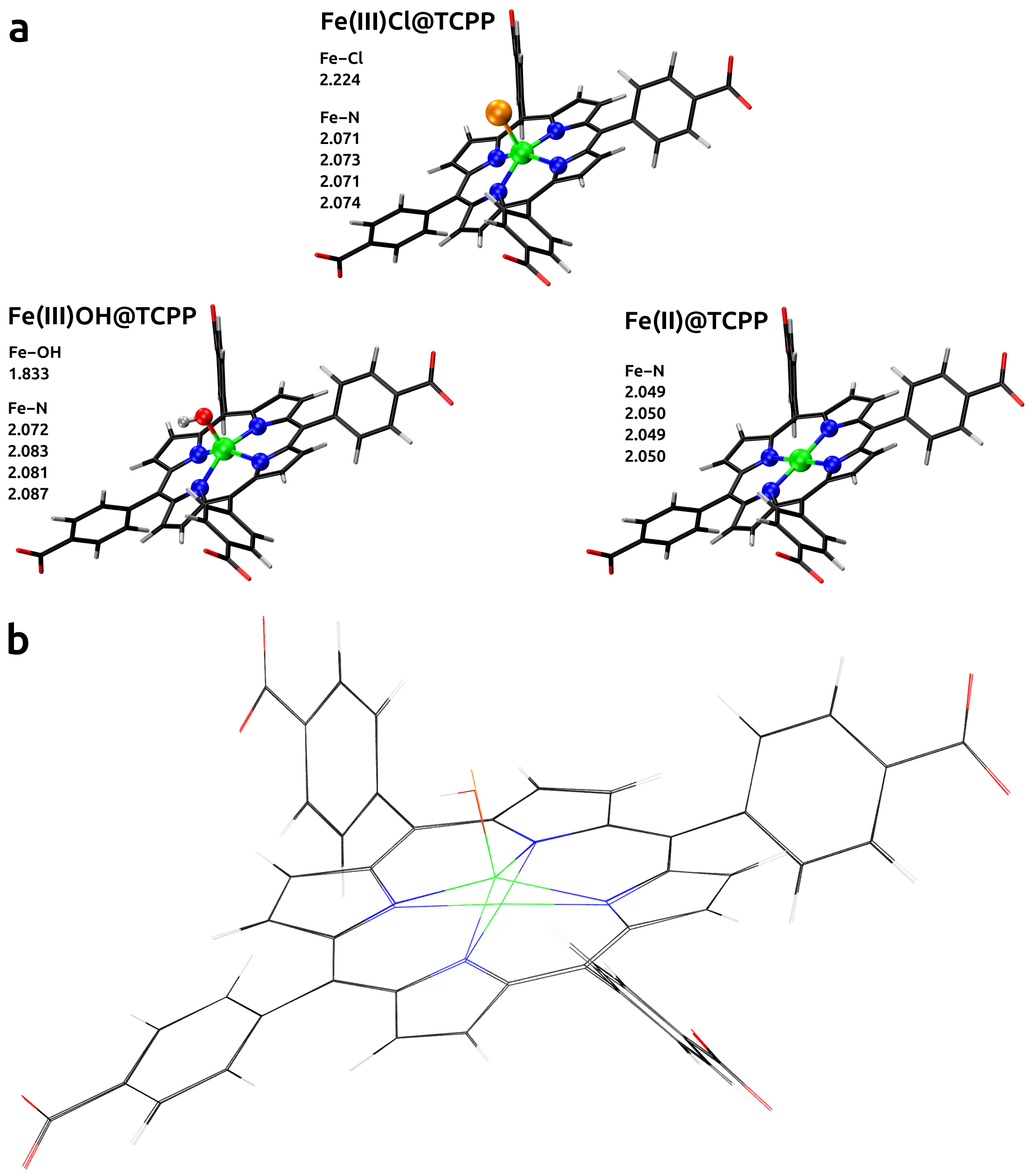

Figure S7: Geometry-optimized models corresponding to minima at the respective potential energy surfaces at the PBE0-D4/def2-TZVP level of theory, which were used in this work for EPR/NMR computations (panel a); coordinates of all 3 models overlapped (panel b).

S6. Prediction of hyperfine coupling constants: the challenge

The high sensitivity of NMR shifts of ligand atoms to changes in geometry within the coordination sphere of a paramagnetic metal ion was shown by Bertarello and coworkers,[62] on the example of Co(II)-binding site in superoxide dismutase metalloprotein (SOD). However, it is the reliable interpretation of the observed NMR shifts into chemical information, which still constitutes a limiting factor for usage of solid-state NMR data for structure determination/refinement in paramagnetic systems. As shown in our previous study, calculations of $\boldsymbol{A}$, and especially its isotropic component A_{iso}, the hyperfine coupling constant, constitute the most challenging task in interpretation of paramagnetic NMR shielding.[25] Kohn-Sham DFT of approximate exchange-correlation energy functionals is a workhorse of computational chemistry due to its good trade-off between computational cost and accuracy for most valence properties, however, its predictive power for hyperfine couplings is severely limited, and calculations with more rigorous methods are typically needed.[63–66] Hyperfine coupling is an exceedingly subtle property resulting from the interplay between orbital occupations, core- and valence-shell spin polarization and electron correlation effects. These impose extreme challenges to approximate density functionals, and so far, there seem to be a rather limited room for systematic improvements in this respect, although the work on better understanding of these limitations is ongoing.[66, 67] On the other hand, coupled-cluster (CC) theory offers the most accurate results among practical *ab initio* electronic-structure theories.[68, 69] However, CC singles and doubles CCSD model, and its counterpart including perturbative triples correction CCSD(T) exhibit steep polynomial scaling proportional to N^6 and N^7, respectively. Although these methods provide consistently accurate description of hyperfine couplings, can only be applied to study small benchmark systems.[25, 63, 64, 70] But this has changed by the advent of Domain-Based Pair Natural Orbital DLPNO-CCSD method, which offers access to hyperfine tensors that converge towards canonical CCSD limit at significantly reduced computational cost.[71–73] This offers the possibility of systematic control of errors, bridging the gap between computationally efficient but inconsistent Kohn-Sham DFT approximations, and highly accurate electronic structure theory calculations.[74]

We checked the convergence of our DLPNO-CCSD "production setup" that was used for analysis of Fe@PCN-224 MOF by calculating hyperfine couplings for the $[Fe(NH_3)_6]^{3+}$ complex, which was small enough so canonical CCSD calculation using the same core-polarized basis sets could be afforded. For comparison we also included results obtained with the hybrid DFT-PBE0 method used by Bertarello and coworkers in their work on Co(II)-SOD;[62] these results are shown in Table S4. Results by the DLPNO-CCSD setup were close to those by CCSD, but both ^{57}Fe and ^{14}N couplings were slightly underestimated with respect to CCSD. We checked that tightening the PNO-space truncation parameters in the DLPNO-CCSD calculation to HFC2 setting in ORCA code improves convergence of ^{14}N A_{iso} to 6.667 MHz (within 0.16 MHz from canonical CCSD), however, at very significant additional computational cost. On the contrary, $[Fe(NH_3)_6]^{3+}$ complex imposed an extreme challenge for the hybrid DFT-PBE0 model, and this method was not able to predict correct spin-density on the metal ion; EPR and ENDOR experiments on isolated $^{57}Fe^{+3}$ centers in α-quartz and in CaO revealed negative hyperfine couplings for ^{57}Fe of -27 and -30 MHz.[75, 76] DLPNO-CCSD method is capable of providing results in agreement with EPR and ENDOR data for the challenging ^{57}Fe nucleus, however, very large basis sets including critical core-correlation are necessary to provide converged results, as indicated in Table S4. We note that such calculations are not feasible with the canonical CCSD method due to enormous memory and integral storage requirements.

We also compared A_{iso} values calculated with the DFT-PBE0 approximation to those obtained with the DLPNO-CCSD method for the Fe(III)Cl@TCPP model, and resulting δ_{iso} values are shown in Table S5. Interestingly, hybrid DFT-PBE0 model was surprisingly accurate for the $^1H_\beta$, $^{13}C_\beta$ and $^{13}C_\alpha$ NMR shifts, whereas predictions for the remaining proton and carbon signals were essentially erratic; see Figure S8a. This inconsistent behavior of the DFT-PBE0 model resulted in virtually no correlation with experimental data ($R^2 = 0.388$), in contrast to excellent correlation ($R^2 = 0.976$) for the predictions by DLPNO-CCSD; see Figure S8b. These results corroborate observations from our previous study that DFT should not be used for structure refinements employing evaluation of paramagnetic NMR shifts, and the DLPNO-CCSD method should be used instead.[25]

Underestimation of computed $^1H_\beta$ and $^{13}C_\beta$ shifts for the Fe(III)Cl@TCPP model when using hyperfine tensors obtained by the DLPNO-CCSD is consistent with underestimated hyperfine couplings for the $[Fe(NH_3)_6]^{3+}$ complex at the same level of theory with respect to CCSD. It indicates that PNO-space truncation in the DLPNO-CCSD setup employed herein is probably a main source of errors, but those due to basis sets incompleteness may also be quite significant, although ^{14}N is less sensitive in this respect than ^{57}Fe. Nonetheless, both $^1H_\beta$ and $^{13}C_\beta$ signals exhibited the highest detected, and the highest calculated shifts for

protons and carbon, respectively, so their assignment is unambiguous despite the slight underestimation of hyperfine couplings.

Table S4: Calculated isotropic hyperfine coupling constants (A_{iso}; MHz) for the high-spin $[Fe(NH_3)_6]^{3+}$ complex with DFT-PBE0, DLPNO-CCSD and canonical CCSD methods using as indicated basis sets.

Method	^{57}Fe	^{14}N	Basis set Fe/N,H	Coupled-cluster amplitudes	Time factor
DFT-PBE0	12.836	7.764	def2-TZVP	—	1
DFT-PBE0	1.160	7.727	cc-pwCVTZ/EPR-II	—	1
DLPNO-CCSD	−9.492	6.429	cc-pwCVTZ/EPR-II	710338	×171
CCSD	−9.820	6.826	cc-pwCVTZ/EPR-II	212891230	×5030
DLPNO-CCSD	−22.572	6.046	cc-pwCVQZ/EPR-III	1216049	×272
DLPNO-CCSD	−27.059	6.142	cc-pwCV5Z/EPR-III	1436214	×380
DLPNO-CCSD	−26.746	5.986	aug-cc-pVTZ-J	1128118	×480

Table S5: Calculated isotropic hyperfine coupling constants (A_{iso}; MHz) and isotropic NMR shifts (δ_{iso}; ppm) evaluated for the Fe(III)Cl@TCPP model using hyperfine tensors $\boldsymbol{A}$ calculated with the DFT-PBE0 model and at the DLPNO-CCSD level of theory using the cc-pwCVTZ(Fe,Cl)/EPR-II(N,C,H) basis sets and the same remaining parameters ($\boldsymbol{g}$, $\boldsymbol{D}$, and $\boldsymbol{\sigma}_{\text{orb}}$).

Atom	A_{iso} DFT-PBE0	A_{iso} DLPNO-CCSD	δ_{iso} DFT-PBE0	δ_{iso} DLPNO-CCSD	δ_{iso} Experiment
$^{1}H_{\beta}$	0.243	0.123	78.3	43.8	73.0
$^{1}H_{ortho}$	−0.463	−0.006	−122.3	6.1	6.8
$^{1}H_{meta}$	0.466	0.004	144.3	11.5	13.0
$^{13}C_{\alpha}$	0.574	0.542	795.1	759.3	810
$^{13}C_{\beta}$	1.138	0.692	1433.7	923.2	1212
$^{13}C_{meso}$	0.715	0.228	945.0	385.9	341
$^{13}C_{ipso}$	−1.071	−0.122	−1069.6	10.4	−15
$^{13}C_{ortho}$	1.056	0.082	1344.9	234.5	227
$^{13}C_{meta}$	−0.758	−0.003	−726.1	130.8	146
$^{13}C_{para}$	0.828	0.009	1095.2	162.3	168
$^{13}C_{COO^-}$	−0.256	−0.003	−121.1	167.5	168
^{14}N	9.541	8.350			
^{35}Cl	7.077	7.816			
^{57}Fe	1.851	−3.719			
Correlation with experiment (R^2)			0.388	0.976	1.000

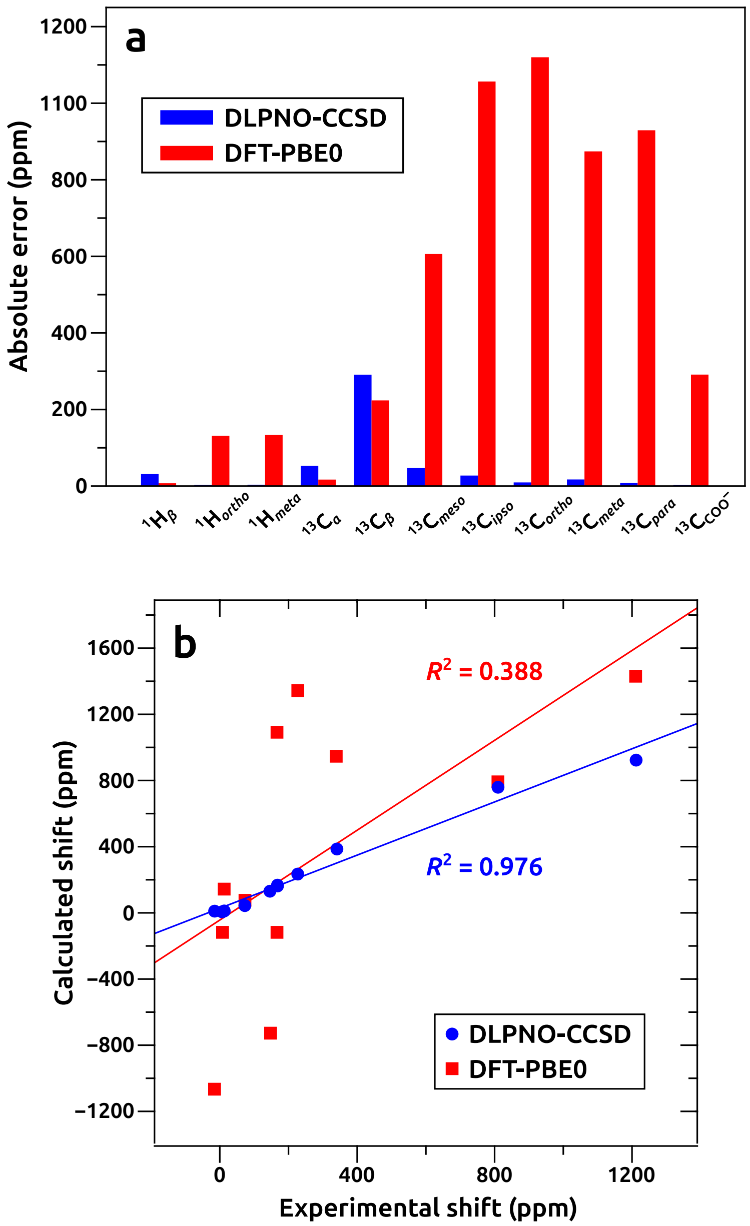

Figure S8: Absolute errors with respect to experimental data (panel a) and correlation with experimental data (panel b) for isotropic NMR shifts ($\delta_{\rm iso}$; ppm) evaluated for the Fe(III)Cl@TCPP model using hyperfine tensors $\boldsymbol{A}$ calculated with the DFT-PBE0 model and at the DLPNO-CCSD level of theory using the same cc-pwCVTZ(Fe,Cl)/EPR-II(N,C,H) basis sets and the same remaining parameters ($\boldsymbol{g}$, $\boldsymbol{D}$, and $\boldsymbol{\sigma}_{\rm orb}$).

Table S6: $\boldsymbol{g}$ and $\boldsymbol{D}$ (cm^{-1}) tensors calculated at the DKH2-CASSCF/NEVPT2/cc-pwCVTZ-DK(Fe)/cc-pVDZ-DK(Cl,O,N,C,H) level of theory for the Fe(III)Cl@TCPP, Fe(III)OH@TCPP, and Fe(II)@TCPP models.

Model	g_{xx}	g_{yy}	g_{zz}	D_{xx}	D_{yy}	D_{zz}
Fe(III)Cl@TCPP	2.000827	2.000835	2.001589	−0.037527	−0.085257	2.066913
Fe(III)OH@TCPP	2.001172	2.001191	2.001647	0.080441	−0.137584	1.313466
Fe(II)@TCPP	1.985718	2.296393	2.299626	0.050341	−0.110945	30.675816

Table S7: Isotropic NMR shifts ($\delta_{\rm iso}$; ppm) evaluated for the Fe(III)Cl@TCPP, Fe(III)OH@TCPP, and Fe(II)@TCPP models using $\boldsymbol{g}$ and $\boldsymbol{D}$ tensors from Table S6 and $\boldsymbol{A}$ tensors from Tables S10 and S11.

Atom	$\delta_{\rm iso}$ Fe(III)Cl@TCPP	$\delta_{\rm iso}$ Fe(III)OH@TCPP	$\delta_{\rm iso}$ Fe(II)@TCPP
$^{1}H_{\beta}$	43.8	38.8	15.8
$^{1}H_{ortho}$	6.1	7.1	−3.3
$^{1}H_{meta}$	11.5	11.7	6.1
$^{13}C_{\alpha}$	759.3	745.4	952.9
$^{13}C_{\beta}$	923.2	832.1	544.6
$^{13}C_{meso}$	385.9	233.9	1.4
$^{13}C_{ipso}$	10.4	33.7	142.4
$^{13}C_{ortho}$	234.5	206.8	114.3
$^{13}C_{meta}$	130.8	129.4	129.4
$^{13}C_{para}$	162.3	161.6	145.6
$^{13}C_{\rm COO^-}$	167.5	168.0	169.3

Ability to differentiate between Fe−Cl and Fe−OH axial ligands via NMR signatures of ligand atoms of the porphyrin ring is clearly advantageous, as proton positions typically remain invisible in X-ray diffraction, and preparation of large enough batch of deuterated Fe@PCN-224 MOF sample for neutron diffraction would be challenging. From NMR perspective, detection of the Fe-OH proton would be challenging due to close distance to the Fe ion and therefore potential substantial paramagnetic effects (extensive signal broadening). We calculated hyperfine coupling of $A_{\rm iso}$ = 2.798 MHz for the $^{1}H_{\rm Fe-OH}$ nucleus in the Fe(III)OH@TCPP model.

S7. Calculations for the Fe(II)(py-NMe-PiPr$_2$)Cl$_2$ catalyst

As an additional test for our theoretical protocol for interpretation of paramagnetic NMR shifts, we explored the Fe(II)(py-NMe-PiPr$_2$)Cl$_2$ catalyst molecule [$FeCl_2N_2PC_{13}H_{22}$] with exactly same calculations as performed for our Fe@PCN-224 MOF models. Optimized geometry of the Fe(II)(py-NMe-PiPr$_2$)Cl$_2$ is shown in Figure S9 panel a, and spin densities calculated for this molecule are rendered in panels b−g.

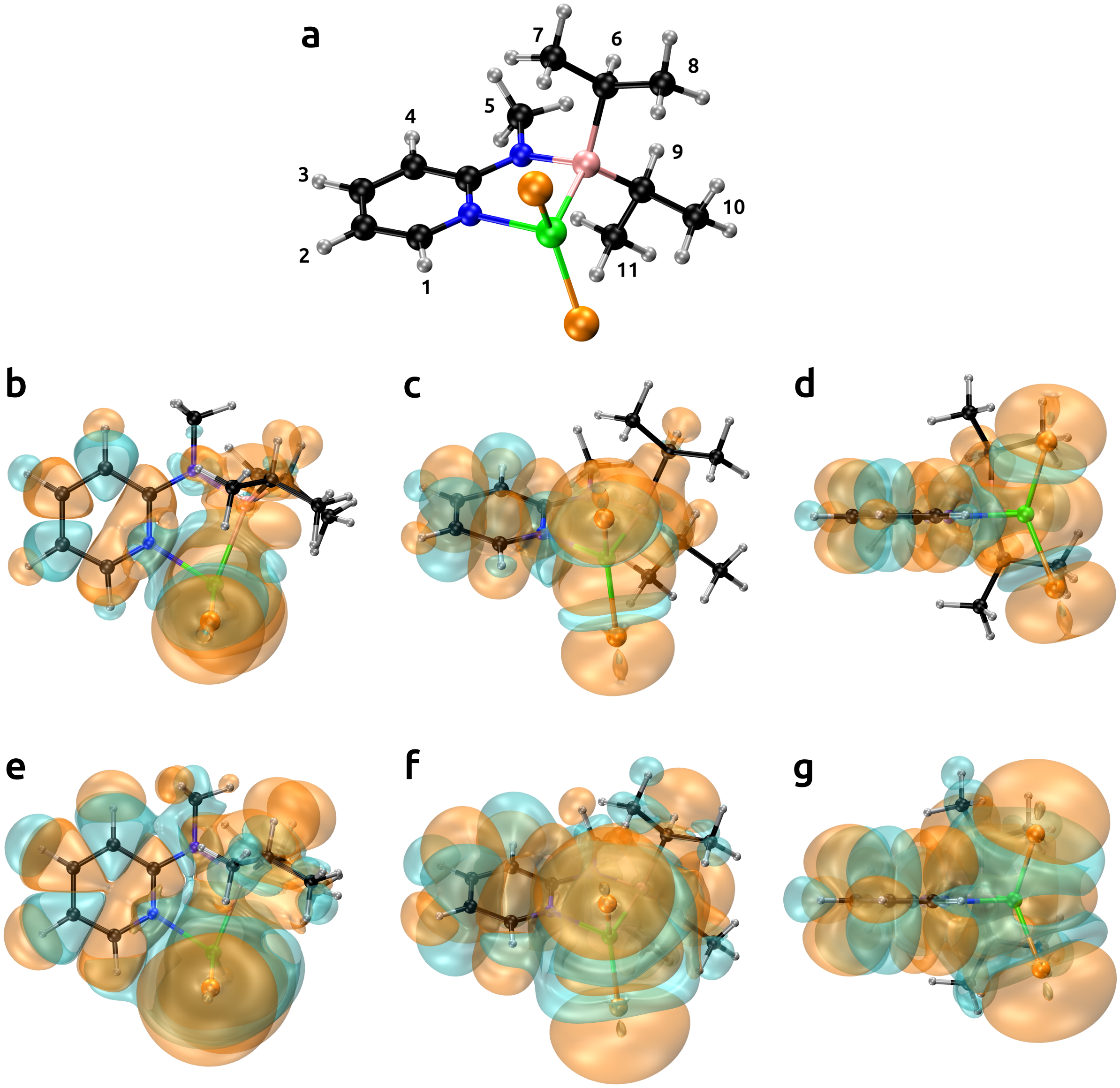

Figure S9: Optimized geometry of the Fe(II)(py-NMe-PiPr$_2$)Cl$_2$ catalyst (panel a). Spin densities calculated at the DLPNO-CCSD level of theory using the cc-pwCVTZ(Fe,Cl,P)/EPR-II(N,C,H) basis sets and rendered with an isodensity of 0.0001 (panels b−d), and 0.00001 (e−g) e bohr^{-3}; orange and cyan denote the positive and negative regions, respectively.

Experimental ^{1}H NMR shifts of this system were recently reported by Koppe and coworkers.[5] Our theoretical data in Table S8 suggest reassignment of experimental $^1H_6$ and $^1H_9$ isotropic NMR shifts. Note that $^1H_6$ is encoded in dark blue and $^1H_9$ in light blue in Fig. 2 of ref.[5] This reassignment is consistent with ^{13}C MAS NMR data reported previously by Blahut and coworkers[77] for the same system, where isotropic shift of carbon $^{13}C{-}H_6$ is higher compared to carbon $^{13}C{-}H_9$, in agreement with the higher hyperfine coupling (A_{iso}) for $^1H_6$ compared to $^1H_9$ (Table S8). Reassignment of $^1H_6$ and $^1H_9$ NMR shifts substantially improves correlation between theory and experiment from $R^2 = 0.958$ to $R^2 = 0.980$; see Figure S10a,b.

Further improvements come from inclusion of VPT2 vibrational corrections to isotropic hyperfine couplings ($A_{\mathrm{iso}}^{\mathrm{zpvc}}$), and significantly improved correlation of $R^2 = 0.989$ is obtained; see Figure S10c.

We note relatively small anisotropy of the $\boldsymbol{g}$ tensor for the Fe(II) ion in the Fe(py-NMe-PiPr$_2$)Cl$_2$ complex, and also limited magnitude of the zero-field splitting $\boldsymbol{D}$ tensor (Table S9). These are significantly smaller than corresponding values for the close-to-planar Fe(II)@TCPP porphyrin complex (Table S6). Notably, it is also revealed when considering shift anisotropies of proton positions with the strongest hyperfine couplings in the Fe(II)(py-NMe-PiPr$_2$)Cl$_2$ molecule and the Fe(III)Cl@TCPP MOF linker model, which correspond to ^{1}H$_6$ and ^{1}H$_\beta$ sites, respectively. Calculated shift anisotropies of $\Delta\delta\,^1\mathrm{H}_6 = 524$ ppm and $\Delta\delta\,^1\mathrm{H}_\beta = 452$ ppm are quite similar, or, in other words, there are no substantial differences, which typically would be expected between Fe(III) and Fe(II) electronic configurations. These results indicate that it should not be assumed that Fe(II) ion must always exhibit pronounced spin-orbit coupling and therefore significant magnetic anisotropy.

Inspection of DLPNO-CCSD distribution of unpaired electron spin densities as presented in Figure S9 panels b−g is already helpful for understanding of experimental NMR shifts from paramagnetic complexes, even without detailed paramagnetic NMR shifts calculations. For example, examination of the top view on the aromatic ring fragment shown in panel e clearly indicates negative region on proton H$_3$ (in contrast to its neighbors), which is in agreement with negative hyperfine coupling, and therefore negative NMR shift, in Table S8. Similarly, inspection of panels c and f indicates positive regions on H$_6$/H$_9$ positions, and negative regions on the corresponding methyl groups $\mathrm{H}_{7/8-\mathrm{CH}_3}/\mathrm{H}_{9/10-\mathrm{CH}_3}$, which is in agreement with positive and negative NMR shifts, respectively, observed for these species in Table S8.

Table S8: Calculated ^{1}H isotropic hyperfine coupling constants (A_{iso}; MHz) and vibrational corrections ($A_{\mathrm{iso}}^{\mathrm{zpvc}}$; MHz) for the Fe(II)(py-NMe-PiPr$_2$)Cl$_2$ catalyst molecule and isotropic ^{1}H NMR shifts evaluated with $\boldsymbol{g}$ and $\boldsymbol{D}$ tensors from Table S9. Experimental ^{1}H NMR shifts from Koppe and coworkers.[5] ^{a} shifts of ^{1}H$_6$ and ^{1}H$_9$ were reassigned.

Atom	A_{iso}	$A_{\mathrm{iso}}^{\mathrm{zpvc}}$	δ_{iso}	δ_{iso}+VPT2	Experiment (δ_{iso})
^{1}H$_1$	0.367	0.025	73.6	79.3	109
^{1}H$_2$	0.079	0.020	23.1	27.7	44
^{1}H$_3$	−0.053	−0.031	−4.3	−11.3	−37
^{1}H$_4$	0.165	0.026	42.6	48.2	62
^{1}H$_6$	0.661	0.049	146.5	157.4	283^a
^{1}H$_9$	0.563	0.089	124.7	144.6	258^a
$^1\mathrm{H}_{5-\mathrm{CH}_3}$	0.012	0.008	4.4	6.2	11
$^1\mathrm{H}_{7-\mathrm{CH}_3}$	−0.013				
$^1\mathrm{H}_{8-\mathrm{CH}_3}$	−0.010	0.015	−5.5	−1.6	−6
$^1\mathrm{H}_{10-\mathrm{CH}_3}$	−0.004				
$^1\mathrm{H}_{11-\mathrm{CH}_3}$	−0.004				
Correlation with experiment (R^2)			0.980	0.989	1.000

Table S9: $\boldsymbol{g}$ and $\boldsymbol{D}$ (cm^{-1}) tensors calculated at the DKH2-CASSCF/NEVPT2/cc-pwCVTZ-DK(Fe)/cc-pVDZ-DK(Cl,P,N,C,H) level of theory for the Fe(II)(py-NMe-PiPr$_2$)Cl$_2$ catalyst.

g_{xx}	g_{yy}	g_{zz}	D_{xx}	D_{yy}	D_{zz}
2.021077	2.088081	2.095511	−0.310054	−3.438475	3.748530

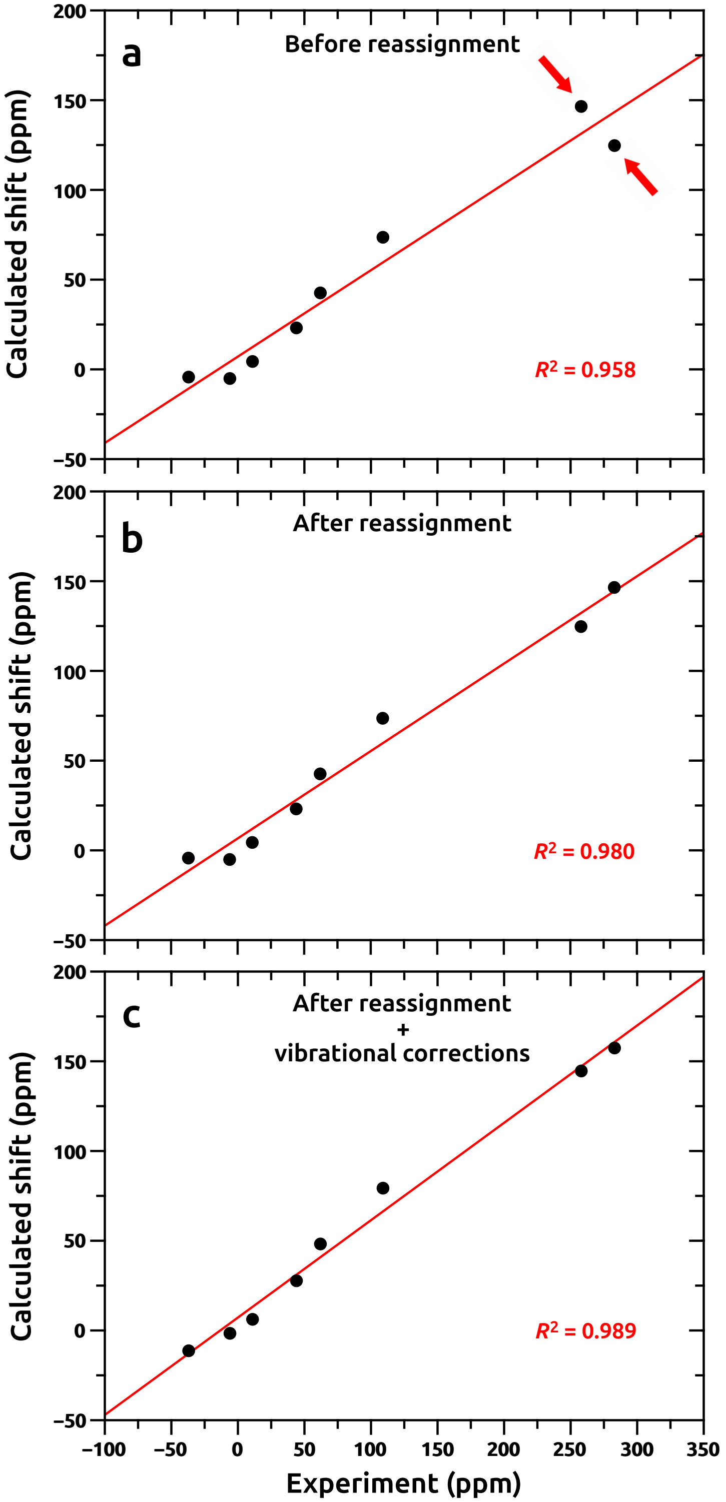

Figure S10: Correlation between theory and experiment for isotropic ^{1}H NMR shifts before (panel a) and after reassignment of $^1H_6/^1H_9$ signals (b) from the Fe(II)(py-NMe-PiPr$_2$)Cl$_2$ catalyst, as well as improvements due to inclusion of vibrational corrections to isotropic hyperfine couplings (c).

S8. Examples of input commands for the ORCA code

In this section we present examples of input commands for the ORCA code, on the example of Fe(II)(py-NMe-PiPr$_2$)Cl$_2$ complex discussed above. We direct reader to official documentation of ORCA for detailed understanding of the keywords used. In this work ORCA version 5.0.1 was used, and note there might be differences between different versions, for example for the default integration grid settings, which might affect numerical precision.

Geometry optimization — **ORCA.inp**

```
!  PBE0 D4 def2-TZVP def2/J RIJCOSX NoTRAH NoSOSCF TightSCF VerySlowConv TightOpt

%maxcore 6000

%pal
nprocs 40
end

%scf
MaxIter 5000
end

%geom
MaxIter 500
end

* xyz 0 5
Fe 5.918436  13.931969  8.074312
Cl 4.412047  15.402638  7.379218
Cl 5.822149  11.751791  8.476365
P  7.536478  14.881036  9.576513
N  8.885790  15.021302  8.515279
C  8.735235  14.769651  7.169439
N  7.561095  14.279798  6.748603
C  7.368505  14.043573  5.451583
C  8.329245  14.269901  4.491507
C  9.551710  14.768487  4.918212
C  9.767236  15.022843  6.255546
H  10.714799 15.421911  6.584344
H  10.342775 14.967490  4.204259
H  8.123688  14.066582  3.449827
H  6.387826  13.662112  5.189739
C  10.145605 15.557842  8.979617
H  10.341817 16.554850  8.570321
H  10.125210 15.637620  10.064797
H  10.978288 14.900957  8.712806
C  8.174955  13.865090  10.976706
C  6.998086  13.322210  11.786537
H  7.378591  12.756008  12.640486
H  6.344357  14.104058  12.171931
H  6.396342  12.649088  11.171809
C  9.014848  12.706204  10.451377
H  9.316192  12.070484  11.287828
H  9.918508  13.037981  9.939693
H  8.431988  12.092514  9.759751
H  8.788949  14.511158  11.616971
C  7.346182  16.605624  10.195769
C  7.151471  17.538103  9.005290
H  7.047282  18.566308  9.361211
H  7.992532  17.509505  8.310149
H  6.246074  17.277872  8.450844
C  6.168038  16.711048  11.157998
H  6.001687  17.760563  11.413313
H  6.338069  16.171031  12.089491
H  5.252119  16.333222  10.696283
H  8.267395  16.874776  10.727447
*
```

Numerical Hessian — **ORCA.inp**

```
!  PBE0 D4 def2-TZVP def2/J RIJCOSX NoTRAH NoSOSCF TightSCF VerySlowConv NumFreq

%maxcore 6000

%pal
nprocs 40
end

%scf
MaxIter 5000
end

* xyz 0 5
atomic coordinates
*
```

A tensor

ORCA.inp

```
!  DLPNO-CCSD NormalPNO RIJCOSX NoTRAH TightSCF SOSCF VerySlowConv NoFrozenCore

%maxcore 12000

%pal
nprocs 20
end

%scf
MaxIter 5000
SOSCFStart 0.0001
SOSCFMaxIt 200
DIISMaxEq 20
DirectResetFreq 1
end

%mdci
Density Unrelaxed
MaxIter 1000
LocMaxIterLed 10000
end

%basis
NewGTO Fe "cc-pwCVTZ" end
NewGTO Cl "cc-pwCVTZ" end
NewGTO P "cc-pwCVTZ" end
NewGTO N "EPR-II" end
NewGTO C "EPR-II" end
NewGTO H "EPR-II" end
NewAuxCGTO Fe "cc-pwCVTZ/C" end
NewAuxCGTO Cl "cc-pwCVTZ/C" end
NewAuxCGTO P "cc-pwCVTZ/C" end
NewAuxCGTO N "cc-pwCVDZ/C" end
NewAuxCGTO C "cc-pwCVDZ/C" end
NewAuxCGTO H "cc-pwCVDZ/C" end
NewAuxJGTO Fe "def2/J" end
NewAuxJGTO Cl "def2/J" end
NewAuxJGTO P "def2/J" end
NewAuxJGTO N "def2/J" end
NewAuxJGTO C "def2/J" end
NewAuxJGTO H "def2/J" end
end

* xyz 0 5
atomic coordinates
*

%eprnmr
Nuclei = all Fe { Aiso, Adip }
Nuclei = all Cl { Aiso, Adip }
Nuclei = all P  { Aiso, Adip }
Nuclei = all N  { Aiso, Adip }
Nuclei = all C  { Aiso, Adip }
Nuclei = all H  { Aiso, Adip }
end
```

Starting orbitals for CASSCF

ORCA.inp

```
!  DKH2 PBE0 AutoAux RIJCOSX NoTRAH SOSCF TightSCF VerySlowConv UNO Normalprint
# !  DKH2 PBE0 AutoAux RIJCOSX Normalprint NoIter MORead # for inspection of QROs:  Löwdin reduced orbital analysis
# %moinp "job1.qro" # read in QROs from the first job

%maxcore 6000

%pal
nprocs 40
end

%scf
MaxIter 5000
SOSCFStart 0.00001
SOSCFMaxIt 200
DIISMaxEq 20
DirectResetFreq 1
end

%basis
NewGTO Fe "cc-pwCVTZ-DK" end
NewGTO Cl "cc-pVDZ-DK" end
NewGTO P "cc-pVDZ-DK" end
NewGTO N "cc-pVDZ-DK" end
NewGTO C "cc-pVDZ-DK" end
NewGTO H "cc-pVDZ-DK" end
end

* xyz 0 5
atomic coordinates
*
```

g and D tensor

ORCA.inp

```
!  DKH2 NEVPT2 AutoAux RIJCOSX NoTRAH TightSCF VerySlowConv NoFrozenCore NormalPrint MORead

%moinp "start.gbw"

%maxcore 6000

%pal
nprocs 40
end

%scf
MaxIter 5000
SOSCFStart 0.0001
SOSCFMaxIt 200
DIISMaxEq 20
DirectResetFreq 1
end

%casscf
nel 6
norb 5
mult 5,3,1
nroots 5,20,30
OrbStep SuperCI_PT
SwitchStep NR
SwitchIter 20
MaxIter 200
rel
dosoc true
gtensor true
dtensor true
end
end

%rel
PictureChange 2
FiniteNuc True
end

%basis
NewGTO Fe "cc-pwCVTZ-DK" end
NewGTO Cl "cc-pVDZ-DK" end
NewGTO P "cc-pVDZ-DK" end
NewGTO N "cc-pVDZ-DK" end
NewGTO C "cc-pVDZ-DK" end
NewGTO H "cc-pVDZ-DK" end
end

* xyz 0 5
atomic coordinates
*
```

Orbital shielding (σ_{orb})

ORCA.inp

```
!  PBE0 pcSseg-2 def2/J RIJCOSX NoTRAH NoSOSCF TightSCF VerySlowConv NMR

%maxcore 12000

%pal
nprocs 20
end

%scf
MaxIter 5000
SOSCFMaxIt 200
DIISMaxEq 20
DirectResetFreq 1
end

* xyz 0 5
atomic coordinates
*
```

VPT2 input #1: anharmonic force-field — **ORCA.inp**

```
!  PBE D4 def2-SVP def2/J NoTRAH SOSCF ExtremeSCF VerySlowConv VPT2

%maxcore 6000

%pal
nprocs 40
end

%scf
MaxIter 5000
SOSCFStart 0.0001
SOSCFMaxIt 200
DIISMaxEq 20
DirectResetFreq 1
end

%vpt2
VPT2 On
AvgProp ATensor
end

%method
Z_Tol 1e-12
end

* xyz 0 5
atomic coordinates
*
```

VPT2 input #2: A^{zpvc} — **ORCA.atensor.inp**

```
!  PBE0 EPR-II def2/J RIJCOSX NoTRAH SOSCF ExtremeSCF VerySlowConv

%maxcore 6000

%pal
nprocs 40
end

%scf
MaxIter 5000
SOSCFStart 0.0001
SOSCFMaxIt 200
DIISMaxEq 20
DirectResetFreq 1
end

* xyz 0 5
atomic coordinates
*
```

pNMR toolbox: paramagnetic shielding — **ORCA.pnmr.inp**

```
5 # Spin multiplicity (2S+1)
293 # Temperature range minimum (K)
293 # Temperature range maximum (K)
1 # Temperature step (K)
1 # Have g tensor?  (0 or 1)
2.021077 0.000000 0.000000 # Cartesian g tensor from CASSCF/NEVPT2
0.000000 2.088081 0.000000
0.000000 0.000000 2.095511
1 # Have D tensor?  (0 or 1)
-0.310054 0.000000 0.000000 # Cartesian D tensor (cm-1) from CASSCF/NEVPT2
0.000000 -3.438475 0.000000
0.000000 0.000000 3.748530
21 # Number of A tensors
11H # Nucleus (index, element)
533.5514 # Prefactor (MHz)
-0.3661 0.0000 0.0000 # Cartesian A tensor (MHz) from DLPNO-CCSD
0.0000 -0.3852 0.0000
0.0000 0.0000 1.2475
.
.
.
# Further nuclei
```

S9. Supplementary data: hyperfine tensors, atomic coordinates, vibrational frequencies

Table S10: ^{13}C hyperfine tensors $\boldsymbol{A}$ (MHz) calculated at the DLPNO-CCSD/cc-pwCVTZ(Fe,Cl)/EPR-II(O,N,C,H) level of theory for the Fe(III)Cl@TCPP, Fe(III)OH@TCPP, and Fe(II)@TCPP models.

	Fe(III)Cl@TCPP			Fe(III)OH@TCPP			Fe(II)@TCPP		
Atom	A_{xx}	A_{yy}	A_{zz}	A_{xx}	A_{yy}	A_{zz}	A_{xx}	A_{yy}	A_{zz}
0C	0.4425	0.0000	0.0000	0.3271	0.0000	0.0000	0.3803	0.0000	0.0000
	0.0000	0.4461	0.0000	0.0000	0.4225	0.0000	0.0000	0.4705	0.0000
	0.0000	0.0000	1.2598	0.0000	0.0000	1.1358	0.0000	0.0000	1.1795
1C	0.4333	0.0000	0.0000	0.1990	0.0000	0.0000	0.0318	0.0000	0.0000
	0.0000	0.4365	0.0000	0.0000	0.2926	0.0000	0.0000	0.1945	0.0000
	0.0000	0.0000	1.2455	0.0000	0.0000	1.0916	0.0000	0.0000	0.9913
2C	−0.1002	0.0000	0.0000	−0.0775	0.0000	0.0000	0.2229	0.0000	0.0000
	0.0000	0.1231	0.0000	0.0000	−0.1855	0.0000	0.0000	0.2536	0.0000
	0.0000	0.0000	2.1423	0.0000	0.0000	1.9435	0.0000	0.0000	2.3294
3C	0.0699	0.0000	0.0000	−0.2381	0.0000	0.0000	−0.0352	0.0000	0.0000
	0.0000	−0.1633	0.0000	0.0000	−0.5381	0.0000	0.0000	0.2727	0.0000
	0.0000	0.0000	2.1090	0.0000	0.0000	1.7748	0.0000	0.0000	2.4080
4C	−0.1268	0.0000	0.0000	0.0655	0.0000	0.0000	0.3577	0.0000	0.0000
	0.0000	−0.3854	0.0000	0.0000	0.1578	0.0000	0.0000	0.4493	0.0000
	0.0000	0.0000	1.9087	0.0000	0.0000	2.1221	0.0000	0.0000	2.4614
5C	0.4281	0.0000	0.0000	0.3271	0.0000	0.0000	0.1838	0.0000	0.0000
	0.0000	0.4573	0.0000	0.0000	0.3592	0.0000	0.0000	0.2158	0.0000
	0.0000	0.0000	1.2677	0.0000	0.0000	1.1639	0.0000	0.0000	1.0192
6C	0.3710	0.0000	0.0000	0.3351	0.0000	0.0000	0.2578	0.0000	0.0000
	0.0000	0.4036	0.0000	0.0000	0.3966	0.0000	0.0000	0.3529	0.0000
	0.0000	0.0000	1.2403	0.0000	0.0000	1.1633	0.0000	0.0000	1.0593
7C	−0.0979	0.0000	0.0000	−0.0699	0.0000	0.0000	0.3418	0.0000	0.0000
	0.0000	−0.3413	0.0000	0.0000	−0.1734	0.0000	0.0000	0.3912	0.0000
	0.0000	0.0000	1.9654	0.0000	0.0000	1.9627	0.0000	0.0000	2.5320
8C	−0.1270	0.0000	0.0000	0.0074	0.0000	0.0000	0.1710	0.0000	0.0000
	0.0000	−0.4309	0.0000	0.0000	0.0900	0.0000	0.0000	0.3224	0.0000
	0.0000	0.0000	1.9172	0.0000	0.0000	2.1434	0.0000	0.0000	2.4551
9C	0.3889	0.0000	0.0000	0.2590	0.0000	0.0000	0.2321	0.0000	0.0000
	0.0000	0.3947	0.0000	0.0000	0.3337	0.0000	0.0000	0.3565	0.0000
	0.0000	0.0000	1.2116	0.0000	0.0000	1.1464	0.0000	0.0000	1.0305
10C	0.3886	0.0000	0.0000	0.3418	0.0000	0.0000	0.0371	0.0000	0.0000
	0.0000	0.4065	0.0000	0.0000	0.4295	0.0000	0.0000	0.1398	0.0000
	0.0000	0.0000	1.2174	0.0000	0.0000	1.1469	0.0000	0.0000	0.9351
11C	−0.0692	0.0000	0.0000	−0.0599	0.0000	0.0000	0.3980	0.0000	0.0000
	0.0000	−0.3605	0.0000	0.0000	−0.3236	0.0000	0.0000	0.6007	0.0000
	0.0000	0.0000	1.9531	0.0000	0.0000	1.9524	0.0000	0.0000	2.4939
12C	0.0258	0.0000	0.0000	0.0790	0.0000	0.0000	−0.6649	0.0000	0.0000
	0.0000	−0.3225	0.0000	0.0000	−0.3815	0.0000	0.0000	0.6985	0.0000
	0.0000	0.0000	0.9821	0.0000	0.0000	0.9459	0.0000	0.0000	−0.9426
13C	−0.0375	0.0000	0.0000	−0.5157	0.0000	0.0000	−0.5020	0.0000	0.0000
	0.0000	−0.3209	0.0000	0.0000	−0.6062	0.0000	0.0000	−0.5671	0.0000
	0.0000	0.0000	0.9847	0.0000	0.0000	0.7802	0.0000	0.0000	0.8654
14C	−0.0467	0.0000	0.0000	0.1559	0.0000	0.0000	−0.3735	0.0000	0.0000
	0.0000	−0.3273	0.0000	0.0000	−0.3730	0.0000	0.0000	−0.4378	0.0000
	0.0000	0.0000	1.0203	0.0000	0.0000	1.0130	0.0000	0.0000	0.9992
15C	0.0696	0.0000	0.0000	−0.4383	0.0000	0.0000	−0.5647	0.0000	0.0000
	0.0000	−0.3203	0.0000	0.0000	−0.4440	0.0000	0.0000	−0.8166	0.0000
	0.0000	0.0000	1.0293	0.0000	0.0000	0.9080	0.0000	0.0000	0.8489
16C	−0.1558	0.0000	0.0000	−0.1919	0.0000	0.0000	−0.0215	0.0000	0.0000
	0.0000	−0.4047	0.0000	0.0000	−0.4881	0.0000	0.0000	0.2092	0.0000
	0.0000	0.0000	1.9083	0.0000	0.0000	1.8350	0.0000	0.0000	2.3509
17C	0.4038	0.0000	0.0000	0.2850	0.0000	0.0000	0.3163	0.0000	0.0000
	0.0000	0.4354	0.0000	0.0000	0.3503	0.0000	0.0000	0.5369	0.0000
	0.0000	0.0000	1.2468	0.0000	0.0000	1.1799	0.0000	0.0000	1.1216
18C	0.3931	0.0000	0.0000	0.3521	0.0000	0.0000	0.0220	0.0000	0.0000
	0.0000	0.4271	0.0000	0.0000	0.3878	0.0000	0.0000	0.1691	0.0000
	0.0000	0.0000	1.2563	0.0000	0.0000	1.2075	0.0000	0.0000	0.9665
19C	−0.0522	0.0000	0.0000	−0.2687	0.0000	0.0000	0.4244	0.0000	0.0000
	0.0000	−0.2749	0.0000	0.0000	−0.6052	0.0000	0.0000	0.7270	0.0000
	0.0000	0.0000	1.9922	0.0000	0.0000	1.7519	0.0000	0.0000	2.5603
20C	0.2030	0.0000	0.0000	−0.1723	0.0000	0.0000	−0.1594	0.0000	0.0000
	0.0000	−0.2665	0.0000	0.0000	−0.1822	0.0000	0.0000	−0.1644	0.0000
	0.0000	0.0000	−0.2860	0.0000	0.0000	0.2889	0.0000	0.0000	0.3301

Table S10: Continuation.

	Fe(III)Cl@TCPP			Fe(III)OH@TCPP			Fe(II)@TCPP		
Atom	A_{xx}	A_{yy}	A_{zz}	A_{xx}	A_{yy}	A_{zz}	A_{xx}	A_{yy}	A_{zz}
21C	−0.0242	0.0000	0.0000	−0.0969	0.0000	0.0000	−0.1010	0.0000	0.0000
	0.0000	−0.0402	0.0000	0.0000	−0.1030	0.0000	0.0000	−0.1018	0.0000
	0.0000	0.0000	0.2773	0.0000	0.0000	0.2158	0.0000	0.0000	0.2054
22C	−0.0588	0.0000	0.0000	−0.0464	0.0000	0.0000	−0.0517	0.0000	0.0000
	0.0000	−0.0673	0.0000	0.0000	−0.0505	0.0000	0.0000	−0.0531	0.0000
	0.0000	0.0000	0.1073	0.0000	0.0000	0.1139	0.0000	0.0000	0.1096
23C	−0.0203	0.0000	0.0000	−0.0445	0.0000	0.0000	−0.0434	0.0000	0.0000
	0.0000	−0.0376	0.0000	0.0000	−0.0522	0.0000	0.0000	−0.0448	0.0000
	0.0000	0.0000	0.0887	0.0000	0.0000	0.0804	0.0000	0.0000	0.0843
24C	−0.0540	0.0000	0.0000	−0.0449	0.0000	0.0000	−0.0535	0.0000	0.0000
	0.0000	−0.0623	0.0000	0.0000	−0.0485	0.0000	0.0000	−0.0546	0.0000
	0.0000	0.0000	0.1047	0.0000	0.0000	0.1072	0.0000	0.0000	0.1102
25C	−0.0148	0.0000	0.0000	−0.1092	0.0000	0.0000	−0.1054	0.0000	0.0000
	0.0000	−0.0328	0.0000	0.0000	−0.1132	0.0000	0.0000	−0.1082	0.0000
	0.0000	0.0000	0.2647	0.0000	0.0000	0.1781	0.0000	0.0000	0.2044
26C	0.2080	0.0000	0.0000	0.1488	0.0000	0.0000	−0.1792	0.0000	0.0000
	0.0000	−0.2726	0.0000	0.0000	−0.3327	0.0000	0.0000	−0.1836	0.0000
	0.0000	0.0000	−0.2930	0.0000	0.0000	−0.3711	0.0000	0.0000	0.3137
27C	−0.0266	0.0000	0.0000	−0.0023	0.0000	0.0000	−0.0951	0.0000	0.0000
	0.0000	−0.0418	0.0000	0.0000	0.0284	0.0000	0.0000	−0.0974	0.0000
	0.0000	0.0000	0.2920	0.0000	0.0000	0.3380	0.0000	0.0000	0.2201
28C	−0.0598	0.0000	0.0000	−0.0672	0.0000	0.0000	−0.0547	0.0000	0.0000
	0.0000	−0.0685	0.0000	0.0000	−0.0854	0.0000	0.0000	−0.0554	0.0000
	0.0000	0.0000	0.1133	0.0000	0.0000	0.1086	0.0000	0.0000	0.1124
29C	−0.0209	0.0000	0.0000	0.0009	0.0000	0.0000	−0.0398	0.0000	0.0000
	0.0000	−0.0384	0.0000	0.0000	−0.0323	0.0000	0.0000	−0.0423	0.0000
	0.0000	0.0000	0.0907	0.0000	0.0000	0.0981	0.0000	0.0000	0.0862
30C	−0.0547	0.0000	0.0000	−0.0619	0.0000	0.0000	−0.0540	0.0000	0.0000
	0.0000	−0.0632	0.0000	0.0000	−0.0803	0.0000	0.0000	−0.0547	0.0000
	0.0000	0.0000	0.1045	0.0000	0.0000	0.0986	0.0000	0.0000	0.1084
31C	−0.0138	0.0000	0.0000	0.0083	0.0000	0.0000	−0.0980	0.0000	0.0000
	0.0000	−0.0329	0.0000	0.0000	0.0402	0.0000	0.0000	−0.1032	0.0000
	0.0000	0.0000	0.2635	0.0000	0.0000	0.3087	0.0000	0.0000	0.2014
32C	0.1956	0.0000	0.0000	0.1655	0.0000	0.0000	−0.1113	0.0000	0.0000
	0.0000	−0.2763	0.0000	0.0000	−0.3040	0.0000	0.0000	−0.1237	0.0000
	0.0000	0.0000	−0.2947	0.0000	0.0000	−0.3356	0.0000	0.0000	0.3711
33C	−0.0057	0.0000	0.0000	0.0048	0.0000	0.0000	−0.1385	0.0000	0.0000
	0.0000	−0.0225	0.0000	0.0000	0.0314	0.0000	0.0000	−0.1465	0.0000
	0.0000	0.0000	0.2799	0.0000	0.0000	0.3083	0.0000	0.0000	0.1763
34C	−0.0524	0.0000	0.0000	−0.0583	0.0000	0.0000	−0.0494	0.0000	0.0000
	0.0000	−0.0596	0.0000	0.0000	−0.0735	0.0000	0.0000	−0.0536	0.0000
	0.0000	0.0000	0.1081	0.0000	0.0000	0.1024	0.0000	0.0000	0.1121
35C	−0.0239	0.0000	0.0000	−0.0054	0.0000	0.0000	−0.0452	0.0000	0.0000
	0.0000	−0.0386	0.0000	0.0000	−0.0328	0.0000	0.0000	−0.0524	0.0000
	0.0000	0.0000	0.0882	0.0000	0.0000	0.0943	0.0000	0.0000	0.0824
36C	−0.0552	0.0000	0.0000	−0.0636	0.0000	0.0000	−0.0487	0.0000	0.0000
	0.0000	−0.0624	0.0000	0.0000	−0.0794	0.0000	0.0000	−0.0524	0.0000
	0.0000	0.0000	0.1112	0.0000	0.0000	0.1025	0.0000	0.0000	0.1083
37C	−0.0022	0.0000	0.0000	0.0089	0.0000	0.0000	−0.1298	0.0000	0.0000
	0.0000	0.0162	0.0000	0.0000	−0.0187	0.0000	0.0000	−0.1357	0.0000
	0.0000	0.0000	0.3026	0.0000	0.0000	0.2985	0.0000	0.0000	0.1693
38C	0.2002	0.0000	0.0000	−0.1918	0.0000	0.0000	−0.1243	0.0000	0.0000
	0.0000	−0.2829	0.0000	0.0000	−0.1986	0.0000	0.0000	−0.1375	0.0000
	0.0000	0.0000	−0.2973	0.0000	0.0000	0.2890	0.0000	0.0000	0.3577
39C	−0.0053	0.0000	0.0000	−0.0966	0.0000	0.0000	−0.1459	0.0000	0.0000
	0.0000	−0.0150	0.0000	0.0000	−0.0992	0.0000	0.0000	−0.1506	0.0000
	0.0000	0.0000	0.3235	0.0000	0.0000	0.2363	0.0000	0.0000	0.1673
40C	−0.0540	0.0000	0.0000	−0.0498	0.0000	0.0000	−0.0530	0.0000	0.0000
	0.0000	−0.0591	0.0000	0.0000	−0.0526	0.0000	0.0000	−0.0562	0.0000
	0.0000	0.0000	0.1209	0.0000	0.0000	0.1204	0.0000	0.0000	0.1094
41C	−0.0322	0.0000	0.0000	−0.0447	0.0000	0.0000	−0.0444	0.0000	0.0000
	0.0000	−0.0408	0.0000	0.0000	−0.0490	0.0000	0.0000	−0.0502	0.0000
	0.0000	0.0000	0.0888	0.0000	0.0000	0.0840	0.0000	0.0000	0.0836
42C	−0.0516	0.0000	0.0000	−0.0482	0.0000	0.0000	−0.0490	0.0000	0.0000
	0.0000	−0.0567	0.0000	0.0000	−0.0498	0.0000	0.0000	−0.0528	0.0000
	0.0000	0.0000	0.1086	0.0000	0.0000	0.1079	0.0000	0.0000	0.1085
43C	−0.0044	0.0000	0.0000	−0.0864	0.0000	0.0000	−0.1379	0.0000	0.0000
	0.0000	−0.0183	0.0000	0.0000	−0.0892	0.0000	0.0000	−0.1433	0.0000
	0.0000	0.0000	0.2816	0.0000	0.0000	0.2026	0.0000	0.0000	0.1630
44C	−0.0278	0.0000	0.0000	−0.0223	0.0000	0.0000	−0.0241	0.0000	0.0000
	0.0000	−0.0281	0.0000	0.0000	−0.0226	0.0000	0.0000	−0.0243	0.0000
	0.0000	0.0000	0.0456	0.0000	0.0000	0.0504	0.0000	0.0000	0.0499
45C	−0.0283	0.0000	0.0000	−0.0322	0.0000	0.0000	−0.0251	0.0000	0.0000
	0.0000	−0.0287	0.0000	0.0000	−0.0332	0.0000	0.0000	−0.0252	0.0000
	0.0000	0.0000	0.0464	0.0000	0.0000	0.0427	0.0000	0.0000	0.0494
46C	−0.0273	0.0000	0.0000	−0.0305	0.0000	0.0000	−0.0229	0.0000	0.0000
	0.0000	−0.0270	0.0000	0.0000	−0.0313	0.0000	0.0000	−0.0231	0.0000
	0.0000	0.0000	0.0462	0.0000	0.0000	0.0427	0.0000	0.0000	0.0511
47C	−0.0268	0.0000	0.0000	−0.0237	0.0000	0.0000	−0.0233	0.0000	0.0000
	0.0000	−0.0270	0.0000	0.0000	−0.0239	0.0000	0.0000	−0.0235	0.0000
	0.0000	0.0000	0.0483	0.0000	0.0000	0.0509	0.0000	0.0000	0.0509

Table S11: ^{1}H hyperfine tensors $\boldsymbol{A}$ (MHz) calculated at the DLPNO-CCSD/cc-pwCVTZ(Fe,Cl)/EPR-II(O,N,C,H) level of theory for the Fe(III)Cl@TCPP, Fe(III)OH@TCPP, and Fe(II)@TCPP models.

	Fe(III)Cl@TCPP			Fe(III)OH@TCPP			Fe(II)@TCPP		
Atom	A_{xx}	A_{yy}	A_{zz}	A_{xx}	A_{yy}	A_{zz}	A_{xx}	A_{yy}	A_{zz}
60H	−0.4357	0.0000	0.0000	−0.4420	0.0000	0.0000	−0.4299	0.0000	0.0000
	0.0000	−0.4588	0.0000	0.0000	−0.4630	0.0000	0.0000	−0.4552	0.0000
	0.0000	0.0000	1.2301	0.0000	0.0000	1.2088	0.0000	0.0000	1.2229
61H	−0.4127	0.0000	0.0000	−0.4944	0.0000	0.0000	−0.4936	0.0000	0.0000
	0.0000	−0.4444	0.0000	0.0000	−0.5051	0.0000	0.0000	−0.4965	0.0000
	0.0000	0.0000	1.2575	0.0000	0.0000	1.1883	0.0000	0.0000	1.1980
62H	−0.4033	0.0000	0.0000	−0.3454	0.0000	0.0000	−0.3718	0.0000	0.0000
	0.0000	−0.4331	0.0000	0.0000	−0.3795	0.0000	0.0000	−0.4148	0.0000
	0.0000	0.0000	1.2146	0.0000	0.0000	1.2464	0.0000	0.0000	1.2464
63H	−0.4067	0.0000	0.0000	−0.4928	0.0000	0.0000	−0.4879	0.0000	0.0000
	0.0000	−0.4349	0.0000	0.0000	−0.5058	0.0000	0.0000	−0.4912	0.0000
	0.0000	0.0000	1.2241	0.0000	0.0000	1.1602	0.0000	0.0000	1.1933
64H	−0.4550	0.0000	0.0000	−0.4199	0.0000	0.0000	−0.5471	0.0000	0.0000
	0.0000	−0.4758	0.0000	0.0000	−0.4537	0.0000	0.0000	−0.5604	0.0000
	0.0000	0.0000	1.2298	0.0000	0.0000	1.2397	0.0000	0.0000	1.1570
65H	−0.4047	0.0000	0.0000	−0.4293	0.0000	0.0000	−0.3695	0.0000	0.0000
	0.0000	−0.4357	0.0000	0.0000	−0.4551	0.0000	0.0000	−0.4117	0.0000
	0.0000	0.0000	1.2572	0.0000	0.0000	1.2226	0.0000	0.0000	1.2602
66H	−0.4009	0.0000	0.0000	−0.4845	0.0000	0.0000	−0.3929	0.0000	0.0000
	0.0000	−0.4327	0.0000	0.0000	−0.5002	0.0000	0.0000	−0.4231	0.0000
	0.0000	0.0000	1.2196	0.0000	0.0000	1.1522	0.0000	0.0000	1.2440
67H	−0.4172	0.0000	0.0000	−0.3961	0.0000	0.0000	−0.4933	0.0000	0.0000
	0.0000	−0.4474	0.0000	0.0000	−0.4293	0.0000	0.0000	−0.4976	0.0000
	0.0000	0.0000	1.2189	0.0000	0.0000	1.2234	0.0000	0.0000	1.1948
68H	−0.4115	0.0000	0.0000	−0.4013	0.0000	0.0000	−0.4374	0.0000	0.0000
	0.0000	−0.4231	0.0000	0.0000	−0.4112	0.0000	0.0000	−0.4496	0.0000
	0.0000	0.0000	0.8186	0.0000	0.0000	0.8162	0.0000	0.0000	0.8997
69H	−0.1487	0.0000	0.0000	−0.1515	0.0000	0.0000	−0.1589	0.0000	0.0000
	0.0000	−0.1493	0.0000	0.0000	−0.1531	0.0000	0.0000	−0.1607	0.0000
	0.0000	0.0000	0.3077	0.0000	0.0000	0.3013	0.0000	0.0000	0.3130
70H	−0.4227	0.0000	0.0000	−0.4095	0.0000	0.0000	−0.4706	0.0000	0.0000
	0.0000	−0.4348	0.0000	0.0000	−0.4206	0.0000	0.0000	−0.4772	0.0000
	0.0000	0.0000	0.8374	0.0000	0.0000	0.8368	0.0000	0.0000	0.9454
71H	−0.1479	0.0000	0.0000	−0.1527	0.0000	0.0000	−0.1614	0.0000	0.0000
	0.0000	−0.1488	0.0000	0.0000	−0.1546	0.0000	0.0000	−0.1626	0.0000
	0.0000	0.0000	0.3098	0.0000	0.0000	0.3003	0.0000	0.0000	0.3235
72H	−0.1584	0.0000	0.0000	−0.1656	0.0000	0.0000	−0.1593	0.0000	0.0000
	0.0000	−0.1594	0.0000	0.0000	−0.1666	0.0000	0.0000	−0.1603	0.0000
	0.0000	0.0000	0.3325	0.0000	0.0000	0.3241	0.0000	0.0000	0.3164
73H	−0.4872	0.0000	0.0000	−0.4774	0.0000	0.0000	−0.4530	0.0000	0.0000
	0.0000	−0.4925	0.0000	0.0000	−0.4828	0.0000	0.0000	−0.4580	0.0000
	0.0000	0.0000	0.9603	0.0000	0.0000	0.9784	0.0000	0.0000	0.9112
74H	−0.5252	0.0000	0.0000	−0.5408	0.0000	0.0000	−0.4843	0.0000	0.0000
	0.0000	−0.5335	0.0000	0.0000	−0.5539	0.0000	0.0000	−0.4892	0.0000
	0.0000	0.0000	1.0418	0.0000	0.0000	1.0470	0.0000	0.0000	0.9720
75H	−0.1659	0.0000	0.0000	−0.1621	0.0000	0.0000	−0.1635	0.0000	0.0000
	0.0000	−0.1673	0.0000	0.0000	−0.1643	0.0000	0.0000	−0.1642	0.0000
	0.0000	0.0000	0.3488	0.0000	0.0000	0.3587	0.0000	0.0000	0.3293
76H	−0.4111	0.0000	0.0000	−0.4173	0.0000	0.0000	−0.4437	0.0000	0.0000
	0.0000	−0.4237	0.0000	0.0000	−0.4339	0.0000	0.0000	−0.4521	0.0000
	0.0000	0.0000	0.8143	0.0000	0.0000	0.8051	0.0000	0.0000	0.8930
77H	−0.1471	0.0000	0.0000	−0.1417	0.0000	0.0000	−0.1567	0.0000	0.0000
	0.0000	−0.1481	0.0000	0.0000	−0.1441	0.0000	0.0000	−0.1577	0.0000
	0.0000	0.0000	0.3079	0.0000	0.0000	0.3135	0.0000	0.0000	0.3167
78H	−0.4314	0.0000	0.0000	−0.4345	0.0000	0.0000	−0.4804	0.0000	0.0000
	0.0000	−0.4431	0.0000	0.0000	−0.4498	0.0000	0.0000	−0.4905	0.0000
	0.0000	0.0000	0.8554	0.0000	0.0000	0.8481	0.0000	0.0000	0.9797
79H	−0.1495	0.0000	0.0000	−0.1456	0.0000	0.0000	−0.1659	0.0000	0.0000
	0.0000	−0.1503	0.0000	0.0000	−0.1473	0.0000	0.0000	−0.1675	0.0000
	0.0000	0.0000	0.3129	0.0000	0.0000	0.3162	0.0000	0.0000	0.3253
80H	−0.4794	0.0000	0.0000	−0.4867	0.0000	0.0000	−0.4367	0.0000	0.0000
	0.0000	−0.4876	0.0000	0.0000	−0.4960	0.0000	0.0000	−0.4458	0.0000
	0.0000	0.0000	0.9500	0.0000	0.0000	0.9402	0.0000	0.0000	0.8877
81H	−0.1581	0.0000	0.0000	−0.1534	0.0000	0.0000	−0.1580	0.0000	0.0000
	0.0000	−0.1592	0.0000	0.0000	−0.1548	0.0000	0.0000	−0.1599	0.0000
	0.0000	0.0000	0.3303	0.0000	0.0000	0.3352	0.0000	0.0000	0.3113
82H	−0.5262	0.0000	0.0000	−0.5273	0.0000	0.0000	−0.4764	0.0000	0.0000
	0.0000	−0.5389	0.0000	0.0000	−0.5336	0.0000	0.0000	−0.4881	0.0000
	0.0000	0.0000	1.0522	0.0000	0.0000	1.0684	0.0000	0.0000	0.9743
83H	−0.1680	0.0000	0.0000	−0.1738	0.0000	0.0000	−0.1652	0.0000	0.0000
	0.0000	−0.1695	0.0000	0.0000	−0.1744	0.0000	0.0000	−0.1670	0.0000
	0.0000	0.0000	0.3499	0.0000	0.0000	0.3443	0.0000	0.0000	0.3261

Table S12: ^{1}H hyperfine tensors $\boldsymbol{A}$ (MHz) calculated at the DLPNO-CCSD/cc-pwCVTZ(Fe,Cl)/EPR-II(O,N,C,H) level of theory for the Fe(II)(py-NMe-PiPr$_2$)Cl$_2$ catalyst.

	Fe(II)(py-NMe-PiPr$_2$)Cl$_2$		
Atom	A_{xx}	A_{yy}	A_{zz}
11H	-0.3661	0.0000	0.0000
	0.0000	-0.3852	0.0000
	0.0000	0.0000	1.2475
12H	-0.4225	0.0000	0.0000
	0.0000	-0.4412	0.0000
	0.0000	0.0000	0.7036
13H	-0.4903	0.0000	0.0000
	0.0000	-0.5048	0.0000
	0.0000	0.0000	1.2330
14H	-2.5334	0.0000	0.0000
	0.0000	-2.5731	0.0000
	0.0000	0.0000	6.2077
16H	-0.5536	0.0000	0.0000
	0.0000	-0.5676	0.0000
	0.0000	0.0000	1.1233
17H	-0.5920	0.0000	0.0000
	0.0000	-0.6015	0.0000
	0.0000	0.0000	1.3370
18H	-0.5602	0.0000	0.0000
	0.0000	-0.5697	0.0000
	0.0000	0.0000	1.0931
21H	-0.6469	0.0000	0.0000
	0.0000	-0.6696	0.0000
	0.0000	0.0000	1.2701
22H	-1.0772	0.0000	0.0000
	0.0000	-1.0982	0.0000
	0.0000	0.0000	2.1896
23H	-1.9000	0.0000	0.0000
	0.0000	-1.9977	0.0000
	0.0000	0.0000	3.8967
25H	-0.6189	0.0000	0.0000
	0.0000	-0.6474	0.0000
	0.0000	0.0000	1.1903
26H	-0.8305	0.0000	0.0000
	0.0000	-0.8586	0.0000
	0.0000	0.0000	1.6884
27H	-1.6612	0.0000	0.0000
	0.0000	-1.7570	0.0000
	0.0000	0.0000	3.4639
28H	-0.2033	0.0000	0.0000
	0.0000	-0.2880	0.0000
	0.0000	0.0000	2.1805
31H	-0.6866	0.0000	0.0000
	0.0000	-0.7178	0.0000
	0.0000	0.0000	1.2236
32H	-1.0642	0.0000	0.0000
	0.0000	-1.0982	0.0000
	0.0000	0.0000	2.1670
33H	-1.9050	0.0000	0.0000
	0.0000	-2.0167	0.0000
	0.0000	0.0000	3.9777
35H	-0.5957	0.0000	0.0000
	0.0000	-0.6179	0.0000
	0.0000	0.0000	1.1674
36H	-0.7841	0.0000	0.0000
	0.0000	-0.7990	0.0000
	0.0000	0.0000	1.5685
37H	-1.5929	0.0000	0.0000
	0.0000	-1.6404	0.0000
	0.0000	0.0000	3.2043
38H	-0.1016	0.0000	0.0000
	0.0000	-0.1937	0.0000
	0.0000	0.0000	2.2793

Table S13: Cartesian coordinates of CH_4, $[Fe(NH_3)_6]^{3+}$, and TCPP models.

CH_4 reference			
	x (Å)	y (Å)	z (Å)
C	10.676062	10.046009	−0.000002
H	11.490663	9.793099	0.677904
H	11.004503	10.824338	−0.688093
H	10.385507	9.160794	−0.564886
H	9.823425	10.405870	0.575026

$[Fe(NH_3)_6]^{3+}$			
	x (Å)	y (Å)	z (Å)
Fe	10.442324	10.311322	0.052421
N	10.633322	12.495622	0.114315
N	12.628609	10.130107	0.005071
N	10.371373	8.119437	0.125642
N	8.251065	10.375761	−0.028877
N	10.413358	10.377691	−2.140476
N	10.358593	10.371134	2.245104
H	12.966241	9.417189	−0.644653
H	13.116671	10.982945	−0.275355
H	9.463582	7.733818	0.392808
H	10.591885	7.658723	−0.759489
H	11.329434	12.826381	0.785407
H	10.909343	12.922497	−0.772115
H	9.777004	12.987537	0.377032
H	7.847342	9.728435	−0.708855
H	11.313575	10.602268	−2.568677
H	10.131499	9.502795	−2.587082
H	11.253277	10.569331	2.697409
H	9.727708	11.086540	2.611842
H	9.768509	11.073211	−2.520946
H	10.041225	9.502974	2.680643
H	7.789095	10.141919	0.852066
H	13.053668	9.877065	0.899294
H	7.862694	11.285374	−0.285851
H	11.029277	7.711102	0.792552

TCPP			
	x (Å)	y (Å)	z (Å)
C	0.239009	0.950713	0.188247
C	−1.103647	0.868115	0.094524
C	−1.424107	−0.544145	0.075889
C	0.727707	−0.411259	0.243110
C	−3.095512	−2.372552	−0.034214
C	−4.383367	−2.914143	−0.311815
C	−4.302231	−4.271577	−0.210644
C	−2.965017	−4.607968	0.145872
C	−1.124539	−6.168405	0.714192
C	−0.691371	−7.484306	1.135484
C	0.622398	−7.377386	1.419576
C	0.986825	−6.001914	1.147472
C	2.298644	−5.521540	1.287927
C	−2.737910	−1.021544	−0.060235
C	2.093198	−0.716544	0.370927
C	−2.459113	−5.888745	0.379305
C	2.596653	−1.997076	0.614409
C	3.965996	−2.366166	0.745990
C	4.018310	−3.698690	1.031615
C	2.684588	−4.196539	1.063562
C	−3.837874	−0.045777	−0.269067
C	−3.866033	0.784673	−1.390439
C	−4.904287	1.682984	−1.585677
C	−5.946420	1.792503	−0.671203
C	−5.916523	0.968681	0.448770
C	−4.885190	0.062816	0.647420
C	3.078413	0.387128	0.247005
C	3.184465	1.110826	−0.941884
C	4.112486	2.132283	−1.074205
C	4.959361	2.477044	−0.026542
C	4.846520	1.764036	1.162055
C	3.927746	0.733936	1.298753
C	3.367262	−6.462656	1.709085
C	4.056321	−6.256717	2.905487
C	5.042045	−7.137556	3.322381
C	5.383966	−8.249171	2.560298
C	4.707374	−8.448010	1.361728
C	3.715641	−7.574231	0.940750
C	−3.412630	−7.021942	0.267273
C	−3.242022	−7.996644	−0.716687
C	−4.142064	−9.043471	−0.845073
C	−5.231307	−9.166661	0.010524
C	−5.391884	−8.204853	1.002047
C	−4.505223	−7.145281	1.126113
C	−7.098716	2.792251	−0.895077
C	5.989985	3.613046	−0.180077
C	6.472239	−9.231318	3.036850
C	−6.229280	−10.331616	−0.140399
N	−0.299337	−1.296322	0.191271
N	−2.276609	−3.431996	0.229067
N	−0.093885	−5.286809	0.741656
N	1.867297	−3.136411	0.798729
O	−7.034172	3.459958	−1.947046
O	−7.967684	2.821366	−0.000266
O	6.035211	4.147387	−1.306583
O	6.664056	3.870029	0.837997
O	7.001978	−8.954061	4.131995
O	6.700011	−10.194332	2.277221
O	−7.118564	−10.396128	0.732355
O	−6.036699	−11.078429	−1.121117
H	−5.244135	−2.320482	−0.572812
H	−5.084714	−4.994980	−0.371783
H	−1.821761	1.671857	0.056076
H	0.852973	1.835855	0.243323
H	4.790504	−1.682555	0.626820
H	4.893138	−4.307994	1.189995
H	1.290077	−8.139007	1.789827
H	−1.327405	−8.351186	1.222911
H	−1.283420	−3.361912	0.404667
H	0.862487	−3.193609	0.702249
H	−4.875934	−0.570608	1.529297
H	−6.730344	1.066992	1.158942
H	−4.942030	2.326152	−2.458029
H	−3.062677	0.709816	−2.116550
H	2.530517	0.852971	−1.768987
H	4.212370	2.693176	−1.996968
H	3.850021	0.190772	2.235596
H	5.504578	2.050252	1.975282
H	3.795374	−5.398528	3.517206
H	5.574379	−6.995393	4.256454
H	3.198391	−7.744626	0.001690
H	4.987929	−9.313726	0.771573
H	−2.395343	−7.915031	−1.391198
H	−4.031008	−9.796604	−1.617407
H	−4.646301	−6.402108	1.905031
H	−6.237132	−8.320234	1.671758

Table S14: Cartesian coordinates of Fe(III)Cl@TCPP, Fe(III)OH@TCPP, and Fe(II)@TCPP.

	Fe(III)Cl@TCPP				Fe(III)OH@TCPP				Fe(II)@TCPP		
	x (Å)	y (Å)	z (Å)		x (Å)	y (Å)	z (Å)		x (Å)	y (Å)	z (Å)
C	0.248445	0.917045	0.162678	C	0.242407	0.914514	0.163429	C	0.240669	0.954067	0.167656
C	−1.100726	0.833801	0.071563	C	−1.107991	0.832837	0.078077	C	−1.108984	0.869861	0.057422
C	−1.432834	−0.557731	0.041700	C	−1.442669	−0.559049	0.050449	C	−1.445474	−0.524654	0.053521
C	0.747436	−0.422803	0.207098	C	0.741232	−0.426527	0.206469	C	0.741881	−0.387561	0.246272
C	−3.052455	−2.403680	−0.053087	C	−3.066037	−2.409555	−0.028539	C	−3.083756	−2.390954	−0.012111
C	−4.364143	−2.920309	−0.298778	C	−4.379264	−2.928434	−0.273454	C	−4.388050	−2.917412	−0.295753
C	−4.284922	−4.268723	−0.197794	C	−4.296341	−4.277031	−0.179396	C	−4.307091	−4.266796	−0.181665
C	−2.927756	−4.584464	0.126342	C	−2.935552	−4.591233	0.139501	C	−2.955752	−4.578282	0.185667
C	−1.144022	−6.170237	0.709612	C	−1.141510	−6.183469	0.699851	C	−1.141043	−6.185495	0.728867
C	−0.702838	−7.460200	1.143812	C	−0.702367	−7.474230	1.137186	C	−0.689312	−7.486336	1.129915
C	0.615871	−7.349576	1.434399	C	0.615530	−7.363973	1.432604	C	0.635465	−7.381391	1.402594
C	0.992169	−5.996036	1.160483	C	0.994544	−6.010255	1.156375	C	1.011203	−6.019337	1.151395
C	2.289357	−5.503538	1.301438	C	2.291679	−5.513281	1.304358	C	2.310769	−5.510740	1.293363
C	−2.734197	−1.045496	−0.085635	C	−2.745579	−1.050011	−0.068711	C	−2.749080	−1.029377	−0.067523
C	2.099675	−0.741626	0.346377	C	2.094546	−0.749181	0.346011	C	2.096596	−0.718761	0.405129
C	−2.464193	−5.877783	0.366163	C	−2.464550	−5.886542	0.363331	C	−2.471264	−5.874629	0.411479
C	2.560609	−2.033480	0.599826	C	2.559373	−2.039903	0.609513	C	2.576029	−2.012373	0.659638
C	3.939257	−2.372974	0.776754	C	3.941186	−2.378064	0.781003	C	3.963008	−2.360687	0.775239
C	3.986330	−3.692885	1.077720	C	3.990610	−3.697513	1.082894	C	4.019091	−3.688468	1.048963
C	2.639071	−4.174141	1.062971	C	2.641607	−4.180090	1.074367	C	2.668219	−4.169456	1.083881
C	−3.835705	−0.069601	−0.277937	C	−3.847490	−0.075095	−0.266455	C	−3.852413	−0.059381	−0.282370
C	−3.879311	0.751135	−1.405615	C	−3.888617	0.743215	−1.396259	C	−3.884846	0.763463	−1.409375
C	−4.913110	1.657627	−1.585262	C	−4.924350	1.645448	−1.585503	C	−4.922924	1.661439	−1.606866
C	−5.933152	1.784400	−0.648493	C	−5.950479	1.771391	−0.655401	C	−5.960521	1.779157	−0.688317
C	−5.886826	0.969500	0.477521	C	−5.906782	0.960568	0.473682	C	−5.926879	0.962515	0.436895
C	−4.860069	0.055279	0.661563	C	−4.877717	0.050589	0.666577	C	−4.895904	0.056689	0.637581
C	3.085796	0.362185	0.247094	C	3.080678	0.353711	0.230329	C	3.085972	0.382010	0.291533
C	3.225075	1.077272	−0.942947	C	3.207168	1.062868	−0.965003	C	3.205790	1.105971	−0.896064
C	4.145453	2.108796	−1.048446	C	4.128252	2.091563	−1.089371	C	4.132838	2.129487	−1.017425
C	4.948930	2.470663	0.027236	C	4.946977	2.457599	−0.026790	C	4.965434	2.476622	0.040800
C	4.803111	1.763527	1.215779	C	4.813927	1.757979	1.167716	C	4.840539	1.762100	1.227364
C	3.891551	0.724215	1.327202	C	3.901284	0.721559	1.297242	C	3.922386	0.729984	1.353146
C	3.358498	−6.441104	1.727417	C	3.359307	−6.452127	1.729689	C	3.384470	−6.450565	1.702143
C	4.001467	−6.274646	2.954179	C	4.025507	−6.268972	2.941962	C	4.079537	−6.250731	2.896128
C	4.994343	−7.152682	3.361218	C	5.012155	−7.152091	3.352579	C	5.065819	−7.134880	3.304495
C	5.391279	−8.213918	2.555311	C	5.377400	−8.239916	2.567253	C	5.401229	−8.245216	2.537476
C	4.756562	−8.373030	1.328392	C	4.721174	−8.416034	1.354070	C	4.719346	−8.437688	1.340923
C	3.752624	−7.507679	0.919833	C	3.726747	−7.542302	0.940348	C	3.728131	−7.559694	0.927787
C	−3.426068	−7.004674	0.274843	C	−3.423113	−7.015834	0.261592	C	−3.430521	−7.003422	0.304626
C	−3.282360	−7.973776	−0.718074	C	−3.271506	−7.979968	−0.735347	C	−3.271063	−7.975869	−0.683583
C	−4.187710	−9.018555	−0.825463	C	−4.172942	−9.026951	−0.854100	C	−4.173399	−9.021258	−0.806494
C	−5.252590	−9.143609	0.059918	C	−5.242832	−9.160103	0.024029	C	−5.254153	−9.145674	0.059550
C	−5.384674	−8.186346	1.060025	C	−5.382847	−8.208736	1.028756	C	−5.404169	−8.185833	1.054711
C	−4.492835	−7.129069	1.164799	C	−4.494726	−7.149408	1.144548	C	−4.514943	−7.127646	1.173437
C	−7.079863	2.794632	−0.855798	C	−7.099897	2.775965	−0.873751	C	−7.110933	2.780743	−0.913727
C	5.968370	3.620801	−0.095905	C	5.968500	3.603253	−0.170557	C	5.992786	3.617178	−0.100257
C	6.499450	−9.184641	3.009194	C	6.471335	−9.221707	3.030860	C	6.487317	−9.233363	3.006525
C	−6.257203	−10.305920	−0.069330	C	−6.243126	−10.324571	−0.117362	C	−6.253959	−10.309884	−0.085116
N	−0.293286	−1.307514	0.148683	N	−0.302265	−1.307488	0.149699	N	−0.303488	−1.262826	0.184082
N	−2.185682	−3.434875	0.190685	N	−2.200108	−3.439807	0.210669	N	−2.234276	−3.421530	0.270240
N	−0.100171	−5.288177	0.739851	N	−0.095644	−5.306281	0.729157	N	−0.090661	−5.314644	0.757033
N	1.783561	−3.150699	0.756940	N	1.787465	−3.156671	0.775223	N	1.815194	−3.132918	0.837826
O	−7.024152	3.460546	−1.909129	O	−7.045048	3.431957	−1.933565	O	−7.049363	3.440676	−1.970880
O	−7.934735	2.832047	0.051957	O	−7.956200	2.819854	0.032361	O	−7.975318	2.819537	−0.014920
O	6.030571	4.163451	−1.217298	O	6.023127	4.134146	−1.298195	O	6.044287	4.156210	−1.224346
O	6.616075	3.879012	0.938661	O	6.625917	3.870417	0.855618	O	6.657870	3.873313	0.923849
O	6.960418	−8.978565	4.150113	O	6.972892	−8.978798	4.147185	O	7.032127	−8.954560	4.093797
O	6.810203	−10.067044	2.184258	O	6.731436	−10.149939	2.239130	O	6.698088	−10.202741	2.249952
O	−7.131331	−10.363938	0.818918	O	−7.120340	−10.392325	0.767289	O	−7.137276	−10.374766	0.793602
O	−6.083622	−11.056435	−1.050408	O	−6.064103	−11.067570	−1.103394	O	−6.068379	−11.056280	−1.067586
H	−5.225725	−2.316777	−0.534636	H	−5.243703	−2.327069	−0.504779	H	−5.245509	−2.324574	−0.571676
H	−5.068515	−4.996828	−0.332219	H	−5.079287	−5.005494	−0.316137	H	−5.085423	−4.995070	−0.345167
H	−1.819978	1.636213	0.040678	H	−1.825485	1.637173	0.051789	H	−1.822027	1.676688	−0.000254
H	0.862748	1.801056	0.222597	H	0.856515	1.798854	0.222422	H	0.849230	1.842933	0.217602
H	4.759198	−1.680336	0.676867	H	4.761348	−1.686421	0.675566	H	4.784581	−1.674280	0.646449
H	4.851978	−4.304108	1.276632	H	4.858707	−4.307229	1.275791	H	4.895108	−4.301472	1.189816
H	1.284753	−8.106480	1.811285	H	1.280089	−8.121181	1.816373	H	1.303058	−8.150539	1.756446
H	−1.339567	−8.325827	1.233291	H	−1.339702	−8.339225	1.229080	H	−1.319179	−8.357710	1.214434
H	−4.611598	−6.387698	1.949145	H	−4.619867	−6.413342	1.932846	H	−4.648736	−6.384891	1.953940
H	−6.212356	−8.302644	1.751165	H	−6.213728	−8.331560	1.714922	H	−6.243663	−8.301125	1.731639
H	−4.837427	−0.571894	1.547620	H	−4.858187	−0.573805	1.554619	H	−4.884042	−0.571800	1.522839
H	−6.684465	1.081103	1.203874	H	−6.708538	1.071689	1.195582	H	−6.737558	1.066303	1.149876
H	−4.964417	2.294248	−2.461637	H	−4.972973	2.278820	−2.464443	H	−4.964145	2.298045	−2.483880
H	−3.093757	0.660851	−2.149233	H	−3.097969	0.655370	−2.134990	H	−3.085689	0.681882	−2.139548
H	2.606237	0.803657	−1.791707	H	2.576105	0.787317	−1.804214	H	2.563869	0.846069	−1.732027
H	4.271659	2.665485	−1.970447	H	4.243783	2.642185	−2.016474	H	4.242819	2.690543	−1.938954
H	3.786228	0.185482	2.263853	H	3.806960	0.188986	2.238508	H	3.835962	0.184575	2.287817
H	5.429459	2.062090	2.049316	H	5.451346	2.059935	1.991583	H	5.488798	2.048694	2.048285
H	3.703784	−5.448917	3.593291	H	3.749003	−5.426281	3.568211	H	3.823297	−5.393917	3.511553
H	5.493124	−7.044218	4.318067	H	5.528735	−7.028881	4.298122	H	5.603724	−6.996518	4.235933
H	3.267571	−7.644101	−0.041719	H	3.225772	−7.692505	−0.010918	H	3.208420	−7.724425	−0.011045
H	5.082212	−9.199011	0.705588	H	5.021095	−9.262931	0.746523	H	4.995107	−9.301857	0.746218
H	−2.456827	−7.887456	−1.417674	H	−2.441411	−7.888328	−1.428911	H	−2.432007	−7.892899	−1.367548
H	−4.100141	−9.768400	−1.603894	H	−4.078068	−9.772182	−1.636162	H	−4.071123	−9.772111	−1.582275
Fe	−0.124014	−3.364280	−0.026164	Fe	−0.126712	−3.373956	−0.016820	Fe	−0.203249	−3.283437	0.510450
Cl	0.217049	−3.685505	−2.200201	O	0.146277	−3.588903	−1.817059				
				H	−0.134292	−2.843954	−2.353216				

Table S15: Cartesian coordinates of Fe(II)(py-NMe-PiPr$_2$)Cl$_2$ and TCPP linker (X-ray + opt. H).

	Fe(II)(py-NMe-PiPr$_2$)Cl$_2$				TCPP linker X-ray + opt. H		
Atom	x (Å)	y (Å)	z (Å)	Atom	x (Å)	y (Å)	z (Å)
Fe	5.918436	13.931969	8.074312	O	45.31363	12.15124	30.05655
Cl	4.412047	15.402638	7.379218	O	31.47574	25.98913	27.91694
Cl	5.822149	11.751791	8.476365	O	31.98437	26.49775	30.05655
P	7.536478	14.881036	9.576513	O	45.82225	25.98913	27.91694
N	8.885790	15.021302	8.515279	O	31.47574	12.65987	27.91694
C	8.735235	14.769651	7.169439	O	31.98437	12.15124	30.05655
N	7.561095	14.279798	6.748603	O	45.82225	12.65987	27.91694
C	7.368505	14.043573	5.451583	O	45.31363	26.49775	30.05655
C	8.329245	14.269901	4.491507	N	36.59519	19.32450	28.97399
C	9.551710	14.768487	4.918212	N	38.64900	17.27069	28.99950
C	9.767236	15.022843	6.255546	N	38.64900	21.37831	28.99950
H	10.714799	15.421911	6.584344	N	40.70281	19.32450	28.97399
H	10.342775	14.967490	4.204259	C	42.87797	19.99583	29.08569
H	8.123688	14.066582	3.449827	C	41.08350	21.75900	28.98675
H	6.387826	13.662112	5.189739	C	41.08350	16.89000	28.98675
C	10.145605	15.557842	8.979617	C	43.36263	24.54830	30.11723
H	10.341817	16.554850	8.570321	C	44.10469	13.86881	28.98675
H	10.125210	15.637620	10.064797	C	33.42520	14.61087	27.85627
H	10.978288	14.900957	8.712806	C	42.14519	15.82831	28.98675
C	8.174955	13.865090	10.976706	C	37.54480	22.18568	28.95854
C	6.998086	13.322210	11.786537	C	37.97767	15.09553	28.88781
H	7.378591	12.756008	12.640486	C	32.13007	12.80557	28.98675
H	6.344357	14.104058	12.171931	C	34.40418	23.05065	27.85820
H	6.396342	12.649088	11.171809	C	42.89382	15.59835	27.85820
C	9.014848	12.706204	10.451377	C	32.13007	25.84343	28.98675
H	9.316192	12.070484	11.287828	C	42.37515	15.07968	30.11530
H	9.918508	13.037981	9.939693	C	42.14519	22.82069	28.98675
H	8.431988	12.092514	9.759751	C	33.93537	14.10070	30.11723
H	8.788949	14.511158	11.616971	C	35.15281	22.82069	28.98675
C	7.346182	16.605624	10.195769	C	34.42003	19.99583	29.08569
C	7.151471	17.538103	9.005290	C	35.78781	20.42870	29.01496
H	7.047282	18.566308	9.361211	C	39.32033	23.55347	28.88781
H	7.992532	17.509505	8.310149	C	44.10469	24.78019	28.98675
H	6.246074	17.277872	8.450844	C	34.92285	23.56932	30.11530
C	6.168038	16.711048	11.157998	C	33.19331	13.86881	28.98675
H	6.001687	17.760563	11.413313	C	43.87280	24.03813	27.85627
H	6.338069	16.171031	12.089491	C	39.32033	15.09553	28.88781
H	5.252119	16.333222	10.696283	C	35.78781	18.22030	29.01496
H	8.267395	16.874776	10.727447	C	45.16792	25.84343	28.98675
				C	43.87280	14.61087	27.85627
				C	33.93537	24.54830	30.11723
				C	45.16792	12.80557	28.98675
				C	42.37515	23.56932	30.11530
				C	41.51018	18.22030	29.01496
				C	42.89382	23.05065	27.85820
				C	33.42520	24.03813	27.85627
				C	34.42003	18.65317	29.08569
				C	36.21450	16.89000	28.98675
				C	39.75320	22.18568	28.95854
				C	35.15281	15.82831	28.98675
				C	34.40418	15.59835	27.85820
				C	34.92285	15.07968	30.11530
				C	36.21450	21.75900	28.98675
				C	41.51018	20.42870	29.01496
				C	33.19331	24.78019	28.98675
				C	42.87797	18.65317	29.08569
				C	43.36263	14.10070	30.11723
				C	37.54480	16.46331	28.95854
				C	39.75320	16.46331	28.95854
				C	37.97767	23.55347	28.88781
				H	42.70723	22.45824	26.96766
				H	44.47841	14.39688	26.98334
				H	41.78003	15.26426	31.00425
				H	32.81958	24.25212	26.98334
				H	37.32311	14.23810	28.83499
				H	33.72275	13.49401	30.98970
				H	35.51797	15.26426	31.00425
				H	39.97489	14.23810	28.83499
				H	37.32311	24.41090	28.83499
				H	33.58489	20.67566	29.13415
				H	39.69300	19.32450	28.95713
				H	33.58489	17.97334	29.13415
				H	34.59077	16.19076	26.96766
				H	35.51797	23.38474	31.00425
				H	39.97489	24.41090	28.83499
				H	43.71311	20.67567	29.13415
				H	43.57525	13.49401	30.98970
				H	33.72275	25.15499	30.98970
				H	42.70723	16.19076	26.96766
				H	32.81958	14.39688	26.98334
				H	34.59077	22.45825	26.96766
				H	44.47842	24.25212	26.98334
				H	41.78003	23.38475	31.00425
				H	43.57525	25.15499	30.98970
				H	37.60500	19.32450	28.95715
				H	43.71311	17.97333	29.13415

Table S16: Calculated vibrational frequencies for CH_4, $[Fe(NH_3)_6]^{3+}$, and TCPP models.

CH_4 reference		$[Fe(NH_3)_6]^{3+}$		TCPP	
Freq. (cm^{-1})	Int. (10^5 cm/mol)	Freq. (cm^{-1})	Int. (10^5 cm/mol)	Freq. (cm^{-1})	Int. (10^5 cm/mol)
1326.08	16.13	61.62	0.00	9.80	0.48
1326.52	16.14	72.59	0.00	23.96	14.47
1326.62	16.14	105.14	0.01	25.22	9.51
1551.31	0.00	107.41	0.00	26.07	9.08
1551.43	0.00	109.16	0.01	28.20	9.40
3046.85	0.00	135.95	0.01	35.94	0.29
3168.50	19.40	141.01	0.01	36.30	0.38
3168.54	19.41	143.44	0.01	41.56	0.26
3168.67	19.40	150.96	0.05	43.78	0.25
		175.94	1.00	47.93	1.00
		177.81	1.01	51.56	0.25
		179.91	1.09	55.89	0.56
		195.02	0.01	60.05	0.69
		195.97	0.00	68.01	0.01
		206.56	0.00	77.49	0.27
		284.48	0.02	81.68	0.05
		285.19	0.01	84.89	0.09
		357.30	5.77	88.37	0.03
		357.76	5.90	95.26	0.13
		358.51	5.75	112.45	7.92
		373.11	0.00	122.98	14.65
		573.85	0.00	124.54	13.07
		574.94	0.01	126.00	1.87
		576.93	0.02	129.66	3.09
		610.78	0.00	140.77	0.59
		613.81	0.26	147.41	0.05
		614.72	0.15	154.73	4.03
		690.44	184.50	159.07	4.00
		692.13	163.51	161.82	0.08
		692.96	165.82	171.15	0.73
		702.93	21.19	171.76	3.15
		703.64	20.74	187.96	15.68
		707.30	0.84	189.59	20.10
		1365.79	3.01	204.51	0.07
		1365.97	2.21	214.65	2.06
		1374.13	217.44	224.60	0.09
		1376.55	220.05	234.41	0.08
		1377.31	218.69	263.84	0.02
		1413.05	0.01	283.96	0.32
		1634.85	135.92	291.69	2.31
		1645.75	0.04	296.77	0.01
		1646.63	0.05	308.48	0.02
		1653.26	0.03	314.83	1.00
		1656.19	47.28	322.12	0.00
		1656.31	46.24	334.78	0.51
		1661.60	60.94	337.51	0.05
		1662.19	54.50	340.85	2.53
		1666.50	0.14	352.58	2.05
		1674.98	36.11	356.07	13.60
		1675.27	27.80	368.99	12.85
		1678.13	0.94	397.09	10.67
		3426.38	11.36	397.54	13.77
		3426.47	10.42	427.43	0.48
		3426.61	158.87	429.08	1.74
		3427.72	180.72	430.34	3.32
		3427.95	182.07	430.85	0.13
		3436.69	0.01	433.19	2.06
		3506.78	10.90	442.34	0.16
		3506.87	11.80	452.09	0.07
		3508.61	0.83	466.56	27.94
		3508.98	108.42	477.18	38.08
		3510.26	65.44	482.17	0.69
		3510.35	48.82	494.63	10.86
		3510.49	171.00	500.98	3.26
		3510.57	104.31	504.37	0.96
		3510.60	33.20	507.12	0.24
		3511.79	152.33	521.78	1.27
		3511.89	157.21	528.90	1.48
		3512.04	2.56	537.35	0.52
				538.45	3.24
				553.24	0.04
				571.60	1.16
				578.07	2.84
				579.07	0.40
				587.44	0.50
				648.84	0.02
				649.52	0.02
				649.71	0.06
				650.57	0.01
				670.91	1.39
				686.69	0.40
				692.84	0.06
				694.22	0.65
				703.22	4.84
				703.91	5.05
				704.82	3.01

Table S16: Continuation.

CH_4 reference		$[Fe(NH_3)_6]^{3+}$		TCPP	
Freq. (cm^{-1})	Int. (10^5 cm/mol)	Freq. (cm^{-1})	Int. (10^5 cm/mol)	Freq. (cm^{-1})	Int. (10^5 cm/mol)
				711.93	0.06
				725.78	6.14
				728.09	21.95
				729.07	21.64
				731.60	0.42
				740.64	5.45
				756.04	10.03
				758.48	91.67
				767.87	9.28
				776.32	0.09
				794.58	6.52
				798.61	18.72
				799.81	26.40
				808.77	0.11
				822.09	5.83
				826.16	104.49
				826.47	82.48
				827.50	96.70
				827.86	175.09
				828.56	132.59
				834.26	47.09
				841.78	3.39
				845.73	0.02
				848.05	2.91
				852.16	6.69
				853.94	44.04
				868.60	2.50
				871.30	3.14
				872.12	3.19
				874.41	3.20
				892.47	0.45
				896.62	22.21
				897.64	36.54
				902.47	0.60
				904.88	12.11
				906.15	1.69
				907.08	2.25
				909.47	1.27
				940.39	0.21
				941.13	0.29
				942.34	0.28
				973.23	3.52
				986.27	0.47
				988.00	0.77
				989.04	0.82
				991.29	0.97
				997.77	3.73
				998.60	15.77
				1000.35	1.63
				1000.58	14.63
				1003.24	21.70
				1004.10	24.71
				1006.21	1.65
				1018.00	9.81
				1020.97	54.16
				1032.97	5.46
				1034.62	3.82
				1040.50	1.54
				1042.75	26.39
				1043.69	11.54
				1045.60	10.71
				1047.28	9.56
				1080.18	0.05
				1088.65	2.37
				1092.61	0.82
				1094.97	9.73
				1096.12	4.04
				1096.83	2.08
				1099.34	4.24
				1101.65	16.29
				1134.76	3.15
				1135.13	3.67
				1135.40	3.03
				1137.44	4.05
				1159.91	0.38
				1183.99	0.24
				1184.71	0.02
				1185.77	0.10
				1187.19	0.04
				1215.15	4.52
				1225.83	84.50
				1234.21	0.55
				1248.56	3.01
				1253.55	22.81
				1271.45	0.04
				1295.80	0.43
				1298.18	37.56

Table S16: Continuation.

CH_4 reference		$[Fe(NH_3)_6]^{3+}$		TCPP	
Freq. (cm^{-1})	Int. (10^5 cm/mol)	Freq. (cm^{-1})	Int. (10^5 cm/mol)	Freq. (cm^{-1})	Int. (10^5 cm/mol)
				1300.08	21.57
				1300.49	8.77
				1302.52	0.94
				1304.92	0.16
				1320.45	25.16
				1341.75	2.68
				1344.69	0.21
				1350.63	1.37
				1352.48	1.19
				1358.39	0.83
				1360.69	2.00
				1370.83	2.08
				1380.84	544.08
				1381.26	1297.39
				1381.76	769.38
				1384.15	40.77
				1393.26	27.57
				1401.32	17.88
				1411.29	0.95
				1414.08	17.92
				1426.23	1.33
				1427.89	3.80
				1428.62	16.91
				1429.93	11.33
				1430.26	7.18
				1452.06	13.40
				1497.65	0.04
				1512.00	0.02
				1523.49	136.33
				1527.09	28.90
				1532.62	0.25
				1533.69	3.57
				1535.95	21.20
				1539.36	3.97
				1557.16	0.21
				1568.85	14.79
				1585.56	20.06
				1604.56	12.23
				1607.01	0.40
				1610.67	0.46
				1613.52	43.60
				1616.77	5.79
				1619.61	0.18
				1621.50	28.27
				1622.25	25.81
				1659.08	0.22
				1659.67	0.31
				1660.03	0.63
				1660.46	0.26
				1718.90	245.18
				1719.57	85.09
				1719.92	540.00
				1720.38	724.74
				3171.75	10.54
				3172.37	10.78
				3172.89	11.43
				3173.48	10.57
				3174.69	9.79
				3174.81	9.55
				3176.35	9.96
				3176.57	9.12
				3200.70	10.79
				3200.92	2.96
				3200.98	16.52
				3201.10	29.85
				3202.30	2.75
				3202.41	1.46
				3202.52	2.97
				3202.91	3.28
				3258.10	0.93
				3258.31	1.90
				3271.53	0.01
				3271.62	0.01
				3277.34	10.36
				3277.63	4.12
				3287.48	0.26
				3287.67	1.80
				3560.60	70.13
				3601.40	0.56

Table S17: Calculated vibrational frequencies for Fe(III)Cl@TCPP, Fe(III)OH@TCPP, and Fe(II)@TCPP models.

Fe(III)Cl@TCPP		Fe(III)OH@TCPP		Fe(II)@TCPP	
Freq. (cm^{-1})	Int. (10^5 cm/mol)	Freq. (cm^{-1})	Int. (10^5 cm/mol)	Freq. (cm^{-1})	Int. (10^5 cm/mol)
7.27	1.03	9.77	1.22	10.5	0.76
19.57	6.91	21.69	15.06	22.2	16.76
22.78	15.32	25.18	1.76	25.9	12.87
25.36	9.94	26.58	12.02	27.2	6.87
28.82	2.40	28.67	10.18	30.1	10.10
30.46	7.90	30.50	4.11	37.1	0.46
31.86	2.41	37.77	0.01	39.8	0.21
40.82	0.62	40.93	0.24	43.2	0.07
42.97	1.11	43.57	0.58	47.0	0.11
44.63	0.41	48.07	0.11	48.0	0.45
49.86	0.17	52.97	0.77	56.2	0.45
58.00	0.48	61.43	0.02	62.9	0.40
62.61	0.17	69.24	1.18	68.1	0.21
68.43	0.20	76.38	1.05	82.3	0.26
74.50	0.38	78.84	0.10	84.7	0.38
76.50	0.11	80.84	0.38	88.5	1.96
79.94	0.12	85.33	6.63	92.0	0.15
82.29	0.06	91.19	1.38	93.2	0.67
85.65	0.03	95.18	4.77	102.1	0.13
95.69	7.86	100.09	1.84	105.1	0.91
99.27	0.20	101.22	3.25	123.3	10.97
104.20	0.47	123.64	3.38	124.7	9.68
123.55	9.37	123.87	9.26	127.0	5.69
124.88	9.61	126.35	5.41	132.6	5.67
127.14	1.55	129.82	2.09	138.3	3.91
131.23	5.02	132.41	11.06	149.1	0.01
134.39	6.52	135.18	15.33	157.8	0.93
149.17	0.03	149.04	0.07	161.9	0.02
156.35	2.15	156.20	1.95	174.8	5.54
160.14	0.06	160.13	0.06	176.5	15.37
181.77	26.15	182.60	22.66	181.6	20.86
183.84	24.07	186.41	29.57	183.2	13.45
195.78	3.16	196.76	0.59	190.9	10.14
197.12	0.43	197.84	0.17	202.0	0.50
199.31	0.61	201.84	8.08	209.1	0.35
203.83	0.10	203.18	0.10	225.0	1.75
223.51	3.33	225.73	8.63	234.4	0.08
238.25	1.29	242.17	0.59	237.9	0.13
243.62	0.21	243.13	3.08	254.7	0.15
254.32	0.32	254.82	0.57	257.5	0.06
257.26	0.54	257.22	1.20	269.4	0.33
258.75	0.53	258.85	1.62	309.5	0.16
272.96	0.08	274.55	1.68	317.0	0.79
297.48	0.01	297.43	0.04	319.3	0.76
315.05	0.56	313.93	1.24	330.1	0.24
319.46	0.15	319.47	0.33	331.7	0.38
320.57	0.68	320.07	0.60	338.9	0.64
322.22	0.16	322.19	0.11	341.6	0.08
333.78	0.22	334.72	0.63	343.8	0.43
336.55	0.59	335.21	0.20	354.2	0.21
340.89	0.05	339.38	0.03	374.7	0.04
347.75	0.07	347.12	0.26	379.0	12.38
363.04	39.92	374.45	1.91	379.3	12.65
377.85	24.18	379.94	17.29	422.5	13.85
380.48	17.45	380.96	16.65	423.6	13.62
381.10	15.99	415.15	12.82	431.3	1.41
415.67	15.19	418.56	8.80	433.7	3.94
419.23	9.98	425.99	0.20	435.5	5.92
426.13	0.28	427.59	2.63	439.5	2.69
427.71	4.86	428.05	3.75	442.7	0.25
428.37	7.43	429.09	9.34	451.5	0.10
437.61	2.33	440.08	1.27	466.4	0.13
440.79	1.51	454.57	0.21	484.7	38.59
456.19	0.27	465.97	0.02	485.0	38.86
466.89	0.23	484.37	40.75	496.6	5.54
485.06	40.03	486.05	39.63	499.7	5.50
486.99	39.14	496.54	8.30	504.4	4.74
497.80	8.46	501.18	1.66	505.5	3.50
501.49	1.77	504.69	5.56	507.6	0.39
504.85	5.74	505.00	4.03	524.2	0.92
505.11	4.06	506.84	0.41	541.6	0.21
506.85	0.12	519.70	1.49	545.6	0.22
521.87	1.32	539.31	0.02	559.9	0.01
540.94	0.08	541.97	0.20	580.1	0.09
545.94	0.25	556.37	0.01	582.8	0.47
558.76	0.01	575.73	0.06	585.3	0.42
576.63	0.05	580.01	2.38	590.7	3.41
581.11	0.93	581.95	0.88	648.9	0.03
582.89	0.92	589.91	9.07	650.6	0.07
591.81	4.75	610.52	169.12	651.0	0.07
648.41	0.02	649.07	0.02	652.3	0.02
649.96	0.09	650.11	0.05	686.2	0.11
650.17	0.06	650.28	0.06	693.8	0.08

Table S17: Continuation.

Fe(III)Cl@TCPP		Fe(III)OH@TCPP		Fe(II)@TCPP	
Freq. (cm^{-1})	Int. (10^5 cm/mol)	Freq. (cm^{-1})	Int. (10^5 cm/mol)	Freq. (cm^{-1})	Int. (10^5 cm/mol)
651.82	0.14	651.64	0.21	694.2	0.11
682.55	0.01	663.28	3.64	699.6	0.93
691.02	0.08	681.94	0.14	705.2	2.63
693.54	0.11	691.94	0.03	706.5	4.44
694.70	0.19	692.56	0.24	708.9	2.55
699.73	0.32	693.74	0.10	718.1	0.50
705.81	4.01	699.25	0.02	728.6	13.01
706.56	3.43	705.70	3.54	731.1	17.30
717.95	0.08	706.04	3.76	733.0	21.51
726.02	9.45	717.72	0.05	735.0	2.07
728.25	24.06	725.25	9.52	756.4	50.90
730.12	15.02	727.53	23.75	764.9	6.28
733.41	7.25	728.76	24.90	772.6	8.07
754.14	48.89	730.37	0.86	778.1	4.01
762.41	5.65	750.20	63.77	798.8	10.55
768.54	9.00	760.72	5.98	804.5	17.62
776.10	1.76	762.60	5.47	806.5	15.91
796.74	8.67	775.14	0.26	812.7	0.77
800.90	20.26	795.24	8.42	825.6	12.00
803.25	20.84	800.10	22.69	826.7	81.34
810.36	1.75	800.64	18.64	828.1	235.67
826.38	36.56	808.29	0.24	828.7	244.49
827.40	144.96	822.63	0.43	830.3	34.48
827.87	179.10	826.48	97.30	831.7	19.15
827.92	242.81	827.72	253.47	836.5	45.64
829.72	24.60	828.16	204.15	847.5	0.10
833.75	6.54	829.94	34.76	853.1	0.38
837.84	36.20	830.92	25.31	854.1	0.42
847.68	0.05	832.92	12.86	863.7	22.14
851.42	29.87	838.06	64.17	865.7	4.42
852.86	2.41	847.76	0.01	875.2	2.47
854.54	0.43	852.56	0.15	877.4	2.77
863.96	1.21	853.66	0.10	880.8	4.14
869.87	2.05	863.66	1.42	884.0	3.82
870.92	4.72	869.04	0.11	896.6	0.86
871.74	2.51	869.71	8.02	901.3	26.86
883.30	2.97	870.07	6.95	902.1	21.09
894.83	0.55	870.68	0.19	908.5	1.05
897.05	20.10	895.04	0.51	912.8	0.01
898.80	16.53	897.15	23.41	914.3	10.63
904.50	4.57	897.40	22.68	916.6	3.15
910.35	6.01	901.55	0.08	920.5	8.78
912.07	0.87	908.54	10.79	941.0	0.25
912.21	0.06	911.81	0.41	942.3	0.15
919.02	7.06	912.15	0.06	943.3	0.17
942.97	0.29	912.92	0.23	988.2	0.69
943.77	0.26	941.25	0.11	990.1	1.58
945.55	0.40	942.04	0.09	992.5	1.18
971.66	2.44	942.40	0.57	993.8	1.16
987.89	0.38	942.59	0.32	1000.6	1.87
988.68	1.29	986.96	0.14	1006.8	1.45
988.70	0.09	987.25	0.66	1011.3	2.39
992.34	1.21	987.80	0.53	1017.4	4.30
1000.14	1.47	988.20	0.81	1021.5	9.63
1000.53	1.72	999.43	1.47	1035.1	96.48
1001.21	1.59	999.72	1.39	1035.4	107.58
1017.57	5.10	999.86	1.64	1035.7	6.47
1031.30	109.00	1000.55	1.58	1037.6	1.05
1033.03	1.23	1031.08	109.06	1040.1	1.06
1034.12	109.21	1032.33	8.47	1044.1	5.81
1039.23	1.24	1034.79	104.62	1044.4	4.75
1040.30	0.09	1038.65	1.20	1047.6	0.96
1042.84	1.75	1039.67	0.37	1052.3	3.35
1044.35	2.48	1041.53	2.87	1053.4	2.90
1046.54	0.51	1043.32	4.07	1056.5	2.53
1053.21	7.26	1044.66	0.52	1066.6	0.34
1053.81	6.33	1052.10	4.45	1086.8	3.03
1057.55	2.95	1053.38	2.42	1088.4	3.33
1067.92	0.07	1056.55	3.30	1095.5	0.25
1089.94	3.12	1067.70	0.06	1096.7	3.71
1090.88	4.67	1088.91	2.88	1098.8	3.01
1095.86	13.12	1089.99	4.22	1100.9	7.49
1096.49	0.26	1095.35	15.32	1103.3	12.25
1096.56	2.18	1095.86	0.04	1107.6	9.27
1097.57	2.99	1096.30	2.66	1134.6	2.98

Table S17: Continuation.

Fe(III)Cl@TCPP		Fe(III)OH@TCPP		Fe(II)@TCPP	
Freq. (cm^{-1})	Int. (10^5 cm/mol)	Freq. (cm^{-1})	Int. (10^5 cm/mol)	Freq. (cm^{-1})	Int. (10^5 cm/mol)
1101.62	4.79	1096.66	1.63	1135.0	3.32
1104.89	12.77	1097.32	3.09	1140.1	3.87
1134.32	2.72	1101.90	13.23	1142.8	4.24
1134.91	3.49	1134.70	2.49	1187.1	0.19
1135.21	2.41	1135.03	4.20	1188.7	0.13
1140.17	3.24	1135.26	3.88	1188.9	0.51
1185.03	0.01	1135.39	1.99	1190.4	0.11
1185.36	0.00	1183.75	0.05	1204.5	0.25
1185.62	0.01	1184.92	0.00	1241.5	42.82
1189.28	0.18	1185.36	0.01	1241.9	42.62
1210.05	0.37	1185.67	0.02	1273.6	16.86
1242.87	23.55	1208.16	0.38	1275.1	77.99
1243.09	23.01	1242.34	30.47	1275.9	64.77
1273.23	10.89	1243.13	31.90	1291.3	0.05
1273.91	46.57	1272.67	38.36	1303.2	0.03
1274.51	59.29	1273.96	25.60	1304.7	0.43
1281.86	0.04	1275.65	69.23	1305.4	0.12
1299.81	0.01	1286.33	0.13	1308.8	0.15
1301.23	0.01	1299.39	0.01	1313.4	0.77
1301.60	0.05	1299.78	0.15	1333.0	2.43
1306.72	0.49	1301.02	0.03	1337.7	1.79
1310.42	0.55	1302.37	0.03	1338.1	1.73
1329.99	1.28	1311.71	1.10	1347.7	0.44
1344.39	7.66	1333.97	2.04	1348.4	0.62
1346.14	15.76	1343.28	9.47	1351.7	0.11
1349.02	1.72	1344.44	4.74	1355.2	3.77
1349.66	1.09	1347.55	0.15	1368.6	0.07
1351.90	0.09	1348.19	0.03	1380.2	681.87
1354.56	4.42	1350.64	0.06	1381.0	1184.95
1374.23	0.11	1354.24	4.08	1381.4	666.00
1379.76	621.09	1373.05	0.50	1382.3	49.62
1380.43	1271.42	1379.86	608.13	1382.5	60.68
1380.87	713.31	1380.34	1260.15	1383.9	53.49
1383.19	46.55	1381.16	711.86	1403.2	0.00
1383.89	0.80	1383.20	5.85	1410.6	0.61
1385.39	2.76	1383.57	94.91	1426.9	2.88
1412.47	2.13	1384.25	5.65	1428.1	4.62
1416.50	1.02	1409.87	0.72	1429.1	7.95
1427.35	3.45	1414.45	0.84	1429.8	7.11
1427.68	1.97	1425.89	4.20	1481.7	0.90
1428.14	7.46	1427.22	3.00	1482.1	1.01
1428.40	7.96	1427.66	5.92	1498.3	0.04
1495.65	4.33	1428.16	7.97	1510.5	0.06
1496.12	3.36	1490.87	1.53	1527.1	96.05
1508.12	1.17	1493.15	1.64	1527.2	91.02
1521.88	0.06	1507.02	0.88	1535.7	2.02
1530.41	25.53	1519.15	0.05	1536.6	12.30
1531.72	28.63	1529.43	31.91	1540.1	34.71
1537.57	1.40	1529.72	34.51	1540.8	20.59
1541.54	0.25	1536.14	0.25	1547.4	0.33
1553.66	66.92	1538.59	0.07	1553.6	0.14
1555.23	59.03	1550.68	65.66	1576.0	2.46
1556.01	3.48	1551.95	74.36	1576.7	2.64
1570.79	0.02	1555.02	3.45	1597.6	6.68
1587.88	0.40	1568.34	0.47	1600.2	0.04
1588.86	0.50	1585.06	1.20	1616.7	2.82
1612.78	28.15	1587.30	2.41	1616.9	3.08
1613.51	2.94	1610.54	24.60	1617.5	0.99
1617.51	2.25	1611.55	1.31	1619.9	34.42
1617.99	3.22	1617.03	1.52	1659.8	0.45
1618.65	0.49	1617.36	2.33	1660.1	0.21
1631.50	9.95	1618.04	0.79	1660.4	0.31
1659.70	0.04	1627.07	15.70	1660.6	0.77
1660.21	0.07	1659.27	0.34	1719.3	114.04
1660.54	0.28	1659.66	0.39	1719.7	348.59
1660.62	0.37	1660.08	0.86	1720.0	170.91
1720.27	51.41	1660.47	0.35	1720.4	953.28
1720.49	117.53	1719.17	192.61	3162.8	21.80
1720.65	154.32	1719.42	128.73	3164.8	17.64
1720.96	1260.16	1719.95	424.57	3168.2	14.42

Table S17: Continuation.

Fe(III)Cl@TCPP		Fe(III)OH@TCPP		Fe(II)@TCPP	
Freq. (cm^{-1})	Int. (10^5 cm/mol)	Freq. (cm^{-1})	Int. (10^5 cm/mol)	Freq. (cm^{-1})	Int. (10^5 cm/mol)
3172.12	10.37	1720.35	839.35	3172.7	9.52
3172.25	10.29	3172.36	8.62	3173.6	10.34
3173.22	10.64	3172.56	9.35	3174.1	10.19
3173.38	10.19	3173.43	10.77	3174.7	9.01
3175.64	8.63	3173.89	10.78	3174.9	10.38
3176.68	7.27	3174.11	9.29	3200.0	16.43
3177.26	8.60	3174.85	4.52	3200.0	13.83
3179.48	6.51	3175.05	12.01	3201.2	15.31
3201.16	3.05	3175.43	7.20	3201.9	9.13
3201.21	13.55	3200.94	8.22	3202.0	3.99
3201.29	14.28	3200.98	18.95	3202.2	2.21
3201.40	20.70	3201.30	25.07	3202.9	1.58
3202.75	2.28	3201.34	7.95	3205.0	5.21
3203.16	2.04	3202.56	0.69	3261.4	0.31
3203.20	5.37	3202.83	4.56	3261.6	0.24
3204.48	5.00	3203.06	1.24	3261.9	0.37
3265.46	0.13	3203.44	6.34	3262.0	0.24
3265.54	0.13	3263.43	0.02	3279.3	3.84
3265.75	0.06	3264.01	0.10	3279.4	4.31
3266.60	0.32	3264.08	0.06	3279.8	5.50
3282.97	2.36	3264.49	0.02	3280.0	2.21
3283.08	3.77	3281.58	3.48		
3283.33	1.07	3281.97	2.41		
3284.82	1.93	3282.11	4.48		
		3282.32	0.75		
		3891.71	32.23		

Table S18: Calculated vibrational frequencies for Fe(II)(py-NMe-$PiPr_2$)Cl_2.

Fe(II)(py-NMe-$PiPr_2$)Cl_2	
Freq. (cm^{-1})	Int. (10^5 cm/mol)
27.91	0.56
39.18	0.41
51.56	0.84
73.55	0.58
78.76	4.31
83.02	0.48
99.67	3.16
105.73	5.54
109.00	5.45
123.32	0.33
139.29	1.09
155.86	0.37
180.10	2.11
191.65	3.06
202.44	0.07
210.35	3.57
222.21	0.81
235.97	0.02
248.06	0.04
253.10	0.12
256.32	0.20
280.67	3.51
313.80	1.00
321.59	5.97
325.37	23.16
345.95	1.88
356.48	5.25
395.27	69.60
397.79	22.58
412.36	2.49
435.85	5.02
437.47	3.54
477.58	24.33
535.78	5.69
558.35	6.08
654.96	2.57
680.34	14.08
682.82	16.17
720.62	27.11
763.56	24.57
795.84	34.01
869.72	0.77

Table S18: Continuation.

$Fe(II)(py\text{-}NMe\text{-}PiPr_2)Cl_2$	
Freq. (cm^{-1})	Int. (10^5 cm/mol)
901.42	93.92
905.88	13.29
910.87	2.90
933.79	1.06
937.79	6.39
982.22	0.12
986.96	0.24
990.84	0.24
1006.90	0.01
1029.98	6.50
1033.84	1.91
1068.93	1.98
1089.59	11.33
1108.74	1.03
1124.38	7.83
1126.41	2.75
1138.96	2.04
1159.07	16.39
1186.76	10.83
1188.43	2.54
1191.30	1.28
1218.10	29.08
1264.76	5.91
1280.64	10.73
1316.98	0.34
1320.50	7.07
1343.01	41.77
1347.48	36.68
1378.23	72.56
1395.83	0.97
1403.19	7.39
1418.14	13.33
1422.85	4.78
1456.62	18.90
1474.16	0.81
1480.66	2.49
1481.92	13.71
1484.68	15.08
1487.18	16.05
1490.03	4.18
1496.81	5.74
1499.84	25.28
1506.34	1.92
1507.92	11.86
1514.67	100.99
1531.32	213.95
1638.40	42.24
1675.48	232.43
3024.92	15.56
3028.20	5.35
3030.97	58.19
3046.17	25.82
3050.24	13.05
3051.26	24.50
3053.74	38.80
3090.22	17.03
3119.78	1.39
3121.44	7.57
3121.81	25.59
3127.53	13.78
3132.43	15.09
3142.81	15.82
3144.33	18.84
3156.01	8.28
3157.74	6.85
3200.08	5.22
3203.89	2.12
3239.05	2.33
3250.76	1.67

References

1. D. Z. Zee, T. D. Harris, *Chem. Sci.* **2020**, *11*, 5447–5452.

2. C. Koschnick, R. Stäglich, T. Scholz, M. W. Terban, A. von Mankowski, A. G. Savasci, F. Binder, A. Schökel, M. Etter, J. Nuss, R. Siegel, L. S. Germann, C. Ochsenfeld, R. E. Dinnebier, J. Senker, B. V. Lotsch, *Nat Commun* **2021**, *12*, 3099.

3. G. Kervern, G. Pintacuda, L. Emsley, *Chem. Phys. Lett.* **2007**, *435*, 157–162.

4. T. L. Hwang, P. C. M. van Zijl, M. Garwood, *J. Mag. Reson.* **1998**, *133*, 200–203.

5. J. Koppe, K. J. Sanders, T. C. Robinson, A. L. Lejeune, D. Proriol, S. Wegner, A. Purea, F. Engelke, R. J. Clément, C. P. Grey, A. J. Pell, G. Pintacuda, *Angew. Chem. Int. Ed.* **2025**, *1*, e202408704.

6. F. Neese, *Wiley Interdiscip. Rev. Comput. Mol. Sci.* **2011**, *2*, 73–78.

7. F. Neese, F. Wennmohs, U. Becker, C. Riplinger, *J. Chem. Phys.* **2020**, *152*, 224108.

8. F. Neese, *Faraday Discuss.* **2024**, *254*, 295–314.

9. F. Neese, *J. Comput. Chem.* **2023**, *44*, 381–396.

10. F. Neese, F. Wennmohs, A. Hansen, U. Becker, *Chem. Phys.* **2009**, *356*, 98–109.

11. F. Weigend, *Phys. Chem. Chem. Phys.* **2006**, *8*, 1057–1065.

12. G. L. Stoychev, A. A. Auer, F. Neese, *J. Chem. Theory Comput.* **2017**, *13*, 554–562.

13. C. Adamo, V. Barone, *J. Chem. Phys.* **1999**, *110*, 6158–6170.

14. E. Caldeweyher, S. Ehlert, A. Hansen, H. Neugebauer, S. Spicher, C. Bannwarth, S. Grimme, *J. Chem. Phys.* **2019**, *150*, 154122.

15. F. Weigend, R. Ahlrichs, *Phys. Chem. Chem. Phys.* **2005**, *7*, 3297–3305.

16. K. Wolinski, J. F. Hilton, P. Pulay, *J. Am. Chem. Soc.* **1990**, *112*, 8251–8260.

17. F. Jensen, *J. Chem. Theory Comput.* **2015**, *11*, 132–138.

18. S. Kozuch, J. M. L. Martin, *Phys. Chem. Chem. Phys.* **2011**, *13*, 20104–20107.

19. G. L. Stoychev, A. A. Auer, F. Neese, *J. Chem. Theory Comput.* **2018**, *14*, 4756–4771.

20. J. Gauss, *Chem. Phys. Lett.* **1992**, *191*, 132–138.

21. P. Pinski, C. Riplinger, E. Valeev, F. Neese, *J. Chem. Phys.* **2015**, *143*, 034108.

22. G. L. Stoychev, A. A. Auer, J. Gauss, F. Neese, *J. Chem. Phys.* **2021**, *154*, 164110.

23. A. Antušek, K. Jackowski, M. Jaszuński, W. Makulski, M. Wilczek, *Chem. Phys. Lett.* **2005**, *411*, 111–116.

24. A. Jaworski, N. Hedin, *Phys. Chem. Chem. Phys.* **2021**, *23*, 21554–21567.

25. A. Jaworski, N. Hedin, *Phys. Chem. Chem. Phys.* **2022**, *24*, 15230–15244.

26. R. A. Kendall, T. H. Dunning, R. J. Harrison, *J. Chem. Phys.* **1992**, *96*, 6796.

27. K. A. Peterson, T. H. Dunning, *J. Chem. Phys.* **2002**, *117*, 10548.

28. V. Barone, *Recent Advances in Density Functional Methods, Part I*, World Scientific Publ. Co., Singapore, **1995**.

29. N. Rega, M. Cossi, V. Barone, *J. Chem. Phys.* **1996**, *105*, 11060.

30. N. Rega, M. Cossi, V. Barone, *J. Am. Chem. Soc.* **1997**, *119*, 12960–12967.

31. N. Rega, M. Cossi, V. Barone, *J. Am. Chem. Soc.* **1998**, *120*, 5723–5732.

32. F. Weigend, A. Köhn, C. Hättig, *J. Chem. Phys.* **2002**, *116*, 3175–3183.

33. C. Hättig, *Phys. Chem. Chem. Phys.* **2005**, *7*, 59–66.

34. T. H. Dunning, *J. Chem. Phys.* **1989**, *90*, 1007–1023.

35. D. E. Woon, T. H. Dunning, *J. Chem. Phys.* **1993**, *98*, 1358–1371.

36. A. K. Wilson, D. E. Woon, K. A. Peterson, T. H. Dunning, *J. Chem. Phys.* **1999**, *110*, 7667.

37. B. A. Hess, *Phys. Rev. A* **1985**, *32*, 756.

38. B. A. Hess, *Phys. Rev. A* **1986**, *333*, 3742.

39. G. Jansen, B. A. Hess, *Phys. Rev. A* **1989**, *39*, 6016.

40. L. Visscher, K. G. Dyall, *Atom. Data Nucl. Data Tabl.* **1997**, *67*, 207–224.

41. D. Ganyushin, F. Neese, *J. Chem. Phys.* **2013**, *138*, 104113.

42. S. Jiang, D. Maganas, N. Levesanos, E. Ferentinos, S. Haas, K. Thirunavukkuarasu, J. Krzystek, M. Dressel, L. Bogani, F. Neese, *J. Am. Chem. Soc.* **2015**, *137*, 12923–12928.

43. N. B. Balabanov, K. A. Peterson, *J. Chem. Phys.* **2005**, *123*, 64107.

44. W. A. de Jong, R. J. Harrison, D. A. Dixon, *J. Chem. Phys.* **2001**, *114*, 48.

45. A. Soncini, W. Van den Heuvel, *J. Chem. Phys.* **2013**, *138*, 021103.

46. W. Van den Heuvel, A. Soncini, *J. Chem. Phys.* **2013**, *138*, 054113.

47. N. F. Ramsey, *Phys. Rev.* **1950**, *77*, 567.

48. N. F. Ramsey, *Phys. Rev.* **1950**, *78*, 699–703.

49. N. F. Ramsey, *Phys. Rev.* **1951**, *83*, 540–541.

50. N. F. Ramsey, *Phys. Rev.* **1952**, *86*, 243–246.

51. N. F. Ramsey, *Phys. Rev.* **1953**, *91*, 303–307.

52. P. Pyykkö, *Theor. Chem. Acc.* **2000**, *103*, 214–216.

53. T. Helgaker, M. Jaszuński, K. Ruud, *Chem. Rev.* **1999**, *99*, 293–352.

54. J. Vaara, *Phys. Chem. Chem. Phys.* **2007**, *77*, 5399–5418.

55. A. A. Auer, V. A. Tran, B. Sharma, G. L. Stoychev, D. Marx, F. Neese, *Mol. Phys.* **2020**, *118*, e1797916.

56. R. J. Abraham, G. E. Hawkes, M. F. Hudson, K. M. Smith, *J. Chem. Soc. Perkin Trans.* **1975**, *2*, 204–211.

57. A. Dittmer, G. L. Stoychev, D. Maganas, A. A. Auer, F. Neese, *J. Chem. Theory Comput.* **2020**, *16*, 6950–6967.

58. C. J. Medforth, M. O. Senge, K. M. Smith, L. D. Sparks, J. A. Shelnutt, *J. Am. Chem. Soc.* **1992**, *114*, 9859–9869.

59. L. D. Sparks, C. J. Medforth, M. S. Park, J. R. Chamberlain, M. R. Ondrias, M. O. Senge, K. M. Smith, J. A. Shelnutt, *J. Am. Chem. Soc.* **1993**, *115*, 581–592.

60. H. Ryeng, A. Ghosh, *J. Am. Chem. Soc.* **2002**, *124*, 8099–8103.

61. M. Nakamura, *Coord. Chem. Rev.* **2006**, *250*, 2271–2294.

62. A. Bertarello, L. Benda, K. J. Sanders, A. J. Pell, M. J. Knight, V. Pelmenschikov, L. Gonnelli, I. C. Felli, M. Kaupp, L. Emsley, R. Pierattelli, G. Pintacuda, *J. Am. Chem. Soc.* **2020**, *142*, 16757–16765.

63. M. Munzarová, M. Kaupp, *J. Phys. Chem. A* **1999**, *103*, 9966–9983.

64. F. Neese, *J. Chem. Phys.* **2003**, *118*, 3939.

65. M. Kaupp, A. V. Arbuznikov, A. Heßelmann, A. Görling, *J. Chem. Phys.* **2010**, *132*, 184107.

66. C. J. Schattenberg, T. M. Maier, M. Kaupp, *J. Chem. Theory Comput.* **2018**, *14*, 5653–5672.

67. A. Pyykkönen, A. Wodyński, M. Kaupp, J. Vaara, *Phys. Chem. Chem. Phys.* **2025**, *27*, 18887–18900.

68. R. J. Bartlett, M. Musiał, *Rev. Mod. Phys.* **2007**, *79*, 291–352.

69. F. Neese, M. Atanasov, G. Bistoni, D. Maganas, S. Ye, *J. Am. Chem. Soc.* **2019**, *141*, 2814–2824.

70. S. A. Perera, J. D. Watts, R. J. Bartlett, *J. Chem. Phys.* **1994**, *100*, 1425.

71. M. Saitow, U. Becker, C. Riplinger, E. F. Valeev, F. Neese, *J. Chem. Phys.* **2017**, *146*, 164105.

72. M. Saitow, F. Neese, *J. Chem. Phys.* **2018**, *149*, 034104.

73. Y. Guo, C. Riplinger, D. G. Liakos, U. Becker, M. Saitow, F. Neese, *J. Chem. Phys.* **2020**, *152*, 024116.

74. I. Zhang, A. Grüneis, *Front. Mater.* **2019**, *6*, 123.

75. R. Calvo, M. Passeggi, *Physics Letters* **1970**, *31A*, 7.

76. J. Minge, J. Weil, *J. Phys. Chem. Solids* **1989**, *50*, 997–1001.

77. J. Blahut, L. Benda, A. L. Lejeune, K. J. Sanders, E. Burcher, B. Jeanneau, D. Proriol, L. Catita, P. A. R. Breuil, A. J. Quoineaud, A. A. Pell, G. Pintacuda, *RSC Adv.* **2021**, *11*, 29870–29876.
